# Highly tunable bimane-based fluorescent probes: design, synthesis, and application as a selective amyloid binding dye†

**DOI:** 10.1039/d4sc00024b

**Published:** 2024-03-26

**Authors:** Yarra Venkatesh, Nicholas P. Marotta, Virginia M.-Y. Lee, E. James Petersson

**Affiliations:** a Department of Chemistry, University of Pennsylvania 231 South 34th Street Philadelphia PA 19104 USA ejpetersson@sas.upenn.edu; b Graduate Group in Biochemistry and Molecular Biophysics, Perelman School of Medicine, University of Pennsylvania 421 Curie Boulevard Philadelphia PA 19104 USA; c Department of Pathology and Laboratory Medicine, Center for Neurodegenerative Disease Research, University of Pennsylvania 3600 Spruce Street Philadelphia PA 19104 USA

## Abstract

Small molecule fluorescent probes are indispensable tools for a broad range of biological applications. Despite many probes being available, there is still a need for probes where photophysical properties and biological selectivity can be tuned as desired. Here, we report the rational design and synthesis of a palette of fluorescent probes based on the underexplored bimane scaffold. The newly developed probes with varied electronic properties show tunable absorption and emission in the visible region with large Stokes shifts. Probes featuring electron-donating groups exhibit rotor effects that are sensitive to polarity and viscosity by “intramolecular charge transfer” (ICT) and twisted intramolecular charge transfer (TICT) mechanisms, respectively. These properties enable their application as “turn-on” fluorescent probes to detect fibrillar aggregates of the α-synuclein (αS) protein that are a hallmark of Parkinson's disease (PD). One probe shows selective binding to αS fibrils relative to soluble proteins in cell lysates and amyloid fibrils of tau and amyloid-β. Finally, we demonstrate the diagnostic potential of the probe in selectively detecting αS fibrils amplified from PD with dementia (PDD) patient samples.

## Abstract 2

Rational design of environmentally-sensitive bimane probes with tunable properties holds promise for studying neurodegenerative disease.

## Graphical abstract

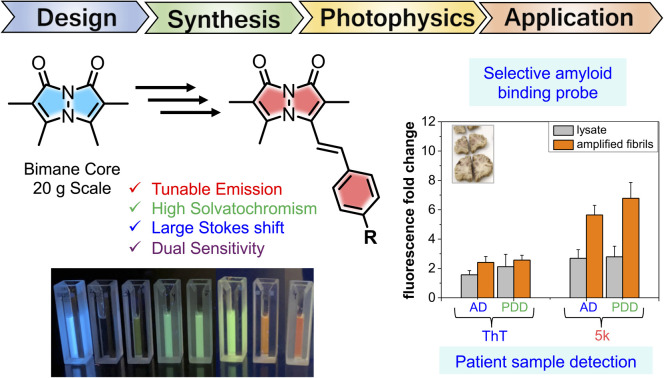

## Introduction

Fluorescence spectroscopy has become a pivotal method in the life sciences, spanning fundamental research to clinical applications. Fluorescence-based technologies characterize protein/protein, protein/ligand, and membrane/biomolecule interactions which play crucial roles in the regulation of cellular pathways.^1–3^ In this context, small molecule based fluorescent probes have significant advantages over protein-based probes in optical imaging and analytical sensing due to their small size (minimizing disruption to the target biomolecule). Despite the plethora of developed fluorescent probes, their structural modifications often rely on a diminutive set of classical “core” dyes such as coumarin, fluorescein, boron dipyrromethene (BODIPY), or cyanine,^2,4^ emphasizing the importance of core fluorophore modularity and highlighting the need for novel core scaffolds in probe design. Particularly in biological systems, the demand persists for “ideal” probes that exhibit drug-like uptake with high biomolecule selectivity and photophysical properties tailored to a given fluorescence assay.

Recently, molecular rotor-based, photoisomerizable, and/or aggregation-induced emission (AIE) fluorophores are emerging classes of molecules that rotate along specific bonds in the excited state. These types of fluorophores offer unique advantages in terms of sensitivity and specificity and are powerful tools to study amyloid fibrils accumulating in cells or tissue samples from patients with Alzheimer's disease (AD), Parkinson's disease (PD), and many other neurodegenerative disorders.^5^ The design and application of such probes often involve careful consideration of the fluorophore's properties for high selectivity and sensitivity in detecting protein aggregation within complex biological environments. Rotor-based fluorophores, a major family of these molecules, are widely employed as fluorogenic probes due to their enhanced fluorescence in viscous environments, resulting from restriction of bond rotation. Thioflavin T (ThT), a well-known rotor-based dye, has been extensively used for staining amyloid fibrils, displaying increased fluorescence upon binding. However, ThT has limitations, including a small Stokes shift, high background fluorescence, low protein specificity, and poor selectivity for fibrils over other types of protein aggregates, as well as poor cellular uptake.^6,7^ These drawbacks have driven the search for improved fluorogenic amyloid dyes incorporating rotor-based mechanisms, leading to improved photophysical properties such as: larger Stokes shifts, greater environmental sensitivity, enhanced selectivity, and reduced background fluorescence.^8–10^

**Scheme 1:**
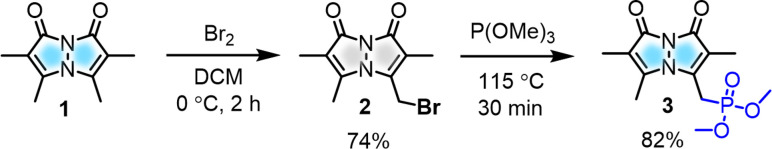
Synthesis of key bimane precursor.

**Scheme 2:**
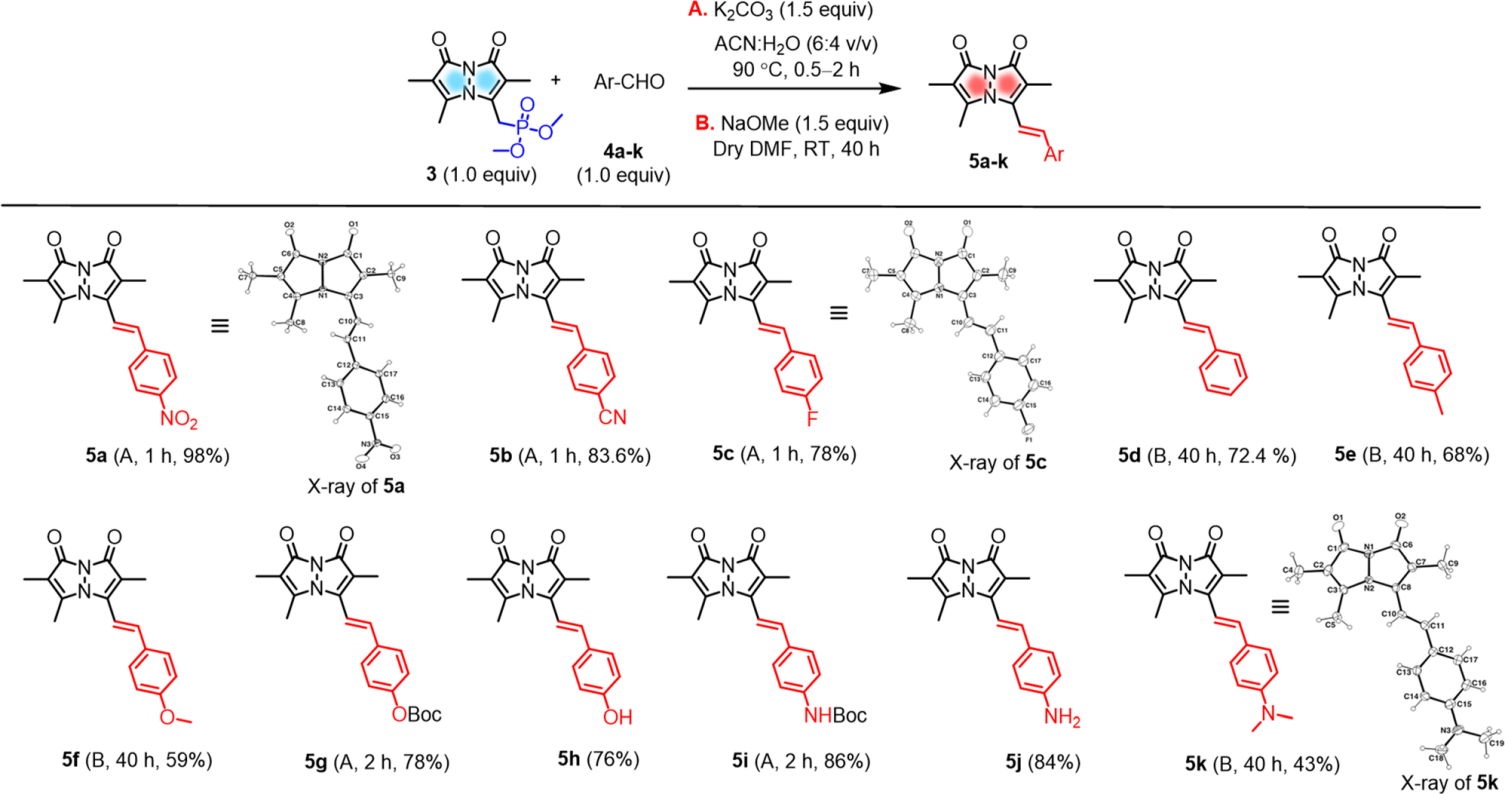
Aryl aldehyde substrate scope for H–W–E reactions (Boc = *tert*-butoxycarbonyl).

As a potential scaffold for such amyloid dyes, bimane is a highly attractive structure to its small size and relatively low toxicity.^11–17^ Bimane was introduced by Kosower and coworkers nearly four decades ago,^13,14^, and is widely used for *in vitro* protein labeling as cysteine-reactive monobromobimane (Scheme 1, 2).^12,15–17^ However, there have been some applications in cells, and it has been shown that a *syn*-bimane adduct of α-aminobutyric acid can cross the blood–brain barrier in rodents.^18^ Unfortunately, the potential for practical applications of bimane derivatives has thus far been limited due to synthetic routes requiring hazardous reagents that can be difficult to handle. To prepare the bimane core, the key intermediate *i.e.*, chloropyrazolinone is typically synthesized using chlorine gas. Recently, Neogi *et al.*, developed an alternative method to access the intermediate, using trichloroisocyanuric acid (TCCA), an easy-to-handle solid chlorination reagent.^19^ Recent advances in synthesis led by the Grynszpan and Levine groups have facilitated the formation of a complex of β-cyclodextrin with *syn*-bimane/bimane-ditriazole, used for the detection of cobalt and iodine, respectively.^20,21^ Bimane-ditriazole and Cu(ii) or boronate ester-functionalized bimane are extremely sensitive to the detection of trace amounts of water.^22^ In addition, bimane has the capability to act as an O-donor ligand in metal-bimane complexes, including those with Pd(ii), Na(i), and Li(i).^23^ However, with the exception of a few derivatives,^23–25^ exploration of bimanes in complex biological applications has been limited due to their UV absorption and blue fluorescence.

To pursue our goal of improving photophysical properties and advancing the development of next-generation fluorophores, we present a small but highly tunable bimane scaffold accessible through key intermediate 3 in Scheme 1. This scaffold is designed to be compatible with the rational design and synthesis of libraries of fluorescent probes (5a–k). The newly synthesized probes offer the capability to design photophysical properties, including (1) visible light excitation, (2) a 3- to 4-fold increase in molar extinction coefficient (*ε*), (3) highly tunable emission spanning from blue to red color, and (4) large (∼200 nm) Stokes shifts. Remarkably, derivatives featuring electron-donating groups (EDGs) demonstrate sensitivity to both polarity and viscosity through intramolecular charge transfer (ICT) and twisted intramolecular charge transfer (TICT) mechanisms, respectively. Probes with EDGs (5j and 5k) showcase applications as “turn on” fluorescent probes for the selective binding to the α-synuclein (αS) protein that aggregates to form amyloid fibrils in PD and related neurodegenerative disorders.^20,21^ Furthermore, 5k demonstrates selective binding to αS fibrils over (1) αS monomers, (2) cellular proteins in lysates, and (3) amyloid fibrils of other proteins such as tau and amyloid-β. Finally, we demonstrate the diagnostic potential of 5k by selectively detecting polymorphs or “strains” of αS fibrils from PD with dementia (PDD) patient samples.

## Results and discussion

### Rational design of bimane scaffold

To modulate the photophysical properties of the bimane core, we chose to derivatize at the 3-position, since studies with the commonly used 3-bromomethyl bimane 2 have shown that conversion to a thioether by reaction with thiols leads to blue-shifted absorption and fluorescence turn-on.^12^ Hence, there was a precedent for electronic communication with the bimane core *via* substituents at the 3-position. To access bimane derivatives, we synthesized bromide 2 and converted it to phosphonate ester 3 (Scheme 1) for use in Horner–Wadsworth–Emmons (H–W–E) reactions with diverse aldehydes, one of the most useful methods for C=C bond formation with predominantly *E* configuration (Scheme 2).

Initially, the gram-scale synthesis of the bimane core 1 was achieved in three sequential steps with modifications of a previously established milligram scale procedure.^19^ It involves (1) condensation under sonication (96.4% yield, no chromatography), (2) chlorination by TCCA (84% yield, no chromatography), and (3) cyclization under heterogeneous basic conditions (71% yield), resulting in improved yields, as depicted in the ESI (see ESI, pages S4 and S5†). Then, the key precursor methyl bimane phosphonate 3 was synthesized in two steps as shown in Scheme 1, bromination followed by an Arbuzov reaction. *syn*-Bimane 1 was treated with a bromine solution to afford bromobimane 2 in 74% yield. Subsequent reaction with neat trimethyl phosphite led to the formation of compound 3 in 82% yield (Fig. S29, see ESI†).

### Synthesis of arylated bimane probes

A set of commercially available aryl aldehydes with a range of electron-withdrawing to electron-donating *para*-substituents were subjected to the H–W–E reaction with 3 under condition A, K_2_CO_3_ in an acetonitrile (ACN)/H_2_O mixture (6 : 4 v/v) at reflux (Scheme 2). All the electron-poor aryl aldehydes (4a–c) afforded good to excellent isolated yields (>78%) of 5a–c with short reaction times, but electron-rich aryl aldehyde 4k afforded a low isolated yield (<10%). Reactions of protected aldehydes 4g and 4i proceeded with condition A to give excellent yields of 5g and 5i. Next, deprotection of 5g and 5i by trifluoroacetic acid (TFA) yielded free alcohol and amine derivatives 5h and 5j.

To improve conversions for electron-rich aldehydes, we optimized the reaction conditions to prepare the dimethylamino analogue 5k as a model reaction by varying the base, solvent, and temperature as shown in Table 1. Optimized condition B using NaOMe as a base in dry DMF at room temperature (entry 4 in Table 1, ESI pages S8 and S9†) was then applied to other electron-rich aryl aldehydes (4d–f, 4k), improving the yields to 43–72% of 5d–f and 5k. All probes were characterized by ^1^H, ^13^C NMR and high-resolution mass spectrometry (Fig. S30–S39, see ESI†).

**Table 1:** Screening of H–W–E reaction conditions^a^

Entry	Solvent	Base	Temp.	Time	Yield (%)
1	ACN/H_2_O	K_2_CO_3_	RT	40 h	2
2	ACN/H_2_O	K_2_CO_3_	90 °C	2 h	9
3	DMF	KO*t*Bu	RT	40 h	27
**4**	**DMF**	**NaOMe**	**RT**	**40 h**	**46**
5	DMF	NaOMe	60 °C	40 h	38
6	DMSO	KO*t*Bu	RT	40 h	4
7	DMSO	NaOMe	RT	40 h	31
8	DMSO	NaOMe	60 °C	40 h	12

a Reaction conditions: 3 (1 equiv.), aryl aldehyde 4k (1 equiv.), KO*t*Bu (1.5 equiv.), NaOMe (1.5 equiv.), K_2_CO_3_ (1.5 equiv.), yields based on chromatogram peak areas.

### Characterization by X-ray crystallography

The solid-state structures of 5a, 5c, and 5k were also characterized by single-crystal X-ray crystallography and are shown in Scheme 2 (for the details of full structure determination see ESI pages S14–S24†). Probes 5a and 5c were crystallized in space groups *P*2_1_ and *Fdd*2, but 5k cocrystallized with methanol solvent molecules in space group *P*2_1_/*c*. The bond lengths and bond angles of the pyrazolinone rings of 5a, 5c, and 5k show little variation between these three probes (see ESI Tables S2, S3, S5, S6, S8 and S9†). Interestingly, the pyrazolinone rings of 5a (R = NO_2_) deviate from planarity and exhibit a butterfly bend of their bicyclic framework around the N–N bond, with *φ* = 128.21(15) for its 8-membered ring compared to 5c (R = F), showing planarity with *φ* = 140.38(18). Here too, 5k (R = NMe_2_) showed a slight bend compared to 5c with *φ* = 139.1(3) (see ESI Table S10†). The crystal structures of 5a, 5c, and 5k show intermolecular stacking of these molecules, as shown in the Table S11 (see ESI†), this could be expected due to the existence of highly conjugated π–π interactions. In the case of 5a, the molecules are stacked face-to-face, with a slippage of 3.86 Å between neighboring molecules, while 5c and 5k are packed head to tail and have varied distances from neighboring molecules. In the case of 5k, other notable intermolecular interactions such as hydrogen bonding between the bimane oxygen atom and the hydrogen atom of the cocrystallized methanol molecules were observed.

### Substituent effects on photophysical properties

**Fig. 1:**
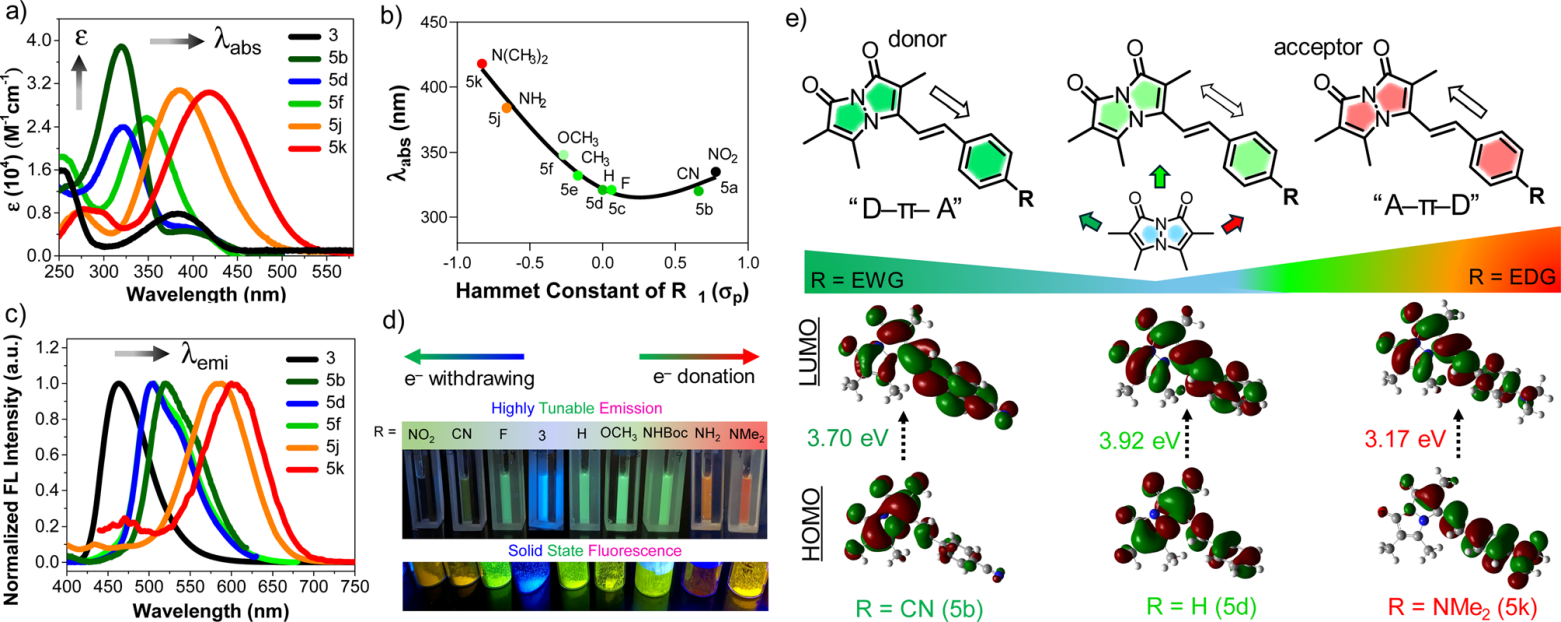
Highly tunable photophysical properties of bimane probes by varying substituent effects. (a) Absorption spectra of selected bimane probes 5b, 5d, 5f, 5j, 5k and 3. Structure–photophysical property relationship of 5a–f and 5j, 5k; (b) correlation between absorption maximum (*λ*_abs_) and Hammett constant (*σ*_p_) of R substituents in styryl bimanes. (c) Normalized and smoothed fluorescence spectra of selected bimane probes 5b, 5d, 5f, 5j, 5k and 3, measured with excitation at their optimal max absorption. (d) Top: Photographs of the tunable emission in ACN/PBS solution, Bottom: solid state fluorescence under handheld UV lamp at 365 nm; (e) Top: schematic representation of the bimane probes showing “push–pull” D–π–A or A–π–D type systems based on the aryl substituent R; D: donor, A: acceptor, Bottom: HOMO and LUMO of 5b (R = CN), 5d (R = H), and 5k (R = NMe_2_), showing the movement in electron density upon excitation from S_0_ to S_1_. Final concentration of probes is 25 μM in ACN and PBS buffer (50 : 50 v/v).

After successful synthesis of a styryl bimane probe library with varied substitution, we examined the photophysical properties of each probe in 50 : 50 ACN/phosphate buffered saline (PBS). As shown in absorption spectra Fig. 1a, a more electron-donating aryl substitution generated a more red-shifted absorption maximum (*λ*_abs_) compared to *λ*_abs_ of parent compound 3, whereas a more electron-withdrawing aryl substitution generated a blue-shifted *λ*_abs_, suggesting that there is significant electronic communication through the styryl bimane scaffold. In addition to the *λ*_abs_ shifts, we also observed a 3–5-fold increase in molar absorptivity (*ε*) relative to 3, which is attributed to the increase in π-conjugation through the styryl group. These results encouraged us to establish systematic guidelines to aid the deliberate selection of substituents for future bimane derivatives. In this context, we analyzed 5a–f, 5j, and 5k in terms of the Hammett substituent constant (*σ*_p_) for the *para*-functional group on the phenyl ring (Fig. 1b).^12^

Electron-donation showed a positive correlation between the *σ*_p_ and *λ*_abs_, but electron-withdrawing groups changed the slope of the Hammett plot, indicating a change in mechanism. This analysis was further supported by HOMO–LUMO density functional theory (DFT) calculations of 5b (R = CN), 5d (R = H), and 5k (R = NMe_2_) (Fig. 1e). In the case of 5k, the movement of electron density from the *N*,*N*-dimethylamino group of 5k to the bimane core upon excitation from S_0_ to S_1_ (*λ*_abs_ = 418 nm, *E* = 3.17 eV). For 5d there is no significant change in electron density movement upon excitation from S_0_ to S_1_ (*λ*_abs_ = 321 nm, *E* = 3.92 eV). In contrast, for 5b the movement of electron density from the bimane group to the electron withdrawing group on the phenyl ring is seen upon excitation from *S*_0_ to *S*_1_ (*λ*_abs_ = 320 nm, *E* = 3.70 eV). Again, for 5a (R = NO_2_), we observed redshifted absorption at *λ*_abs_ = 335 nm with *E* = 3.32 eV. Based on this combination of experimental results and computational analysis, in Fig. 1e and pages S28–S32 in ESI,† we illustrate the potential to design “push–pull” systems of both the donor–spacer–acceptor (D–π–A) and acceptor–spacer–donor (A–π–D) variety. This design is facilitated by the bimane core, which acts as either a donor or acceptor, its functionality depending on the specific aryl substituent *R*.

Next, the emission spectrum of each compound was measured in ACN/PBS (50 : 50) with excitation at *λ*_abs_ (Table 2). As shown in Fig. 1c, analysis of the emission spectra of selected bimane derivatives 5b, 5d, 5f, 5j, 5k and 3 with different substituents showed tunable emission with a significant bathochromic shift of the emission maximum (*λ*_em_) compared to the parent phosphonate 3 (*λ*_em_ = 464 nm). This tunable emission is seen both in solution and the solid state (Fig. 1d). Notably, derivatives 5a–c, featuring electron-withdrawing substituents on the aryl ring, exhibited green emission within the range of 502–520 nm. In contrast, derivatives 5j (R = NH_2_, *λ*_em_ = 583 nm) and 5k (R = NMe_2_, *λ*_em_ = 604 nm), possessing electron-donating substituents, displayed larger bathochromic shifts in *λ*_em_. Further comparison of 5h (R = NHBoc, *λ*_em_ = 505 nm) and 5i (R = NH_2_, *λ*_em_ = 583 nm), which differ only by removal of the *tert*-butoxycarbonyl (Boc) group, highlighted the influence of the amine lone pair on the emission wavelength shift (Table 2). Taken together, these results further support the idea that the tunable emission mainly stems from the design of “push–pull” D–π–A or A–π–D systems. Moreover, we also calculated the Stokes shifts for 5a–k and observed values ranging from 133 to 217 nm (Table 2). These interesting fluorescent properties of styryl bimanes, such as high tunability and large Stokes shift, will make them valuable probes for a broad spectrum of biological applications.

**Table 2:** Photophysical properties of bimane derivatives 5a–f, 5h–k and 3 in ACN/PBS buffer (50 : 50 v/v)

Entry	Substituent (Ph-R)	Absorbance *λ*_abs_^a^ (nm)	Emission *λ*_em_^b^ (nm)	*ε* c (M^−1^ cm^−1^)	Stokes shift^d^ (nm)	Fluorescence QY		Fluorescence lifetime (ns)	HOMO–LUMO gap^g^ (eV)
						ACN/PBS^e^ (%)	ACN^f^ (%)		
3	—	384	464	7902	80	64.6	77.7	2.32	—
5a	NO_2_	335	468^h^	36 955	133	0.1	0.2	—	3.315
5b	CN	320	520	38 995	200	0.8	1.4	—	3.696
5c	F	321	502	23 796	181	2.5	2.8	1.07	3.912
5d	H	321	503	23 959	182	2.5	3.1	1.02	3.915
5e	CH_3_	332	503	21 164	171	3.0	3.4	1.28	3.881
5f	OCH_3_	348	504	25 659	156	3.3	3.5	1.31	3.726
5h	OH	354	506	31 514	152	2.3	3.1	—	3.772
5i	NH(Boc)	356	505	34 826	149	3.4	3.4	—	—
5j	NH_2_	385	583	30 834	198	0.7	1.5	—	3.429
5k	NMe_2_	418	604	30 420	186	0.5	7.5	—	3.172

a Maximum absorption wavelength.

b Maximum emission wavelength.

c Molar absorption coefficients at maximum absorption wavelength.

d Difference between maximum absorption wavelength and maximum emission wavelength.

e Fluorescence quantum yield (error limit within ±5). For all probes, final concentration is 25 μM in ACN/PBS buffer at pH 7.4.

f Fluorescence quantum yield in acetonitrile (ACN) solvent.

g HOMO and LUMO energy levels of probes in water were calculated using APF-D/6-311+G (2d, p) DFT calculations.

h The red-shifted emission band is presumably quenched by the nitro group through a PET process, leading to the appearance of the 468 nm band corresponding to native bimane fluorescence.

Measurement of the fluorescence quantum yield (QY) of each compound in ACN/PBS revealed dramatic changes for the styryl bimanes 5a–k (0.1–3.4%) compared to parent phosphonate 3 (64.6%). The changes in QY are also reflected in shortened fluorescent lifetimes (*τ*) for the derivatives (1.02–1.31 ns) compared to 3 (2.32 ns) (Tables 2, S13 and Fig. S10 in ESI†). In pure ACN, *ε* values were not dramatically affected, but the QY of some compounds increased significantly, particularly tertiary amine probe 5k, with a QY of 7.5% in ACN. Notably, primary amine 5j did not experience as large an increase in QY (2%) (Tables 2, S12 and Fig. S5 ESI†). The large effect of solvent on QY prompted us to examine the environmental sensitivity of the probes.

### Environmental sensitivity of probes: polarity and viscosity

**Fig. 2:**
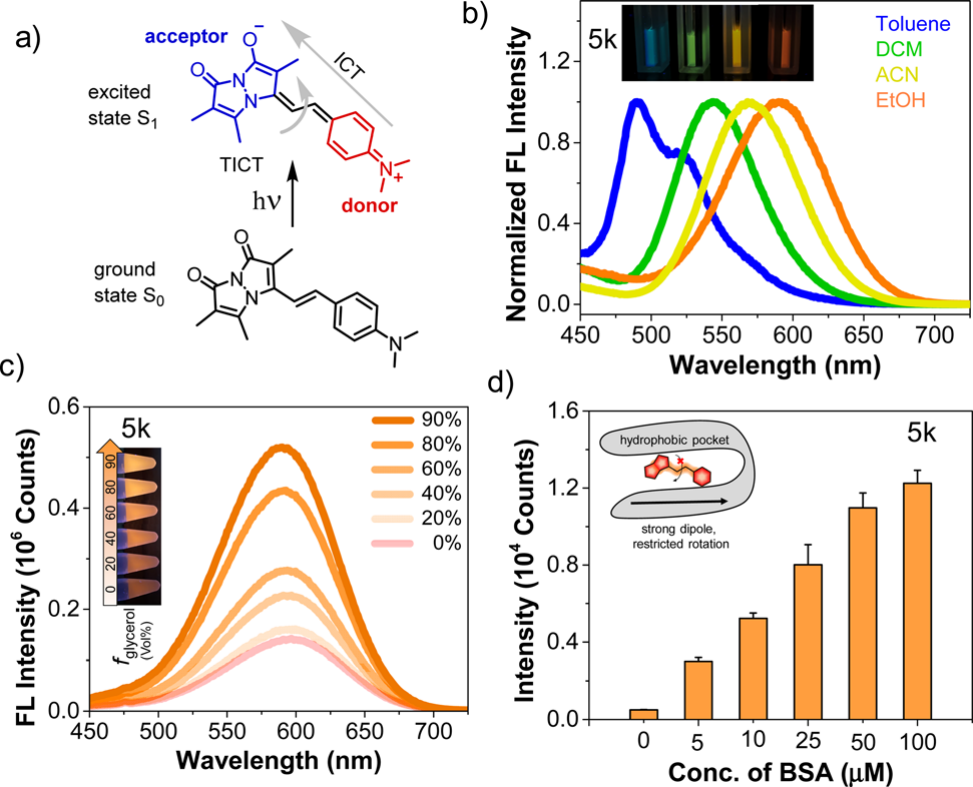
Dual sensitivity in polarity and viscosity of a solvatochromic fluorophore 5k. (a) D–π–A system shows the phenomenon of ICT from the donor to the acceptor in the excited state and excited fluorophore could either return to the ground state through fluorescence or through internal rotation which leads to non-radiative TICT. (b) Solvatochromism of 5k in organic solvents with different polarity. 5k was prepared at 25 μM in indicated solvents. (c) Viscosity sensitivity of 5k with varying the glycerol concentration in ethylene glycol. Inset: Tubes with varying glycerol % illuminated with a 365 nm handheld UV lamp. (d) Fluorescence intensity of probe 5k (1 μM) in the presence of varying concentrations of BSA protein. Inset: Schematic representation of the fluorescence “turn on” mechanism through rotational restriction upon binding to the hydrophobic pockets on the protein surface. *λ*_ex_/*λ*_em_ = 463/580 nm for 5k. Error bars represent SD of 3 measurements.

We investigated solvatochromism more generally, obtaining emission spectra in four organic solvents with different polarity: nonpolar aprotic (toluene, DCM), polar aprotic (ACN), and polar protic (EtOH). As illustrated for 5k (R = NMe_2_) in Fig. 2b, the probe showed a high level of solvatochromism, with a red-shift in more polar solvents and emission spanning 490–590 nm. Other derivatives having electron-donating groups (5j, R = NH_2_) showed sensitivity to solvent polarity, while parent compound 3 showed no significant environmental sensitivity (ESI, Fig. S6†). Probe 5b with an electron-withdrawing group (R = CN) and 5d (R = H) displayed no significant solvatochromism (ESI, Fig. S6†). Overall, these results suggest that probes with electron-donating groups have the ability to exhibit solvatochromism.

We also studied pH effects (ESI, Fig. S9†) and found that although some compounds exhibited pH effects, these were not significant in physiological pH ranges. For example, 5h (R = OH) has red-shifted absorption at pH 11 with a reduced QY, but no shift in emission. Probe 5j (R = NH_2_) has blue-shifted absorption and emission at pH 2. Taken together with the solvent polarity effects, these data show that environmental effects are tunable based on the aryl substituent and motivate the use of amine-containing derivatives as probes of the local environment on a protein surface or in a cellular compartment. The lack of pH sensitivity in the physiological range or changes in protic solvents implies that these effects are modulated more by the general polarity of the environment than by specific hydrogen bonding or protonation. Moreover, in nonpolar solvents 5k showed a blue-shifted emission with enhanced intensity, while in polar solvents it displayed a red-shifted emission accompanied by a reduction in intensity. We hypothesized that styryl bimane probes in polar solvents undergo TICT through non-radiative pathways, resulting from free rotation around the stryryl linker that connects the π-systems in the excited state.

To investigate this, we recorded the emission spectra of 5k in various binary mixtures of ethylene glycol and glycerol (Fig. 2c). Since ethylene glycol and glycerol have similar polarities, the fluorescence intensity measured in mixtures of the two solvents should be influenced solely by the solvent viscosity. The increased viscosity of glycerol serves to restrict free rotation leading to non-radiative TICT states. A 3.7-fold increase in fluorescence intensity with increased % glycerol indicates that, indeed, rotational restriction planarizes the π system, increasing radiative emission. We supposed that the combination of solvatochromic and rotor effects should lead to a dramatic fluorescence turn-on when binding occurs within a hydrophobic cavity on a protein. Indeed, testing 5k with bovine serum albumin (BSA) showed that turn-on was possible as shown in Fig. 2d, prompting us to test binding to targets of more physiological significance.

### Bimane derivatives as “turn-on” binding probes for αS fibrils

**Fig. 3:**
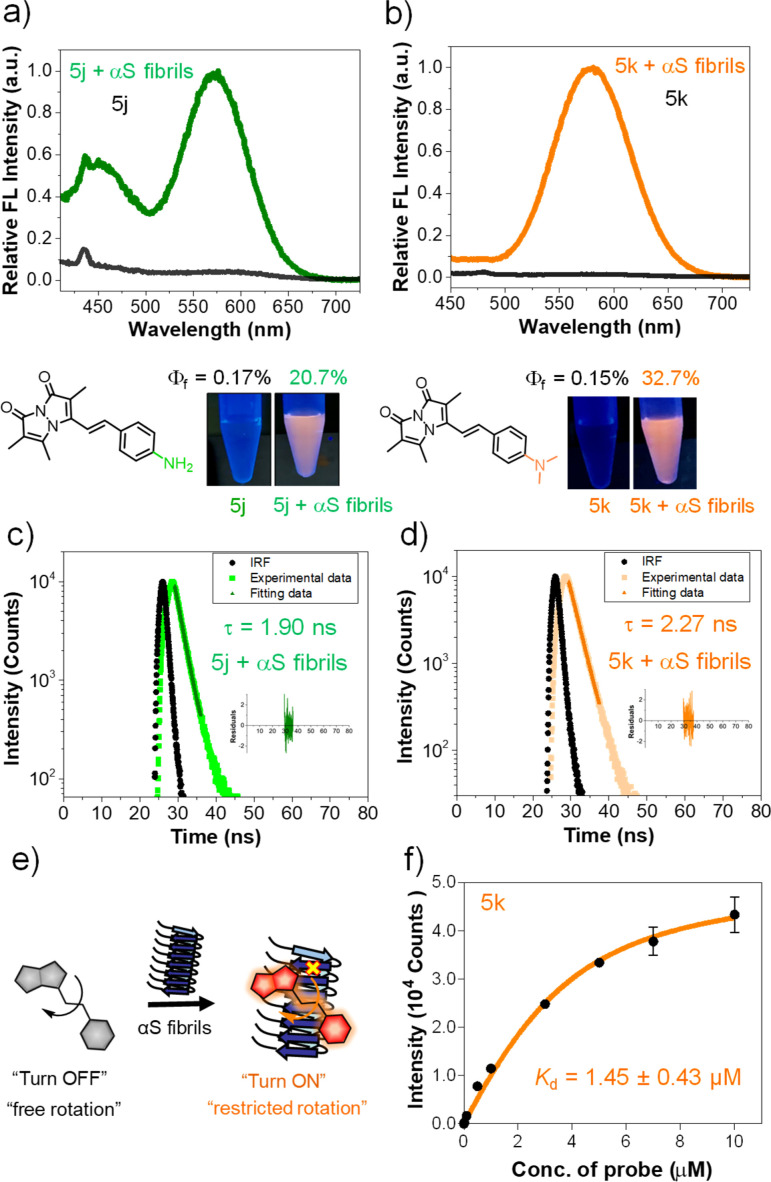
Fluorogenic probes for the detection of αS fibrils. Fluorescence spectral changes of probes in the presence and absence of αS PFFs (a) 5j and (b) 5k; bottom: structure and photographs of the probes with and without αS PFFs under handheld UV light at 365 nm. Concentrations of dyes and PFFs were 10 and 50 μM, respectively. (c and d) Fluorescence lifetime (*τ*) measurements of 5j and 5k with 100 μM αS PFFs by time-correlated single photon counting (TCSPC) technique and lifetime was not detected for probe alone. (e) Schematic representation of the fluorescence “turn on” mechanism by rotational restriction upon binding to αS-PFFs. (f) Dissociation constant (*K*_d_) determination of 5k (10 nM–10 μM) with 100 μM αS PFFs (see the ESI† for the *K*_d_ fitting parameters and constraints). *λ*_ex_/*λ*_em_ = 435/575 nm for 5j and 463/580 nm for 5k. Error bars represent SD of 3 measurements.

The intriguing photophysical features of our bimane probes, such as solvatochromism and viscosity sensitivity exhibited by 5j and 5k, along with their structural resemblance to amyloid binding dyes currently under investigation in our laboratory and others,^25–28^ prompted us to explore their potential application in amyloid sensing. We initially focused on the αS protein that aggregates to form amyloid fibrils which play an important role in PD. We screened 5j and 5k against *in vitro* generated αS pre-formed fibrils (PFFs) by fluorescence spectroscopy. Notably, probes 5j and 5k demonstrated a significant turn-on of orange fluorescence in the presence of PFFs, exhibiting emission maxima at 575 nm and 580 nm, respectively (Fig. 3a and b). Probes 5b–f and 5h didn't show “turn on” fluorescence, as expected, due to lack of environmental effects. Analysis of the tabulated photophysical properties of 5j and 5k in the presence and absence of αS fibrils revealed an increase in both *ε* (3-fold for 5j and 2.2-fold for 5k) and QY (122-fold for 5j and 218-fold for 5k). These enhancements contributed to a turn-on effect with 364 and 476-fold increases in brightness for 5j and 5k, respectively (Table 3). Furthermore, these dyes exhibited large Stokes shifts in the presence of PFFs: 140 nm and 117 nm for 5j and 5k, respectively, making them suitable for imaging applications with heightened sensitivity by minimizing background fluorescence at the excitation wavelength (Table 3).

**Table 3:** Spectral properties of 5j and 5k in the absence and presence of αS fibrils

Dye	PFFs	*λ*_abs_ a (nm)	*λ*_ex_ b (nm)	*λ*_em_ c (nm)	*ε* d (M^−1^ cm^−1^)	Stokes shift^e^ (nm)	Fluorescence QY^f^ (%)	Relative brightness^g^	Fluorescence lifetime^f^ (ns)
5j	−	372	—	—	15 385	—	0.17		—
	+	359	435	575	45 956	140	20.7	364	1.90
5k	−	393	—	—	20 071	—	0.15		—
	+	386	463	580	43 864	117	32.7	476	2.27

a Maximum absorption wavelength (*λ*_abs_) in the presence of αS PFFs corresponds to a mixture of bound and unbound dye.

b Maximum excitation wavelength (*λ*_ex_) better represents the bound form of dye.

c Maximum emission wavelength (*λ*_em_) corresponds to a bound dye.

d Molar absorptivity (*ε*) was measured at *λ*_abs_.

e Stokes shift was determined as the difference between *λ*_ex_ and *λ*_em_. Final dye and PFF concentrations were 10 and 50 μM, respectively, for measurements of *λ*_abs_, *λ*_ex_, *λ*_em_, and Stokes shift.

f Fluorescence quantum yield (QY) and lifetime measurements were made with 100 μM dye and PFFs.

g Relative brightness was determined as the ratio of ε QY in the presence and absence of PFFs. *λ*_ex_/*λ*_em_ = 435/575 nm for 5j and 463/580 nm for 5k.

Additionally, the observed *λ*_em_ values when bound to PFFs are consistent with an ACN-like environment. However, in comparing ACN and ACN/PBS results (Tables 2 and S12 in the ESI†), these enhancements in ACN were accompanied by decreases in *ε* and were smaller than those observed for PFF-bound forms of the compounds (Tables 3 and S12 ESI†). Thus, we conclude that turn on results from contributions involving both solvatochromism and restriction of free rotation. Additionally, the probe's binding to αS fibrils was characterized through fluorescence lifetime measurements. As depicted in Fig. 3c and d, the probes exhibited fluorescence lifetimes (*τ*) of 1.90 ns for 5j and 2.27 ns for 5k when bound to PFFs, similar to the lifetime of the key precursor 3 (2.32 ns) (Tables 3, S13 and S15 in ESI†). This suggests that the PFF-bound forms represent unquenched bimane fluorescence.

For 5j, emission peaks at 450 and 575 nm were observed, likely corresponding twisted and planar states of the molecule, respectively, where the twisted state has emission arising from the bimane core (∼464 nm). Such a double peak would complicate interpretation of fibril binding data for 5j. Together with the larger change in brightness upon fibril binding, these photophysical properties imply that probe 5k is a superior probe for αS fibrils compared to 5j. We therefore evaluated the binding affinity of 5k through fluorometric titration in the presence of 100 μM αS PFFs. The total intensity was plotted against the probe concentration and fit to a *K*_d_ of 1.45 ± 0.43 μM (Fig. 3f), a reasonably high affinity meriting its further investigation as an αS imaging probe.

### Probe selectivity for αS fibrils

**Fig. 4:**
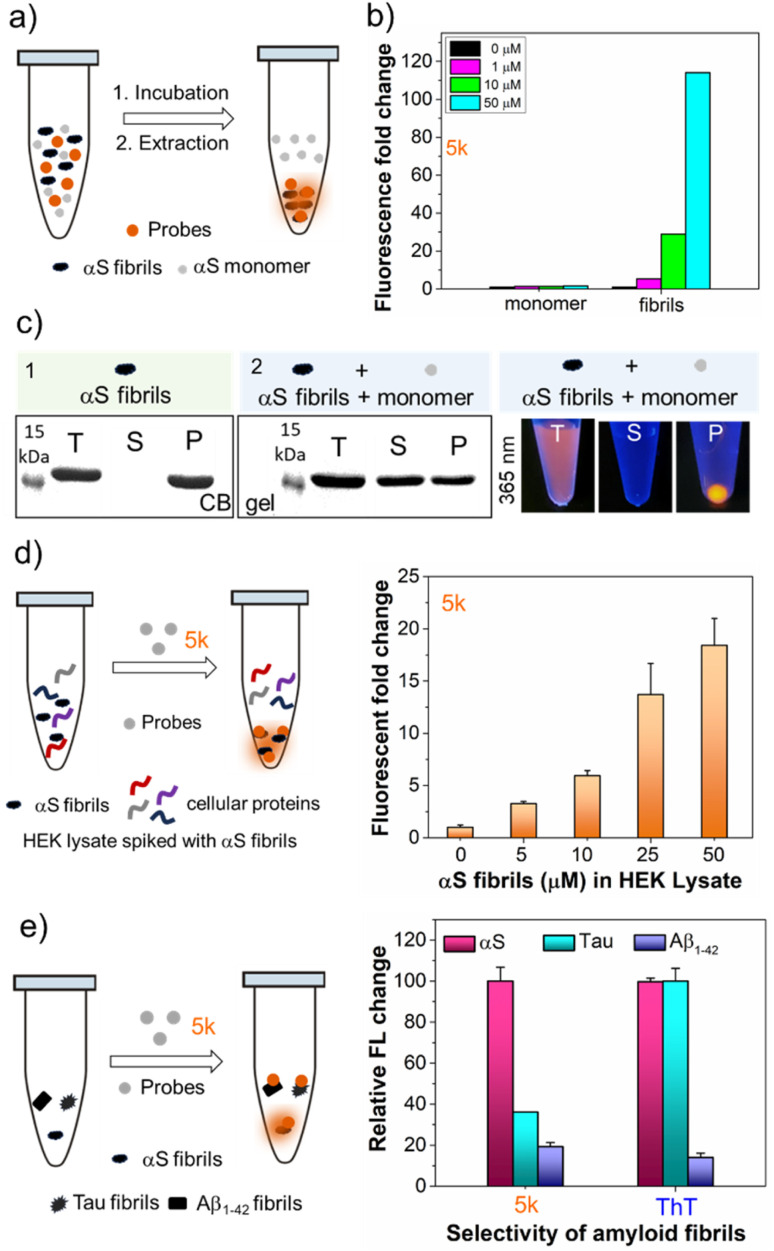
Selectivity of 5k for αS fibrils. (a) Schematic representation of selective detection of αS fibrils over monomer. (b) Dose-dependent fluorescence “turn-on” with αS fibrils and monomer. (c) Fractionation of 5k ((1) control with only fibrils (2) fibrils spiked with monomer) confirms the selective binding to αS fibrils over αS monomer revealed by SDS-PAGE and fluorescence of T, S, P fractions under handheld UV light (365 nm); T: total; S: supernatant; P: pellet. (d) Left: schematic representation of selective detection of αS fibrils in the presence of cellular proteins that are abundant in the cytosolic HEK cell lysate; right: fluorescence fold change of 5k (1 μM) with αS fibrils (0–50 μM) at 10 mg mL^−1^ HEK lysate concentration. (e) Amyloid selectivity of the probe 5k (1 μM) compared to the standard amyloid binding dye ThT with different amyloid fibrils of αS, tau and Aβ_1–42_ that are present in patients with neurodegenerative diseases. *λ*_ex_/*λ*_em_ = 463/580 nm for 5k. Error bars represent SD of 3 measurements.

To be useful for detecting αS fibrils in the study of PD and AD, a probe must detect αS amyloid forms while minimizing non-specific binding in complex biological environments. The dose-dependent increase in the fluorescence intensity of 5k occurs only with αS fibrils, not monomers (Fig. 4b). Interestingly, upon binding of 5k with αS fibrils, immediate precipitation occurred, enabling the separation and isolation of fibrils not only from buffer solutions but also from unincorporated monomer through simple centrifugation. This is confirmed by SDS-PAGE gel analysis, suggesting that probe 5k selectively binds to the fibrils and could be used to isolate fibrils (Fig. 4c). In 2022, Rodrigues *et al.* introduced a technique known as amyloid precipitation to isolate protein aggregates.^26^ This method involves an amyloid-binding ligand attached to a biotin moiety, facilitating surface immobilization through streptavidin-coated magnetic beads. We anticipate that 5k could be used in a similar fashion.

To assess the probe's potential for imaging in biological samples, we measured the ability of 5k to detect varying PFF concentrations in the presence of cytosolic human embryonic kidney (HEK) cell lysate (10 mg mL^−1^ total protein). We observed similar results to the binding studies in buffer, showing the ability to detect protein in the low μM range (Fig. 4d). Since the low backgrounds in cell lysates indicated minimal off-target binding to soluble proteins, we wished to determine whether our probe was selective towards αS PFFs *versus* fibrils of tau and the 42 amino acid amyloid-β variant (Aβ_1–42_), which are typically observed in AD patient brains.^27–29^ The ability to distinguish αS from tau and Aβ_1–42_ is important to understanding the overlapping pathology of AD, PD, and other related neurodegenerative diseases.^30,31^ We mixed 5k with αS, tau, and Aβ_1–42_ fibrils and recorded the fluorescence intensity. As one can see from Fig. 4e, at 10 μM fibril concentrations, 5k fluorescence turn-on when binding to αS is 2.8-fold higher than tau and 5.2-fold higher than Aβ_1–42_. The emission maximum is blue shifted for 5k bound to αS (584 nm) *vs.* tau (596 nm), implying that the dye is in a different environment (ESI, Fig. S21†). So, although 5k binds to both αS and tau, it has a greater fluorescence activation based on the specific mechanism of binding to αS. This is in contrast to ThT, which has high turn-on with both αS and tau (Fig. 4e). Thus, our probe shows much more conformational selectivity than ThT.

Next, we were interested in studying whether the dual sensitive nature of our probes can differentiate αS fibrils from the surrounding environment with different polarity and viscosity. To this end, we investigated the exposure of monomeric αS to varying concentrations of trimethylamine *N*-oxide (TMAO), a naturally occurring osmolyte abundant in aquatic organisms.^32–34^ Previous studies have demonstrated that αS undergoes successive compaction and forms soluble oligomers with increasing amounts of TMAO.^35–38^ Fluorescence measurements with probe 5k showed a strong turn-on of fluorescence in the presence of TMAO and αS, with no significant turn-on in solutions of TMAO alone. For TMAO/αS solutions, we observed distinct *λ*_em_ values around 567 nm, 566 nm, and 560 nm for 2 M, 4 M, and 6 M TMAO, respectively (Fig. S23 in ESI†). These observed changes in 5k fluorescence correspond to changes in binding sites with the compactness or oligomeric nature of αS, where the turn-on results primarily from changes in viscosity and the blue-shifting results from a less polar environment for oligomeric αS. This trend is consistent with fluorescence measurement of 5k involving differing solvents and glycerol/ethylene glycol mixtures (Fig. 2). The *λ*_em_ value of 560 nm for αS in 6 M TMAO is similar to the blue-shifted emission (*λ*_em_ = 555 nm) observed when binding to BSA (Fig. S24 in ESI†). In contrast, probe 5k showed *λ*_em_ of 580 nm with αS fibrils (Fig. 3b and Table 3). Together, these results suggest that the 5k probe is capable of distinguishing αS fibrils from other aggregates through its sensitivity to the polarity and viscosity of its local environment.

### Detecting αS fibrils in clinical samples

**Fig. 5:**
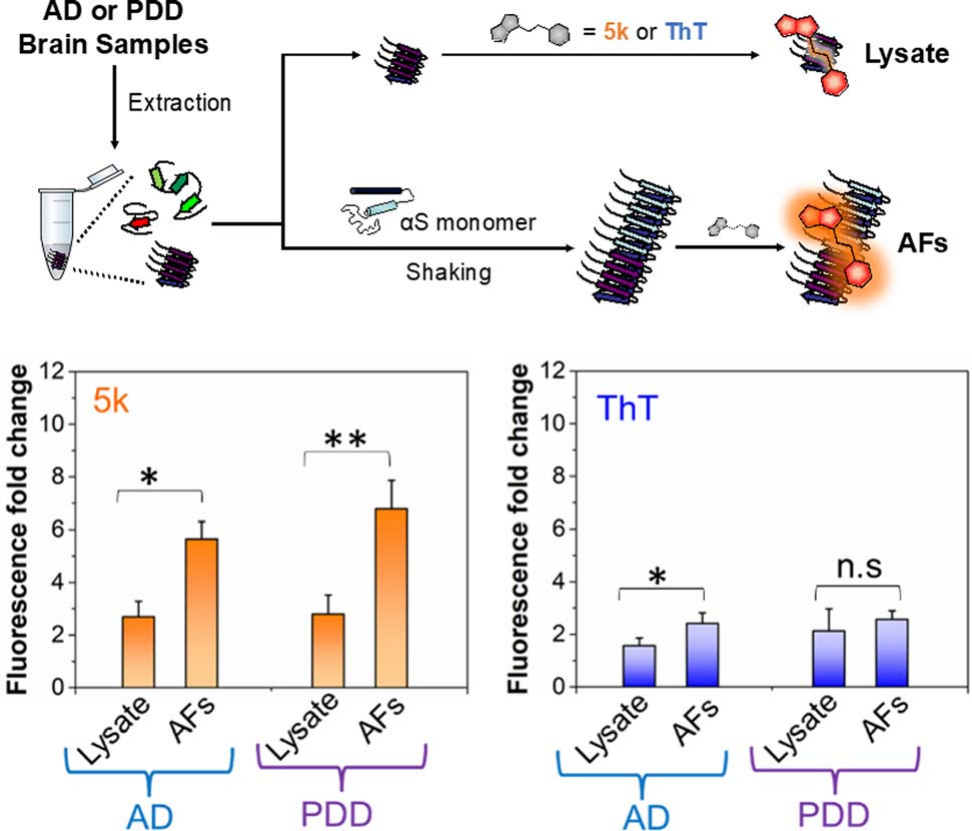
Selective detection of αS fibrils amplified from PDD lysates over AD. Above: workflow for detecting amplified fibrils (AFs) generated from seeds extracted from brain samples of AD or PDD patients, as well as control samples containing only lysate. Bottom Fluorescence fold change relative to dye alone for 5k or ThT incubated with lysate or AF samples. The fluorescence bar graph represents the averaged data based on samples from three different AD or PDD patients. The final lysate or AFs and probe concentrations were 1 μM. The fold change in fluorescence fold was determined relative to the fluorescence of the free probe in PBS buffer. Fluorescence measurements were performed on a Tecan plate reader with the following excitation and emission wavelengths. *λ*_ex_/*λ*_em_ = 463/580 nm for 5k and 450/482 nm for ThT. Error bars represent the SD of 3 measurements. Statistical analysis: *p < 0.05 and **p < 0.005.

Given the selectivity of 5k for αS fibrils over monomer and fibrils of tau and Aβ_1–42_, as well as its low background fluorescence in cell lysate, we wished to determine whether the probe could be used to detect of αS fibrils in clinical samples. There has been great recent excitement about a fibril amplification assay for use as a PD biomarker.^39,40^ However, this enzyme-linked assay is somewhat operationally complex and there are questions about its response to different αS fibril conformations, or “strains.” In recent years, it has become clear from structural and biochemical studies that different αS fibril polymorphs are present in different diseases, and that these also differ from those formed *in vitro*.^7,41–43^ We have recently shown that fibril strains from Lewy bodies in PD and related synucleinopathies such as PDD can be faithfully amplified *in vitro* using seeds derived from patient material and recombinant αS monomer.^44^ However, monitoring these reactions has been challenging because ThT does not effectively detect these amplified fibrils (AFs), as one can see in Fig. 5. AD often features significant αS co-pathology with tau, leading to background fluorescence from ThT that should be less of an issue for 5k given its greater selectivity for αS. Therefore, we thought that an assay using 5k could have significant value if it were selective, as implied by our *in vitro* PFF studies.

To determine whether our fluorescent probe could detect AFs, and potentially distinguish between fibrils strains from different sources, we prepared lysates from three AD cases and three PDD cases. For a portion of each lysate, fibrillar material was amplified with αS monomer (see ESI, pages S48–S51† for sample preparation and characterization of lysates and AFs). We then compared the fluorescence of 5k in the presence of the AFs, or in the lysates alone (Fig. 5). We found that while there was some fluorescence from endogenous fibrillar material in the brain lysate samples, there was a clear increase in fluorescence upon fibril amplification. Interestingly, probe 5k showed higher sensitivity to PDD derived AFs (significance of *p* < 0.005) than AFs of AD (significance of *p* < 0.05). In contrast, ThT is unable to differentiate the lysates and AFs of PDD samples and barely registers significance for detection of AFs in AD samples. While further optimization is necessary, these data show that 5k has the potential to be used in a clinical biomarker assay that is specific for αS in spite of abundant co-pathology of tau and amyloid-β in AD samples.

## Conclusions

In summary, we have demonstrated advances in design and synthesis of the underexplored bimane fluorescent scaffold (see ESI Fig. S27† for analysis of bimane literature relative to similar fluorophores) enabling a rational approach to creating novel styryl bimane dyes. We synthesized bimane scaffold 1 on a multi-gram scale with improved yields compared to those previously reported. Key precursor 3 can be utilized to access styryl bimane analogues through the H–W–E reaction with diverse aryl aldehydes. Detailed investigations of a set of styryl bimane probes with varied electronic properties showed tunable absorption and emission in the visible region with large (>200 nm) Stokes shifts. With this library, we gained insights into the structure–photophysical relationship of *para*-substituted derivatives by Hammett and DFT analysis to design future bimane probes with predictable photophysical properties. Interestingly, 5j and 5k with electron-donating groups show rotor effects with sensitivity to both polarity and viscosity through ICT and TICT mechanisms, respectively. The characteristics of 5j and 5k enable their application as turn-on fluorescent probes for detecting fibrillar aggregates of the αS protein that are a hallmark of PD. In particular, probe 5k demonstrated selective binding to αS PFFs in three key aspects: (1) over monomers and enabling a method to isolate αS fibrils, (2) in cell lysates with minimal off-target binding, and (3) over other protein fibrils such as tau and amyloid-β. Moreover, probe 5k has the ability to differentiate the αS fibrils from αS oligomers and other protein aggregates with environments of different polarity and viscosity through distinct emission wavelengths. Excitingly, it shows the ability to detect αS AFs from PDD with higher sensitivity than AD, so it has potential to be used in a clinical diagnostic with a simple operational workflow. Efforts to improve affinity, selectivity, and turn-on through derivatives of 5k optimized for this application are underway, where modifications of the methyl positions around the bimane core as well as changes to the aryl moiety will be used to tune protein binding and photophysical properties.

In addition to translation of styryl bimanes as αS binding probes, this initial report opens avenues to several other applications in biological systems, membrane chemistry, and materials science. For example, we may be able to develop the probes for tissue imaging by extending conjugation and/or augmenting the D–π–A system to make small near IR probes. We can also use information gained in fluorescent imaging studies to guide the development of positron emission tomography (PET) probes for *in vivo* imaging of PD.^45^ Probes featuring dual sensitivity through rotor effects can provide insight into the polarity and viscosity of cellular components or microenvironments in biological tissues. In another example, the restriction of rotation that leads to fluorescence turn-on when binding amyloids could also be exploited in the solid state. Indeed, preliminary characterization of amorphous forms of styryl bimanes indicates that they have potential for application in organic materials (Fig. 1d and ESI, Fig. S28†). Very recently, Grynszpan and co-workers reported thioxobimanes that significantly modulate the absorption maxima in the visible region, showing promise as ligands for transition metals and for developing turn-on fluorescent chemosensors.^46^ Similar to our previous studies of dimethylaminoquinolines,^47,48^ we find that there is much to be gained by exploring an underappreciated fluorescence scaffold, and that from the derivatives made here, one can rapidly use information gained on the photophysical mechanisms to rationally design new probes with valuable properties for biological and clinical applications.

## Data availability

General information, detailed experimental procedures, characterization of probes by ^1^H, ^13^C NMR, MS and X-ray crystallography, photophysical properties by UV-Vis absorption and fluorescence spectroscopy, computational studies and Cartesian coordinates of the optimized geometries for probes at #APFD/6-311+G (2d, p) level, protocol for protein expression, purification and characterization by MALDI-MS, characterization of patient tissue and amplified fibril preparation, and procedures for the binding studies are available in the ESI.† Original data will be made available upon email request to the corresponding author.

## Author contributions

Y. V. and E. J. P. designed the overall research plan. Y. V. performed all synthesis, photophysical experiments and protein purification. N. M. provided fibrils amplified from patient samples. The manuscript was written with input from all authors. All authors have given approval to the final version of the manuscript.

## Conflicts of interest

There are no conflicts to declare.

## Supplementary Material

SC-015-D4SC00024B-s001

SC-015-D4SC00024B-s002
