# Supplementary material for: Highly tunable bimane-based fluorescent probes: design, synthesis, and application as a selective amyloid binding dye
Source: Chem Sci. 2024 Mar 26;15(16):6053–63. doi: 10.1039/d4sc00024b (PMC11040648; doi:10.1039/d4sc00024b)
Supplement: SC-015-D4SC00024B-s001 [file SC-015-D4SC00024B-s001.pdf]

Electronic Supplementary Material (ESI) for Chemical Science.
This journal is © The Royal Society of Chemistry 2024

Supporting Information

Highly Tunable Bimane-Based Fluorescent Probes: Design, Synthesis, and Application as Selective Amyloid Binding Dye

Yarra Venkatesh,[a] Nicholas P. Marotta,[c] Virginia M.-Y. Lee,[c] and E. James Petersson[a,b]*

[a]Department of Chemistry, University of Pennsylvania, 231 South 34th Street, Philadelphia, Pennsylvania 19104, USA.

[b]Biochemistry and Molecular Biophysics Graduate Group, Perelman School of Medicine, University of Pennsylvania, Philadelphia, Pennsylvania 19104, USA.

[c]Department of Pathology and Laboratory Medicine, Center for Neurodegenerative Disease Research, University of Pennsylvania, 3600 Spruce Street, Philadelphia, Pennsylvania 19104, USA.

*E-mail: ejpetersson@sas.upenn.edu

Table of Contents

General Information S3

Multi-Gram Scale Synthesis of Bimane Core S4-S5

Synthesis and Characterization of key precursor **3** S5-6

General Procedure for H-W-E reaction.. S7

Reaction Optimization for H-W-E reaction.. S8-S9

Synthesis and Characterization of **5a-5k** S10-S13

Crystal Data and Structure Refinement S14-S24

General Photophysical Characterization Methods S25-S26

Photophysical Properties of **3** and **5a-5k** in ACN S27-S28

Solvatochromism of **3, 5b, 5d, 5j**.. S29

Fluorescent Quantum Yield Measurements of **5a-5k** S30-S32

Effect of pH on **5h** and **5j** S33

Fluorescence Lifetime Measurements of **3**, **5c-5f** …………………….………………… S34-S35

Computational Studies ……………………………….…………………………….. S36-S40

Production of α-Synuclein and 1N4R Tau …….…………………..…………….. S41-S44

Fibril Preparation ……………………………......…………………………….. S45-S47

Fibril Binding Characterization ………………......………......………………...………….. S48

Absorbance and Fluorescence Measurements with αS Fibrils …………......…………... S49-S50

Fluorescent QY and lifetime measurements of **5j** and **5k** with αS fibrils………………. S51-S52

Excitation and Fluorescence Measurements of **5j**, **5k** and **ThT** with Tau Fibrils ……..….... S53

Excitation and Fluorescence Measurements of **5j**, **5k** and **ThT** with $A\beta_{1-42}$ Fibrils ……..…... S54

Experimental Procedure for HEK cell lysate preparation and Selectivity of **5k**………………..S55

Experimental procedure for the fluorescent measurements of **5k** with TMAO……………..…S56

Patient Tissue Characterization and Amplified Fibril Preparation……….....…..………….. S57-S60

Fluorescence Measurements with Patient Tissue Lysate Samples ……......…...……………….. S61

Analysis of Bimane Literature ………...…………………………………..……………….. S62

Photographs of **1** and **5a-5k** Solid State Emission ………..……………………………… S62

NMR spectra ……………………………………………………….………………….. S63-S73

Optimized Ground and Excited State Geometries ……………………………………... S74-S82

References ………………………………………………...………………...……..….. S83

General Information

Materials. Trichloroisocyanuric acid (TCCA), trimethyl phosphite and all other derivatives of aryl aldehydes were purchased from Millipore Sigma (St. Louis, MO, USA). Flash column chromatography was performed using Silicycle silica gel (40-63 μm (230- 400 mesh), 60 Å irregular pore diameter). Thin-layer chromatography (TLC) was performed on TLC Silica gel 60G F254 plates from Millipore Sigma. Reagents were purchased at the highest commercial quality and used without further purification, unless otherwise stated. The β-amyloid (1-42) peptide, human (Cat. No. RP10017) was purchased from Genscript (Piscataway, NJ).

Instruments. Nuclear magnetic resonance (NMR) spectra were obtained on a Bruker UNI-400 MHz or UNI-600 MHz instrument (Billerica, MA, USA) and are calibrated using peaks from residual protic solvent in deuterated solvent. The following abbreviations were used to denote multiplicities: s = singlet, d = doublet, t = triplet, q = quartet, m = multiplet, br = broad). Low-resolution mass spectra (LRMS) were obtained on a Waters Acquity Ultra Performance LC connected to a single quadrupole detector mass spectrometer (Waters Corp.; Milford, MA, USA). High-resolution mass spectra (HRMS) were obtained by Dr. Charles Ross III at the University of Pennsylvania's Mass Spectrometry Center on a High-resolution electrospray ionization mass spectra (ESI-HRMS) using Waters LCT Premier XE liquid chromatograph/mass spectrometer. X-ray diffraction data obtained on a Rigaku XtaLAB Synergy-S diffractometer equipped with an HPC area detector (HyPix-6000HE) and employing confocal multilayer optic-monochromated Mo-Kα radiation (λ=0.71073 Å) or Cu=Kα radiation (λ=1.54184 Å) at a temperature of 100K and solved by Dr. Patrick Carroll at the University of Pennsylvania. Absorbance readings for the DC assay were made on a Tecan M1000 plate reader (Mannedorf, Switzerland). UV-Vis absorption spectra were acquired on a Thermo Scientific Genesys 150 UV-Vis spectrometer (Waltham, MA, USA) using quartz cells with a 1 cm cell path length (Starna Cells, Inc 120ul UV cells). Fluorescence spectra were acquired on a Tecan M1000 plate reader. Quantum yield (QY) measurements were performed using a Jasco FP-8300 Fluorimeter with ILF-835 integrating sphere attachment (Easton, MD, USA). Fluorescence lifetime measurements were made using a Photon Technology International (PTI) QuantaMaster™ 40 fluorescence spectrometer (Birmingham, NJ, USA).

Multi-Gram Scale Synthesis of Bimane Core

Synthesis of 3,4-dimethyl-2-pyrazolin-5-one. Ethyl 2-methylacetoacetate (30 g, 208.3 mmol, 1 equiv) was condensed with 4 equiv. of hydrazine (26.7 g, 833.2 mmol, 35 wt. % in H_2O) under sonication for 30 min to give a white precipitate. After filtration of the white precipitate and washing several times with ethyl acetate followed by dichloromethane to get rid of the unreacted starting materials, the precipitate was dried overnight to afford 22.485 g of compound 3,4-dimethyl-2-pyrazolin-5-one in 96.4% isolated yield. ^{1}H NMR and LRMS characterization data matched previous reports.[1]

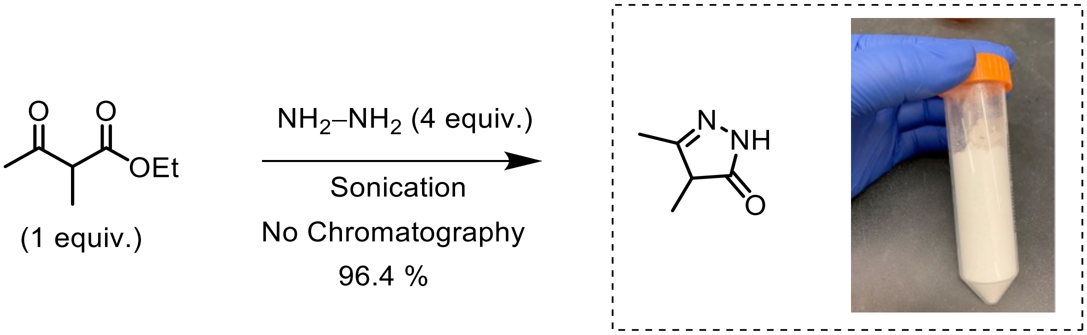

Synthesis of 3,4-dimethyl-4-chloro-2-pyrazolin-5-one. 3,4-dimethyl-2-pyrazolin-5-one (20 g, 178.4 mmol) was dissolved in 200 mL of dichloromethane (DCM). The solution was cooled on an ice bath. Trichloroisocyanuric acid (TCCA, 13.7 g, 59.5 mmol) was slowly added to the reaction mixture over a period of 30 min. After completion of the addition, the reaction mixture was stirred overnight at room temperature and the cyanuric acid byproduct was filtered off. The filtrate was concentrated under reduced pressure at 40 °C to afford 22.05 g of 3,4-dimethyl-4-chloro-2-pyrazolin-5-one in 84 % isolated yield. ^{1}H NMR and LRMS characterization data matched previous reports.[1,2]

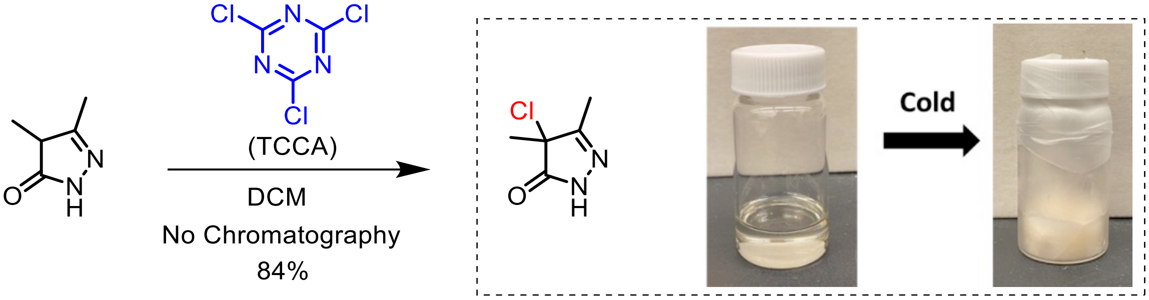

Synthesis of 2,3,5,6-tetramethyl-1H,7H-pyrazolo[1,2-a]pyrazole-1,7-dione (1). 3,4-Dimethyl-4-chloro-2-pyrazolin-5-one (15 g, 102.34 mmol, 1 equiv) was dissolved in 150 mL DCM and cooled on an ice bath. Then add potassium carbonate hydrate (K_2CO_3.1.5 H_2O) (59.32 g, 429.83 mmol, 4.2 equiv) and stir for 18 hours at room temperature. Then the K_2CO_3 was filtered, and the filtrate concentrated to afford a mixture of *cis* (**1**) and *trans* (**1'**) isomers of bimane. 2,3,5,6-tetramethyl-1*H*,7*H*-pyrazolo[1,2-a]pyrazole-1,7-dione, with **1** as the major product. The reaction mixture was purified by column chromatography eluting with 40–70% ethyl acetate in hexane to afford the 71% of compound **1** (**R_f = 0.1, 70% EtOAc in hexane) and compound **1'** (R_f = 0.64, 70% EtOAc in hexane) in 18% yield. ^{1}H NMR and LRMS characterization data matched previous reports.[1]

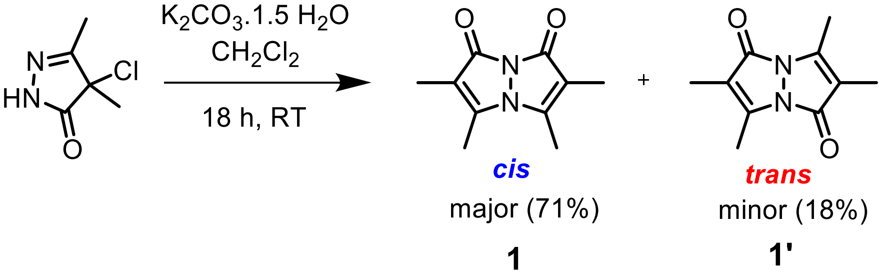

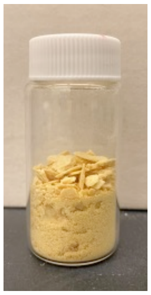

Synthesis and Characterization of 3

Synthesis of 3-(bromomethyl)-2,5,6-trimethyl-1*H*,7*H*-pyrazolo[1,2-a]pyrazole-1,7-dione (2). Compound **1** (3 g, 15.61 mmol) was dissolved in dry 120 mL of DCM. The solution was cooled on an ice bath and a bromine solution (0.8 mL, 15.61 mmol) in 30 mL of DCM solvent was added dropwise over a period of 30 min under cold conditions. Upon completion of the addition, the reaction mixture was stirred for another 30 min under cold conditions. The progress of the reaction was monitored by TLC. Upon completion of the reaction, the resulting mixture was neutralized with cold water and then extracted with DCM. The organic layer was collected and concentrated by rotary evaporation. The crude product was purified by flash column chromatography eluting with 30–60% ethyl acetate in hexane to afford the desired monobrominated product **2** (**R_f = 0.17, 70% EtOAc in hexane**)** in 74% yield and we also isolated the dibrominated product **2'** (R_f = 0.45, 70% EtOAc in hexane) in 21% yield. ^{1}H and ^{13}C NMR and LRMS characterization data matched previous reports.[1]

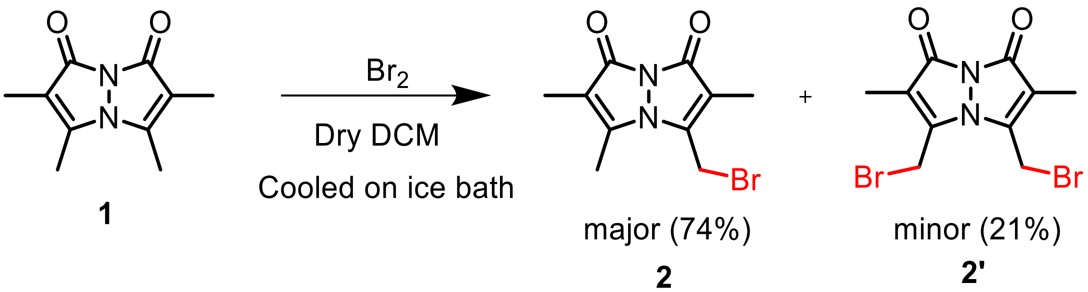

Synthesis of dimethyl ((2,5,6-trimethyl-1,7-dioxo-1*H*,7*H*-pyrazolo[1,2-a]pyrazol-3-yl)methyl)phosphonate (3). A mixture of monobromobimane (**2**, 2.0 g, 7.38 mmol) and trimethyl phosphite (2.85 mL) was heated at 115 °C for 30 min until it became homogeneous and then solidified. After trituration with hexane (50 mL), the mixture was slurried in ethyl acetate and filtered to remove the remaining trimethyl phosphite. Then, the solid product was redissolved in DCM and purified by flash column chromatography using 3% methanol in DCM as an eluent to afford 1.82 g of the phosphonate **3** (R_f = 0.37, 3% methanol in DCM) as a light-yellow solid in 82% isolated yield.

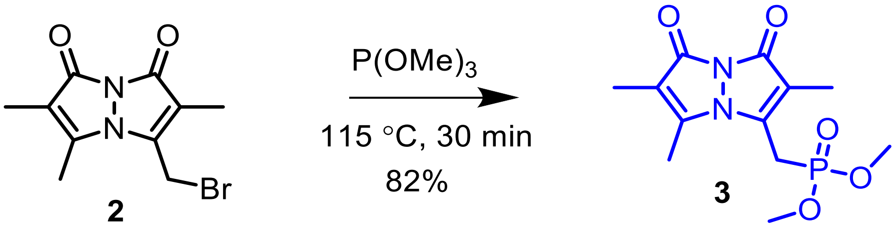

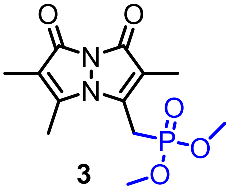

^{1}H NMR (400 MHz, CDCl$_3$) δ 3.78 (d, *J* = 11.1 Hz, 6H), 3.20 (d, *J* = 22.0 Hz, 2H), 2.42 (s, 3H), 1.84 (d, *J* = 4.1 Hz, 3H), 1.80 (s, 3H). ^{13}C NMR (101 MHz, CDCl$_3$) δ 160.7, 160.2, 146.5, 140.0, 114.7, 113.2, 53.7, 25.2, 23.8, 11.9, 7.0. HRMS (ESI$^+$) calcd for $C_{12}H_{17}N_2O_5P$ $[M + H]^+$, 301.0953; found: 301.0965.

Horner-Wadsworth-Emmons (H-W-E) reactions were performed with one of two general procedures:

H-W-E General procedure A. To a solution of dimethyl bimanephosphonate **3** (1 mmol) in acetonitrile (ACN)/H_2O (2 mL, 6:4 v/v), add the aryl aldehydes **4a-c**, **4g**, and **4i** (1 mmol) and K_2CO_3 (1.5 mmol) and heat at 90 ˚C for 30 min-2 h. The reaction progress was checked by TLC, upon completion of the reaction, the solution was cooled to room temperature and the organic layer was extracted with DCM, washed with water (2 x 50 mL), dried using Na_2SO_4, and concentrated under vacuum to yield the crude mixture. Then, the product was purified by recrystallization from MeOH.

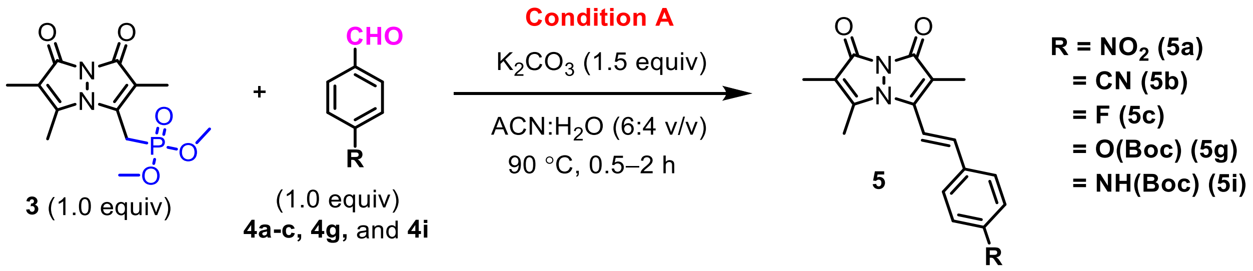

H-W-E General procedure B. To a solution of phosphonate **3** (1 mmol) in dry DMF, the aryl aldehydes **4d-f** and **4k** (1 mmol) and NaOMe (1.5 mmol) were added, and the resulting solution was stirred at RT for 40 h under argon atmosphere. The reaction progress was monitored by TLC, and upon completion, the organic layer was extracted with DCM, washed with water (2 x 50 mL), dried using Na_2SO_4, and concentrated under vacuum to yield the crude product. Then, the product was purified by flash column chromatography using 3% methanol in DCM as an eluent.

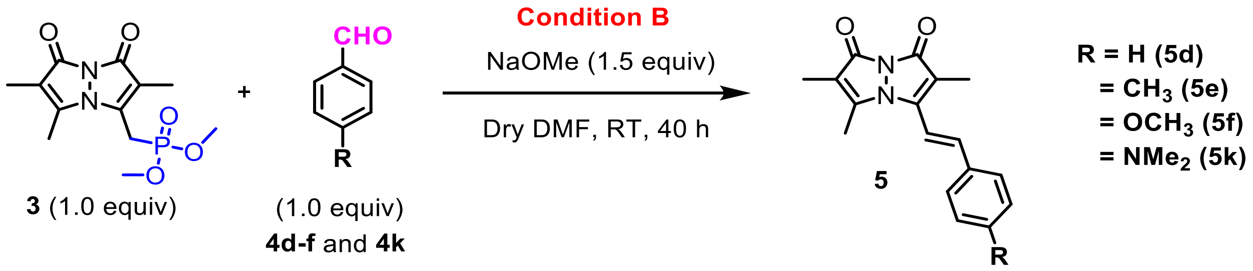

Scheme S1. Optimization of H-W-E reaction of **3** and 4-(dimethylamino)benzaldehyde[a]

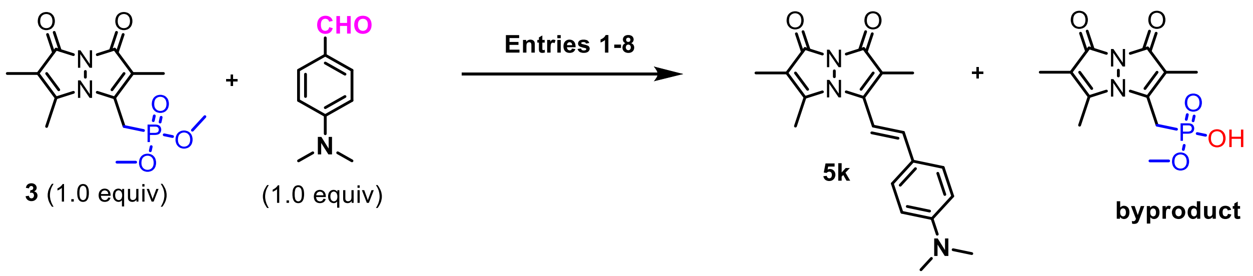

Entry	Solvent	Base	Temp	Time (hours)	Yield (%)
1	ACN/H_2O	K_2CO_3	RT	40 h	2.3
2	ACN/H_2O	K_2CO_3	90 ˚C	2 h	9
3	DMF	KO*t*Bu	RT	40 h	27
4	DMF	NaOMe	RT	40 h	46
5	DMF	NaOMe	60 ˚C	40 h	38.4
6	DMSO	KO*t*Bu	RT	40 h	3.5
7	DMSO	NaOMe	RT	40 h	31
8	DMSO	NaOMe	60 ˚C	40 h	11.5

[a]Reaction conditions: **3** (1 equiv), aryl aldehyde **4k** (1 equiv), KO*t*Bu (1.5 equiv) or NaOMe (1.5 equiv), K_2CO_3 (1.5 equiv).

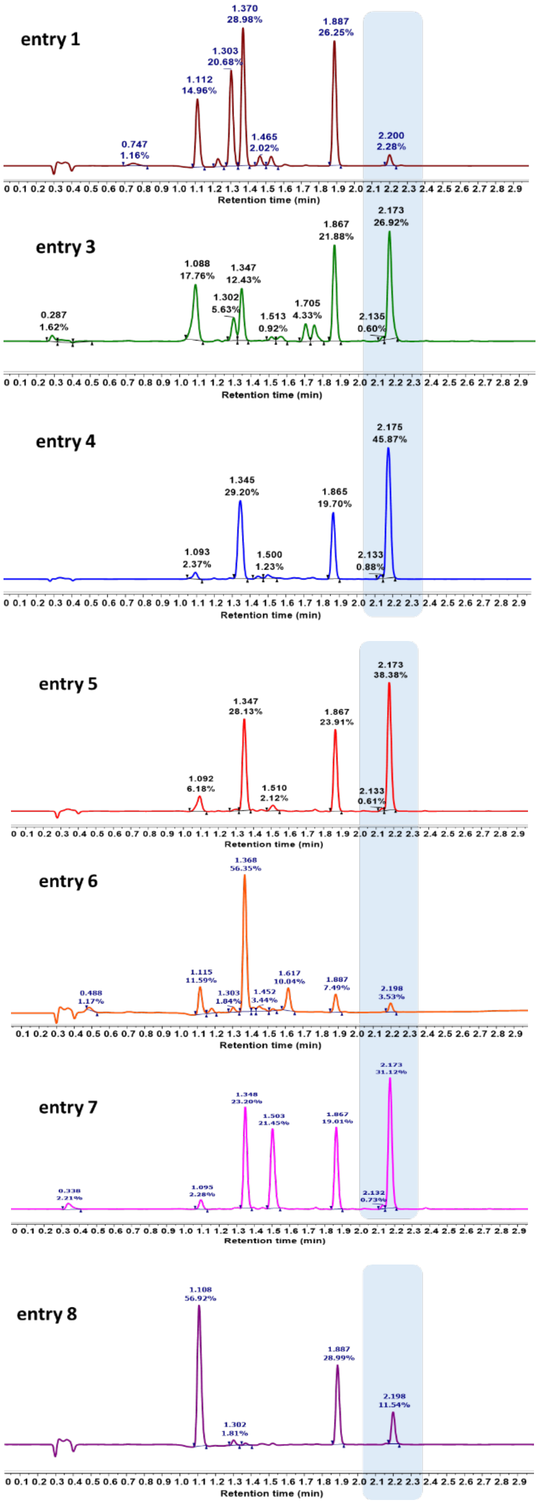

Fig. S1. Liquid Chromatography (LC) chromatogram for the conversion of **5k** under different optimized reaction conditions.

Synthesis and Characterization of 5a-5k

(*E*)-2,3,6-trimethyl-5-(4-nitrostyryl)-1*H*,7*H*-pyrazolo[1,2-a]pyrazole-1,7-dione (5a).

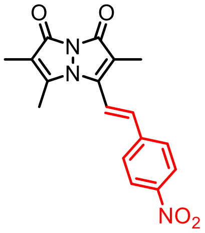

Following General Procedure A with 4-nitrobenzaldehyde (**4a**, 50.3 mg, 0.33 mmol, 1 equiv, purchased from Aldrich) at 90 °C for 30 min afforded 106 mg (98 % isolated yield, yellow solid) of **5a**. ^{1}H NMR (400 MHz, $CDCl_3$) δ 8.29 (d, J = 8.9 Hz, 2H), 7.70 (d, J = 8.8 Hz, 2H), 7.17 (d, J = 16.5 Hz, 1H), 6.96 (d, J = 17.3 Hz, 1H), 2.30 (s, 3H), 2.03 (s, 3H), 1.84 (s, 3H). ^{13}C NMR (101 MHz, $CDCl_3$) δ 161.7, 161.3, 148.5, 146.4, 141.0, 138.5, 128.1, 124.7, 118.3, 115.2, 114.3, 12.7, 8.6, 7.2. HRMS (ESI$^+$) calcd for $C_{17}H_{15}N_3O_4$ $[M + H]^+$, 326.1141 ; found: 326.1156.

(*E*)-4-(2-(2,5,6-trimethyl-1,7-dioxo-1H,7H-pyrazolo[1,2-a]pyrazol-3-yl)vinyl)benzonitrile (5b).

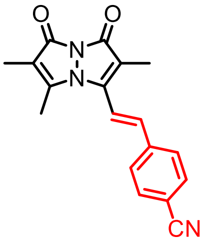

Following General Procedure A with 4-cyanobenzaldehyde (**4b**, 43.6 mg, 0.33 mmol, 1 equiv, purchased from Aldrich) at 90 °C for 1 h afforded 85.3 mg (83.6 % isolated yield, yellow solid) of **5b**. ^{1}H NMR (400 MHz, $CDCl_3$) δ 7.73 (d, *J* = 8.4 Hz, 2H), 7.63 (d, *J* = 8.5 Hz, 2H), 7.12 (d, *J* = 16.5 Hz, 1H), 6.91 (d, *J* = 16.5 Hz, 1H), 2.28 (s, 3H), 2.02 (s, 3H), 1.84 (s, 3H). ^{13}C NMR (101 MHz, $CDCl_3$) δ 161.7, 161.3, 148.6, 146.6, 139.1, 139.0, 133.1, 127.9, 118.4, 117.6, 115.0, 114.2, 113.5, 12.7, 8.6, 7.2. HRMS (ESI$^+$) calcd for $C_{18}H_{15}N_3O_2$ $[M + H]^+$, 306.1243; found: 306.1235.

(*E*)-3-(4-fluorostyryl)-2,5,6-trimethyl-1*H*,7*H*-pyrazolo[1,2-a]pyrazole-1,7-dione (5c).

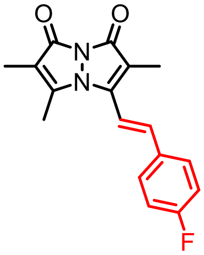

Following General Procedure A with 4-fluorobenzaldehyde (**4c**, 41.3 mg, 0.33 mmol, 1 equiv, purchased from Aldrich) at 90 °C for 2 h afforded 77.2 mg (78 % isolated yield, yellow solid) of **5c**. ^{1}H NMR (400 MHz, $CDCl_3$) δ 7.52 (dd, J = 8.9, 5.3 Hz, 2H), 7.13 (t, J = 8.6 Hz, 2H), 7.07 (d, J = 16.4 Hz, 1H), 6.71 (d, J = 16.5 Hz, 1H), 2.28 (s, 3H), 2.01 (s, 3H), 1.83 (s, 3H). ^{13}C NMR (101 MHz, $CDCl_3$) δ 164.0 (d, $^{1}J_{C-F}$ = 252.7 Hz), 161.8, 161.6, 148.6, 147.5, 140.1, 131.2, 129.4, 129.3, 116.6 (d, $^{2}J_{C-F}$ = 22.2 Hz), 113.9, 113.7, 12.7, 8.6, 7.2. HRMS (ESI$^+$) calcd for $C_{17}H_{15}FN_2O_2$ $[M+H]^+$, 299.1196; found: 299.1193.

(*E*)-2,3,6-trimethyl-5-styryl-1*H*,7*H*-pyrazolo[1,2-a]pyrazole-1,7-dione (5d): Following General Procedure B with benzaldehyde (**4d**, 35.3 mg, 0.33 mmol, 1 equiv, purchased from Aldrich) at room temperature for 40 h afforded 67 mg (72.4 % isolated yield, yellow solid) of **5d** after purification by column chromatography (3% methanol in dichloromethane, R_f = 0.46). ^{1}H NMR (400 MHz, $CDCl_3$) δ 7.53 (dd, J = 7.9, 1.9 Hz, 2H), 7.48 – 7.39 (m, 3H), 7.11 (d, J = 16.5 Hz, 1H), 6.79 (d, J = 16.4 Hz, 1H), 2.29 (s, 3H), 2.02 (s, 3H), 1.84 (s, 3H). ^{13}C NMR (101 MHz, $CDCl_3$) δ 161.9, 161.7, 148.6, 147.7, 141.4, 135.0, 130.4, 129.4, 127.5, 114.0, 113.9, 113.8, 12.7, 8.6, 7.2. HRMS (ESI+) calcd for $C_{17}H_{16}N_2O_2$ [M + H]+, 281.1290 ; found: 281.1283.

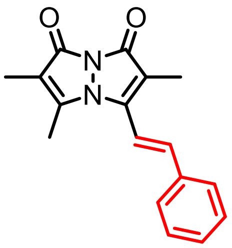

(*E*)-2,3,6-trimethyl-5-(4-methylstyryl)-1H,7H-pyrazolo[1,2-a]pyrazole-1,7-dione (5e). Following General Procedure B with *p*-tolualdehyde (**4e**, 40 mg, 0.33 mmol, 1 equiv, purchased from Aldrich) at room temperature for 40 h afforded 67 mg (68 % isolated yield, yellow solid) of **5e** after purification by column chromatography (3% methanol in dichloromethane, R_f = 0.47). ^{1}H NMR (400 MHz, $CDCl_3$) δ 7.45 (d, J = 8.1 Hz, 2H), 7.28 (d, J = 2.8 Hz, 2H), 7.10 (d, J = 16.5 Hz, 1H), 6.76 (d, J = 16.4 Hz, 1H), 2.43 (s, 3H), 2.31 (s, 3H), 2.04 (s, 3H), 1.86 (s, 3H). ^{13}C NMR (101 MHz, $CDCl_3$) δ 161.9, 161.7, 148.7, 148.0, 141.4, 140.9, 132.2, 130.1, 127.4, 113.9, 113.4, 112.9, 77.5, 77.2, 76.9, 21.7, 12.7, 8.6, 7.2. HRMS (ESI^+) calcd for $C_{18}H_{18}N_2O_2$ $[M + H]^+$, 295.1447; found: 295.1447.

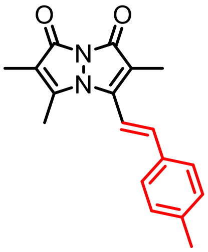

(*E*)-3-(4-methoxystyryl)-2,5,6-trimethyl-1*H*,7*H*-pyrazolo[1,2-a]pyrazole-1,7-dione (5f). Following General Procedure B with *p*-anisaldehyde (**4f**, 45.3 mg, 0.33 mmol, 1 equiv, purchased from Aldrich) at room temperature for 40 h afforded 61 mg (59 % isolated yield, yellow solid) of **5f** after purification by column chromatography (3% methanol in dichloromethane, R_f = 0.45). ^{1}H NMR (400 MHz, $CDCl_3$) δ 7.47 (d, J = 8.8 Hz, 2H), 7.05 (d, J = 16.5 Hz, 1H), 6.95 (d, J = 8.8 Hz, 2H), 6.63 (d, J = 17.1 Hz, 1H), 3.86 (s, 3H), 2.28 (s, 3H), 2.00 (s, 3H), 1.83 (s, 3H). ^{13}C NMR (101 MHz, $CDCl_3$) δ 161.7, 161.6, 161.3, 148.5, 148.0, 140.9, 128.9, 127.5, 114.6, 113.6, 112.7, 111.3, 55.5, 12.5, 8.4, 7.0. HRMS (ESI^+) calcd for $C_{18}H_{18}N_2O_3$ $[M + H]^{+,}$ 311.1396; found: 311.1408.

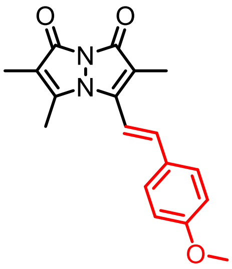

(*E*)-3-(4-hydroxystyryl)-2,5,6-trimethyl-1*H*,7*H*-pyrazolo[1,2-a]pyrazole-1,7-dione (5h).

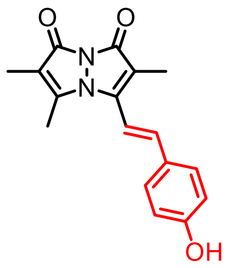

Following General Procedure A with *tert*-butyl 4-formylphenyl carbonate (**4g**, 74 mg, 0.33 mmol, 1 equiv, purchased from Aldrich) at 90 °C for 2 h afforded 100.3 mg (76 % isolated yield) of *tert*-butyloxycarbonyl (Boc) protected **5g** after purification by column chromatography (3% methanol in dichloromethane, $R_f = 0.41$).

Next, the Boc deprotection of **5g** (100 mg, 0.252 mmol, 1 equiv) was carried out under acidic conditions using trifluoroacetic acid (431.4 mg, 3.78 mmol, 15 equiv) in 2 mL DCM solvent for 6 h. The reaction progress was monitored by the TLC and upon completion, it was dried under vacuum to afford 55.3 mg of the **5h** (74% isolated yield, yellow solid, $R_f = 0.34$ in 3% methanol in dichloromethane). ^{1}H NMR (600 MHz, DMSO) δ 9.97 (s, 1H), 7.59 (d, J = 8.7 Hz, 2H), 7.20 (d, J = 16.5 Hz, 1H), 6.97 (d, J = 16.5 Hz, 1H), 6.83 (d, J = 8.7 Hz, 2H), 2.34 (s, 3H), 1.91 (s, 3H), 1.74 (s, 3H). ^{13}C NMR (151 MHz, DMSO) δ 161.1, 160.9, 159.4, 150.1, 148.8, 141.2, 129.5, 126.3, 115.8, 111.8, 110.6, 110.5, 12.1, 8.0, 6.6. HRMS (ESI$^+$) calcd for $C_{17}H_{16}N_2O_3$ $[M + H]^+$, 297.1239; found: 297.1233.

***tert*-butyl (*E*)-(4-(2-(2,5,6-trimethyl-1,7-dioxo-1*H*,7*H*-pyrazolo[1,2-a]pyrazol-3-yl)vinyl) phenyl)carbamate (5i).**

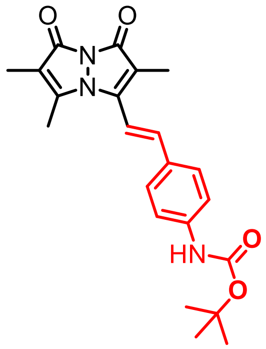

Following General Procedure A with 4-(Boc-amino)benzaldehyde (**4i**, 73.6 mg, 0.33 mmol, 1 equiv) at 90 °C for 2 h afforded 113.5 mg (86 % isolated yield, yellow solid) of **5i** after purification by column chromatography (3% methanol in dichloromethane, $R_f = 0.42$). ^{1}H NMR (600 MHz, $CDCl_3$) δ 7.45 (s, 4H), 7.04 (d, J = 16.4 Hz, 1H), 6.73 – 6.60 (m, 2H), 2.28 (s, 3H), 2.02 (s, 3H), 1.84 (s, 3H), 1.53 (s, 9H). ^{13}C NMR (151 MHz, $CDCl_3$) δ 161.9, 161.8, 152.5, 148.7, 148.0, 129.6, 128.4, 118.8, 113.9, 113.2, 112.2, 81.4, 28.5, 12.7, 8.6, 7.2. HRMS (ESI$^+$) calcd for $C_{22}H_{25}N_3O_4$ $[M + H]^+$, 396.1923; found: 396.1930.

(*E*)-3-(4-aminostyryl)-2,5,6-trimethyl-1*H*,7*H*-pyrazolo[1,2-a]pyrazole-1,7-dione (5j): The deprotection of a Boc-protected **5i** (100 mg, 0.253 mmol, 1 equiv) was carried out under acidic

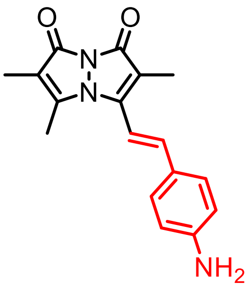

conditions using trifluoroacetic acid (432.5 mg, 3.79 mmol, 15 equiv) in 2 mL DCM solvent for 6 h. After completion of the reaction, the reaction progress was monitored by the TLC and concentrated the solvent and dried under vacuum to afford 62.8 mg of **5j** (84% isolated yield, red solid, R_f = 0.39). ^{1}H NMR (600 MHz, DMSO) δ 7.43 (d, J = 8.6 Hz, 2H), 7.13 (d, J = 16.3 Hz, 1H), 6.81 (d, J = 16.3 Hz, 1H), 6.60 (d, J = 8.5 Hz, 2H), 5.76 (s, 2H), 2.35 (s, 3H), 1.91 (s, 3H), 1.74 (s, 3H). ^{13}C NMR (151 MHz, DMSO) δ 161.6, 161.5, 151.7, 150.5, 149.9, 142.6, 130.0, 122.9, 114.1, 112.2, 109.8, 108.0, 12.6, 8.6, 7.0. HRMS (ESI$^+$) calcd for $C_{17}H_{17}N_3O_2$, $[M + H]^+$, 296.1399; found: 296.1405.

(*E*)-3-(4-(dimethylamino)styryl)-2,5,6-trimethyl-1*H*,7*H*-pyrazolo[1,2-a]pyrazole-1,7-dione

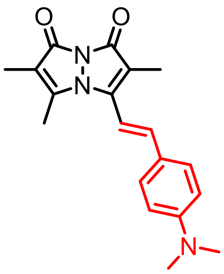

(5k). Following General Procedure B with 4-(dimethylamino)benzaldehyde (**4k**, 49.6 mg, 0.33 mmol, 1 equiv, purchased from Aldrich) at room temperature for 40 h afforded 46.3 mg (43 % isolated yield, orange solid) of **5k** after purification by column chromatography (3% methanol in dichloromethane, R_f = 0.45). ^{1}H NMR (600 MHz, $CDCl_3$) δ 7.40 (d, *J* = 8.9 Hz, 2H), 7.03 (d, *J* = 16.3 Hz, 1H), 6.71 (d, *J* = 8.9 Hz, 2H), 6.53 (d, *J* = 16.3 Hz, 1H), 3.04 (s, 6H), 2.29 (s, 3H), 2.01 (s, 3H), 1.84 (s, 3H). ^{13}C NMR (151 MHz, $CDCl_3$) δ 162.1, 162.0, 151.9, 149.0, 148.7, 141.8, 129.0, 122.8, 113.6, 112.2, 111.8, 108.4, 40.4, 12.8, 8.7, 7.2. HRMS (ESI$^+$) calcd for $C_{19}H_{21}N_3O_2$, $[M + H]^+$, 324.1712; found: 324.1712.

X-ray Structure Determination of Compound 5a

Compound **5a**, $C_{17}H_{15}N_3O_4$, crystallizes in the monoclinic space group $P2_1$ (systematic absences 0k0: k=odd) with a=3.85501(8)Å, b=13.3190(3)Å, c=14.7962(3)Å, β=94.880(2)°, V=756.96(3)Å^3, Z=2, and d_{calc}=1.427 g/cm^3 . X-ray intensity data were collected on a Rigaku XtaLAB Synergy-S diffractometer equipped with an HPC area detector (HyPix-6000HE) and employing confocal multilayer optic-monochromated Mo-Kα radiation (λ=0.71073 Å) at a temperature of 100 K. Preliminary indexing was performed from a series of thirty 0.5° rotation frames with exposures of 0.5 sec. A total of 1844 frames (11 runs) were collected employing ω scans with a crystal to detector distance of 34.0 mm, rotation widths of 0.5° and exposures of 5 sec. Rotation frames were integrated using CrysAlisPro, producing a listing of unaveraged F^2 and $\sigma(F^2)$ values. A total of 19219 reflections were measured over the ranges $4.122 \leq 2\theta \leq 50.682°$, $-4 \leq h \leq 4$, $-16 \leq k \leq 16$, $-17 \leq l \leq 17$ yielding 2782 unique reflections (R_{int} = 0.0272). The intensity data were corrected for Lorentz and polarization effects and for absorption using SCALE3 ABSPACK (minimum and maximum transmission 0.8029, 1.0000). The structure was solved by direct methods - ShelXT. Refinement was by full-matrix least squares based on F^2 using SHELXL-2018. All reflections were used during refinement. The weighting scheme used was $w=1/[\sigma^2(F_o^2)+ (0.0390P)^2 + 0.1383P]$ where $P = (F_o^2 + 2F_c^2)/3$. Non-hydrogen atoms were refined anisotropically and hydrogen atoms were refined using a riding model. Refinement converged to R1=0.0237 and wR2=0.0626 for 2734 observed reflections for which $F > 4\sigma(F)$ and R1=0.0241 and wR2=0.0629 and GOF =1.029 for all 2782 unique, non-zero reflections and 220 variables. The maximum Δ/σ in the final cycle of least squares was 0.000 and the two most prominent peaks in the final difference Fourier were +0.12 and -0.15 e/Å^3.

Table S1. lists cell information, data collection parameters, and refinement data. **Tables S2**. and **S3**. list bond distances and bond angles. **Fig. S2**. is an ORTEP representation of the molecule with 50% probability thermal ellipsoids displayed.

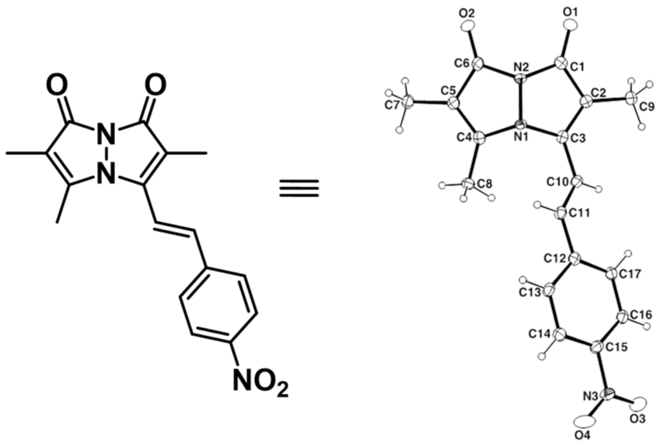

Fig. S2. X-ray diffraction data and ORTEP drawing of **5a** with 50% thermal ellipsoids.

Table S1. Summary of Structure Determination of **5a**

Empirical formula	$C_{17}H_{15}N_3O_4$
Formula weight	325.32
Diffractometer	Rigaku XtaLAB Synergy-S
Temperature/K	100
Crystal system	monoclinic
Space group	$P2_1$
a	3.85501(8)Å
b	13.3190(3)Å
c	14.7962(3)Å
β	94.880(2)°
Volume	756.96(3)Å^3
Z	2
d_{calc}	1.427 g/cm^3
μ	0.104 mm^{-1}
F(000)	340.0
Crystal size, mm	0.2 × 0.07 × 0.07
2θ range for data collection	4.122 - 50.682°
Index ranges	-4 ≤ h ≤ 4, -16 ≤ k ≤ 16, -17 ≤ l ≤ 17
Reflections collected	19219

Independent reflections	2782[R(int) = 0.0272]
Data/restraints/parameters	2782/1/220
Goodness-of-fit on F^2	1.029
Final R indexes [I>=2σ (I)]	R_1 = 0.0237, wR_2 = 0.0626
Final R indexes [all data]	R_1 = 0.0241, wR_2 = 0.0629
Largest diff. peak/hole	0.12/-0.15 eÅ^{-3}
Flack parameter	-0.3(3)

Table S2. Bond Distances in Compound **5a**, Å

C1-C2	1.456(3)	C1-N2	1.419(2)	C1-O1	1.216(2)
C2-C3	1.361(3)	C2-C9	1.491(3)	C3-C10	1.453(2)
C3-N1	1.416(2)	C4-C5	1.354(3)	C4-C8	1.484(2)
C4-N1	1.418(2)	C5-C6	1.450(3)	C5-C7	1.493(3)
C6-N2	1.435(2)	C6-O2	1.213(2)	C10-C11	1.345(3)
C11-C12	1.463(2)	C12-C13	1.405(3)	C12-C17	1.404(3)
C13-C14	1.384(3)	C14-C15	1.388(3)	C15-C16	1.388(3)
C15-N3	1.469(2)	C16-C17	1.381(3)	N1-N2	1.400(2)
N3-O3	1.230(2)	N3-O4	1.227(2)		

Table S3. Bond Angles in Compound **5a**, °

N2-C1-C2	105.09(15)	O1-C1-C2	130.24(18)	O1-C1-N2	124.61(17)
C1-C2-C9	121.50(16)	C3-C2-C1	108.59(17)	C3-C2-C9	129.84(18)
C2-C3-C10	127.42(17)	C2-C3-N1	109.79(16)	N1-C3-C10	122.17(16)
C5-C4-C8	127.80(17)	C5-C4-N1	109.57(16)	N1-C4-C8	122.51(16)
C4-C5-C6	109.23(16)	C4-C5-C7	128.84(17)	C6-C5-C7	121.91(16)
N2-C6-C5	104.84(15)	O2-C6-C5	131.29(17)	O2-C6-N2	123.87(17)
C11-C10-C3	123.89(16)	C10-C11-C12	126.40(17)	C13-C12-C11	117.83(16)
C17-C12-C11	123.28(17)	C17-C12-C13	118.89(17)	C14-C13-C12	121.02(17)
C13-C14-C15	118.21(17)	C14-C15-C16	122.51(17)	C14-C15-N3	118.40(17)
C16-C15-N3	119.07(16)	C17-C16-C15	118.66(17)	C16-C17-C12	120.70(17)
C3-N1-C4	130.54(15)	N2-N1-C3	106.78(14)	N2-N1-C4	107.10(14)
C1-N2-C6	128.21(15)	N1-N2-C1	109.73(13)	N1-N2-C6	109.04(14)
O3-N3-C15	118.26(17)	O4-N3-C15	118.47(16)	O4-N3-O3	123.26(17)

This report has been created with Olex2 [6], compiled on 2018.05.29 svn.r3508 for OlexSys.

X-ray Structure Determination of Compound 5c

Compound **5c**, $C_{17}H_{15.25}FN_2O_{2.125}$, crystallizes in the orthorhombic space group Fdd2 (systematic absences hkl: h+k=odd and k+l=odd, h0l: h+l≠4n, and 0kl: k+l≠4n) with a=29.9395(3)Å, b=27.5788(3)Å, c=7.07968(9)Å, V=5845.66(12)$Å^3$, Z=16, and d_{calc}=1.366 g/cm^3. X-ray intensity data were collected on a Rigaku XtaLAB Synergy-S diffractometer equipped with an HPC area detector (HyPix-6000HE) and employing confocal multilayer optic-monochromated Cu=Kα radiation (λ=1.54184 Å) at a temperature of 100K. Preliminary indexing was performed from a series of sixty 0.5° rotation frames with exposures of 15 sec. for θ = ±47.04° and 60 sec. for θ = 107.75°. A total of 7042 frames (47 runs) were collected employing ω scans with a crystal to detector distance of 34.0 mm, rotation widths of 0.5° and exposures of 2 sec. for θ = ±47.04° and 8 sec. for θ = -86.25 and 107.75°. Rotation frames were integrated using CrysAlisPro, producing a listing of unaveraged F^2 and $\sigma(F^2)$ values. A total of 35147 reflections were measured over the ranges $8.718 \leq 2\theta \leq 149.006°$, $-37 \leq h \leq 35$, $-33 \leq k \leq 34$, $-8 \leq l \leq 8$ yielding 2976 unique reflections (R_{int} = 0.0563). The intensity data were corrected for Lorentz and polarization effects and for absorption using SCALE3 ABSPACK (minimum and maximum transmission 0.58523, 1.00000). The structure was solved by dual space methods - SHELXT. Refinement was by full-matrix least squares based on F^2 using SHELXL. All reflections were used during refinement. The weighting scheme used was $w=1/[\sigma^2(F_o^2) + (0.0568P)^2 + 4.6832P]$ where $P = (F_o^2 + 2F_c^2)/3$. Non-hydrogen atoms were refined anisotropically and hydrogen atoms were refined using a riding model. Refinement converged to R1=0.0365 and wR2=0.0978 for 2938 observed reflections for which F > 4σ(F) and R1=0.0369 and wR2=0.0982 and GOF =1.087 for all 2976 unique, non-zero reflections and 207 variables. The maximum Δ/σ in the final cycle of least squares was 0.001 and the two most prominent peaks in the final difference Fourier were +0.15 and -0.21 e/$Å^3$.

Table S4. lists cell information, data collection parameters, and refinement data. **Tables S5** and **S6**. list bond distances and bond angles. **Figure S3**. is an ORTEP representation of the molecule with 50% probability thermal ellipsoids displayed.

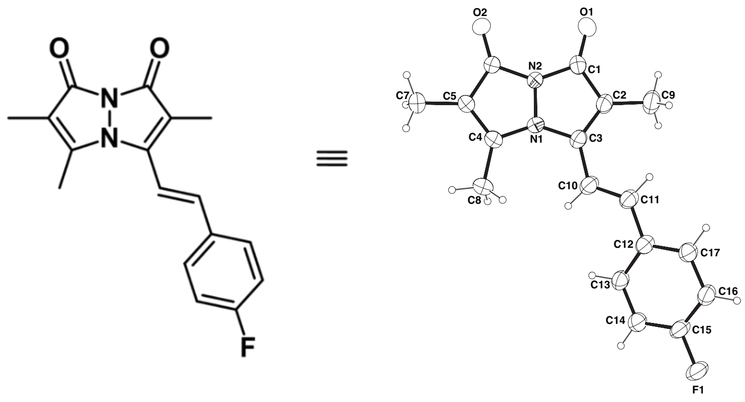

Fig. S3. X-ray diffraction data and ORTEP drawing of **5c** with 50% thermal ellipsoids.

Table S4. Summary of Structure Determination of **5c**

Empirical formula	$C_{17}H_{15.25}FN_2O_{2.125}$
Formula weight	300.56
Diffractometer	Rigaku XtaLAB Synergy-S (HyPix-6000HE)
Temperature/K	100
Crystal system	orthorhombic
Space group	Fdd2
a	29.9395(3)Å
b	27.5788(3)Å
c	7.07968(9)Å
α	90°
β	90°
γ	90°
Volume	5845.66(12)Å^3
Z	16
d_{calc}	1.366 g/cm^3
μ	0.829 mm^{-1}
F(000)	2516.0
Crystal size, mm	0.25 × 0.12 × 0.08
2θ range for data collection	8.718 - 149.006°
Index ranges	-37 ≤ h ≤ 35, -33 ≤ k ≤ 34, -8 ≤ l ≤ 8
Reflections collected	35147

Independent reflections	2976[R(int) = 0.0563]
Data/restraints/parameters	2976/1/207
Goodness-of-fit on F^2	1.087
Final R indexes [I>=2σ (I)]	R_1 = 0.0365, wR_2 = 0.0978
Final R indexes [all data]	R_1 = 0.0369, wR_2 = 0.0982
Largest diff. peak/hole	0.15/-0.21 eÅ^{-3}
Flack parameter	0.10(6)

Table S5. Bond Distances in Compound **5c**, Å

C1-C2	1.452(3)	C1-N2	1.417(3)	C1-O1	1.221(3)
C2-C3	1.374(3)	C2-C9	1.490(3)	C3-C10	1.450(3)
C3-N1	1.387(3)	C4-C5	1.368(3)	C4-C8	1.483(3)
C4-N1	1.374(3)	C5-C6	1.448(3)	C5-C7	1.487(3)
C6-N2	1.398(3)	C6-O2	1.219(3)	C10-C11	1.346(3)
C11-C12	1.464(3)	C12-C13	1.403(3)	C12-C17	1.392(3)
C13-C14	1.388(3)	C14-C15	1.380(3)	C15-C16	1.371(3)
C15-F1	1.357(3)	C16-C17	1.392(3)	N1-N2	1.375(3)

Table S6. Bond Angles in Compound **5c**, °

N2-C1-C2	104.22(18)	O1-C1-C2	131.6(2)	O1-C1-N2	124.2(2)
C1-C2-C9	121.2(2)	C3-C2-C1	109.11(19)	C3-C2-C9	129.5(2)
C2-C3-C10	130.4(2)	C2-C3-N1	108.07(19)	N1-C3-C10	121.5(2)
C5-C4-C8	130.0(2)	C5-C4-N1	108.1(2)	N1-C4-C8	122.0(2)
C4-C5-C6	109.28(19)	C4-C5-C7	127.9(2)	C6-C5-C7	122.8(2)
N2-C6-C5	103.76(18)	O2-C6-C5	131.4(2)	O2-C6-N2	124.86(19)
C11-C10-C3	121.2(2)	C10-C11-C12	127.4(2)	C13-C12-C11	123.9(2)
C17-C12-C11	117.1(2)	C17-C12-C13	118.9(2)	C14-C13-C12	120.3(2)
C15-C14-C13	118.5(2)	C16-C15-C14	123.2(2)	F1-C15-C14	118.2(2)
F1-C15-C16	118.6(2)	C15-C16-C17	117.7(2)	C12-C17-C16	121.4(2)
C4-N1-C3	142.1(2)	C4-N1-N2	108.70(18)	N2-N1-C3	109.15(18)
C6-N2-C1	140.38(18)	N1-N2-C1	109.45(18)	N1-N2-C6	110.16(17)

This report has been created with Olex2 [6], compiled on 2021.10.07 svn.r9718050b for OlexSys.

X-ray Structure Determination of Compound 5k

Compound **5k**, $C_{20}H_{25}N_3O_3$, crystallizes in the monoclinic space group $P2_1/c$ (systematic absences 0k0: k=odd and h0l: l=odd) with a=7.0514(6)Å, b=17.1123(10)Å, c=30.720(2)Å, β=91.352(8)°, V=3705.8(5)Å^3, Z=8, and d_{calc}=1.274 g/cm^3. X-ray intensity data were collected on a Rigaku XtaLAB Synergy-S diffractometer equipped with an HPC area detector (Dectris Pilatus3 R 200K) and employing confocal multilayer optic-monochromated Mo-Kα radiation (λ=0.71073 Å) at a temperature of 100 K. Preliminary indexing was performed from a series of thirty 0.5° rotation frames with exposures of 10 seconds. A total of 1272 frames (13 runs) were collected employing ω scans with a crystal to detector distance of 50.0 mm, rotation widths of 0.5° and exposures of 60 seconds.

The crystal grew as a non-merohedral twin. The Ewald Explorer extension in CrysAlisPro was used to index the diffraction images and to determine the twinning mechanism. The crystal was twinned by a rotation of 180° about the 001 real direction. Rotation frames were integrated using CrysAlisPro, producing a listing of unaveraged F^2 and $\sigma(F^2)$ values. A total of 49137 reflections were measured over the ranges $4.636 \leq 2\theta \leq 52.95°$, $-8 \leq h \leq 8$, $-21 \leq k \leq 21$, $-37 \leq l \leq 38$ yielding 13556 unique reflections (R_{int} = 0.081). The intensity data were corrected for Lorentz and polarization effects and for absorption using SCALE3 ABSPACK (minimum and maximum transmission 0.85,80 1.0000). The structure was solved by dual space methods - SHELXT. Refinement was by full-matrix least squares based on F^2 using SHELXL. All reflections were used during refinement. The weighting scheme used was $w=1/[\sigma^2(F_o^2)+ (0.1260P)^2 + 0.9687P]$ where $P = (F_o^2 + 2F_c^2)/3$. Non-hydrogen atoms were refined anisotropically and hydrogen atoms were refined using a riding model. Refinement converged to R1=0.0719 and wR2=0.1949 for 9577 observed reflections for which $F > 4\sigma(F)$ and R1=0.1047 and wR2=0.2144 and GOF =1.047 for

all 13556 unique, non-zero reflections and 484 variables. The maximum Δ/σ in the final cycle of least squares was 0.000 and the two most prominent peaks in the final difference Fourier were +0.40 and -0.33 e/Å^3. The twinning parameter refined to a value of 0.5079(16).

Table S7. lists cell information, data collection parameters, and refinement data. **Tables S8**. and **S9**. list bond distances and bond angles. **Fig. S4**. is an ORTEP representation of the molecule with 50% probability thermal ellipsoids displayed.

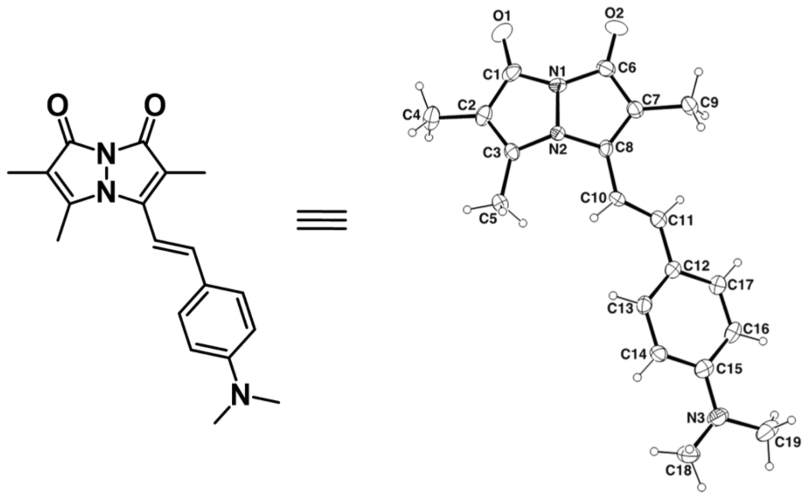

Fig. S4. X-ray diffraction data and ORTEP drawing of **5k** with 50% thermal ellipsoids.

Table S7. Summary of Structure Determination of Compound **5c**

Empirical formula	$C_{20}H_{25}N_3O_3$
Formula weight	355.43
Diffractometer	Rigaku XtaLAB Synergy-S (Dectris Pilatus3 R 200K)
Temperature/K	100
Crystal system	monoclinic
Space group	$P2_1/c$
a	7.0514(6)Å
b	17.1123(10)Å
c	30.720(2)Å
β	91.352(8)°
Volume	3705.8(5)Å^3
Z	8
d_{calc}	1.274 g/cm^3
μ	0.087 mm^{-1}

F(000)	1520.0
Crystal size, mm	0.26 × 0.19 × 0.04
2θ range for data collection	4.636 - 52.95°
Index ranges	-8 ≤ h ≤ 8, -21 ≤ k ≤ 21, -37 ≤ l ≤ 38
Reflections collected	49137
Independent reflections	13556[R(int) = 0.081]
Data/restraints/parameters	13556/0/484
Goodness-of-fit on F^2	1.047
Final R indexes [I>=2σ (I)]	R_1 = 0.0719, wR_2 = 0.1949
Final R indexes [all data]	R_1 = 0.1047, wR_2 = 0.2144
Largest diff. peak/hole	0.40/-0.33 eÅ^{-3}

Table S8. Bond Distances in Compound **5k**, Å

O1-C1	1.235(4)	O2-C6	1.219(4)	N1-N2	1.369(4)
N1-C1	1.409(4)	N1-C6	1.421(4)	N2-C3	1.392(4)
N2-C8	1.394(4)	N3-C15	1.373(4)	N3-C18	1.438(5)
N3-C19	1.455(5)	C1-C2	1.430(5)	C2-C3	1.357(4)
C2-C4	1.510(5)	C3-C5	1.477(5)	C6-C7	1.437(5)
C7-C8	1.364(5)	C7-C9	1.508(4)	C8-C10	1.445(4)
C10-C11	1.339(4)	C11-C12	1.453(4)	C12-C13	1.391(5)
C12-C17	1.401(4)	C13-C14	1.378(5)	C14-C15	1.398(5)
C15-C16	1.414(5)	C16-C17	1.380(5)	O1'-C1'	1.215(4)
O2'-C6'	1.225(4)	N1'-N2'	1.370(4)	N1'-C1'	1.404(4)
N1'-C6'	1.401(4)	N2'-C3'	1.385(4)	N2'-C8'	1.378(4)
N3'-C15'	1.362(5)	N3'-C18'	1.445(5)	N3'-C19'	1.459(4)
C1'-C2'	1.459(5)	C2'-C3'	1.348(4)	C2'-C4'	1.490(5)
C3'-C5'	1.482(4)	C6'-C7'	1.445(5)	C7'-C8'	1.373(5)
C7'-C9'	1.490(5)	C8'-C10'	1.444(5)	C10'-C11'	1.347(4)
C11'-C12'	1.452(5)	C12'-C13'	1.398(5)	C12'-C17'	1.403(4)
C13'-C14'	1.367(5)	C14'-C15'	1.422(4)	C15'-C16'	1.409(5)
C16'-C17'	1.369(5)	O3-C20	1.404(5)	O3'-C20'	1.372(5)

Table S9. Bond Angles in Compound **5k**, °

N2-N1-C1	109.1(3)	N2-N1-C6	109.6(2)	C1-N1-C6	139.1(3)
N1-N2-C3	108.6(2)	N1-N2-C8	108.4(3)	C3-N2-C8	140.0(3)
C15-N3-C18	120.4(3)	C15-N3-C19	120.1(3)	C18-N3-C19	117.6(3)
O1-C1-N1	122.9(3)	O1-C1-C2	132.3(3)	N1-C1-C2	104.8(3)
C1-C2-C4	123.6(3)	C3-C2-C1	109.4(3)	C3-C2-C4	127.0(3)
N2-C3-C5	121.6(3)	C2-C3-N2	108.0(3)	C2-C3-C5	130.4(3)
O2-C6-N1	123.6(3)	O2-C6-C7	132.1(3)	N1-C6-C7	104.3(3)
C6-C7-C9	122.0(3)	C8-C7-C6	109.3(3)	C8-C7-C9	128.5(3)
N2-C8-C10	120.6(3)	C7-C8-N2	108.3(3)	C7-C8-C10	131.0(3)
C11-C10-C8	122.6(3)	C10-C11-C12	126.5(3)	C13-C12-C11	123.7(3)
C13-C12-C17	116.6(3)	C17-C12-C11	119.7(3)	C14-C13-C12	122.5(3)
C13-C14-C15	121.0(3)	N3-C15-C14	121.0(3)	N3-C15-C16	121.9(3)
C14-C15-C16	117.1(3)	C17-C16-C15	121.0(3)	C16-C17-C12	121.9(3)
N2'-N1'-C1'	109.9(3)	N2'-N1'-C6'	109.7(2)	C6'-N1'-C1'	139.8(3)
N1'-N2'-C3'	108.5(2)	N1'-N2'-C8'	109.1(3)	C8'-N2'-C3'	141.9(3)
C15'-N3'-C18'	120.9(3)	C15'-N3'-C19'	121.4(3)	C18'-N3'-C19'	117.6(3)
O1'-C1'-N1'	124.5(3)	O1'-C1'-C2'	131.7(3)	N1'-C1'-C2'	103.7(3)
C1'-C2'-C4'	122.7(3)	C3'-C2'-C1'	109.1(3)	C3'-C2'-C4'	128.1(3)
N2'-C3'-C5'	122.2(3)	C2'-C3'-N2'	108.7(3)	C2'-C3'-C5'	129.1(3)
O2'-C6'-N1'	124.2(3)	O2'-C6'-C7'	131.5(3)	N1'-C6'-C7'	104.2(3)
C6'-C7'-C9'	121.2(3)	C8'-C7'-C6'	109.1(3)	C8'-C7'-C9'	129.1(3)
N2'-C8'-C10'	120.6(3)	C7'-C8'-N2'	107.8(3)	C7'-C8'-C10'	131.6(3)
C11'-C10'-C8'	124.1(3)	C10'-C11'-C12'	126.1(3)	C13'-C12'-C11'	123.4(3)
C13'-C12'-C17'	116.7(3)	C17'-C12'-C11'	119.9(3)	C14'-C13'-C12'	122.0(3)
C13'-C14'-C15'	121.3(3)	N3'-C15'-C14'	120.7(3)	N3'-C15'-C16'	122.7(3)
C16'-C15'-C14'	116.5(3)	C17'-C16'-C15'	121.1(3)	C16'-C17'-C12'	122.3(3)

This report has been created with Olex2 [6], compiled on 2021.08.20 svn.r13c46975 for OlexSys.

Table S10. Comparison of selected bond lengths and bond angles of **5a**, **5c**, and **5k**.

probe	bond length (Å)		bond angles °	packing
	C1-O1	N2-N1	φ (C1-N2-C6)	
5a	1.216(2)	1.400(2)	128.21(15)	face-to-face
5c	1.221(3)	1.375(3)	140.38(18)	head-to-tail
5k	1.235(4)	1.369(4)	139.1(3)	head-to-tail

Table S11. Crystal structures of probes **5a**, **5c**, and **5k** showing their asymmetric units and representative sections of their crystal lattices (ORTEP-style with 50% thermal ellipsoids).

Probe	Asymmetric unit[a]	Side view	Top view
5a	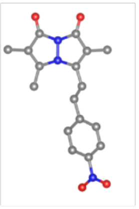	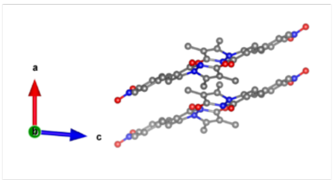	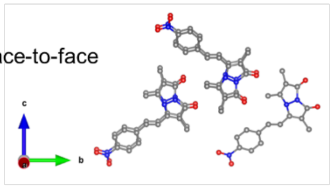
5c	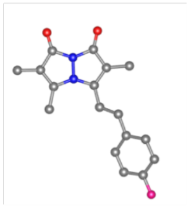	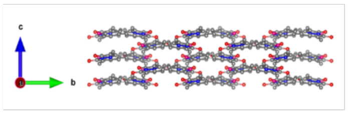	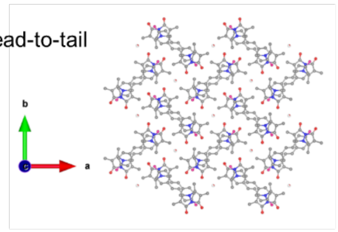
5k[b]	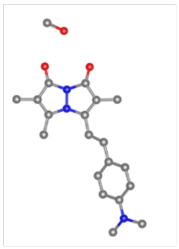	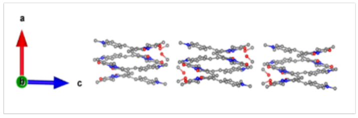	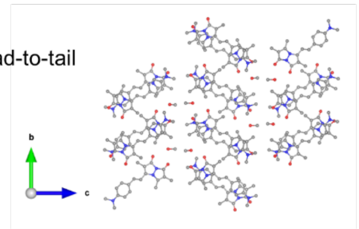

[a]Probes **5a**, **5c**, and **5k** exhibit several molecules per asymmetric unit, only one of which shown here. [b]Probe **5k** includes interstitial methanol molecules. Atoms are colored as follows: C, gray; N, blue; O, red; F, pink. Hydrogen atoms have been omitted for clarity.

General Photophysical Characterization Methods

Absorbance measurements. Key precursor **3** and bimane derivatives **5a-5k** were dissolved in DMSO to make 10 mM starting stock solutions. Samples were individually diluted to the final concentration of 25 μM (500 μL) using 50:50 acetonitrile in phosphate buffered saline (ACN/PBS). The absorbance spectrum of each derivative (150 μL) was measured on a Thermo Scientific Genesys 150 UV-Vis spectrometer, using 50:50 ACN/PBS as a blank.

Fluorescence measurements. 10 mM stock solutions of **3** and **5a-5k** in DMSO were individually diluted to the final concentration of 25 μM (500 μL) using 50:50 ACN/PBS. The fluorescence spectrum of each derivative (150 μL) was measured on a Photon Technology International (PTI) QuantaMaster™ 40 fluorescence spectrometer by excitation at the maximum absorption wavelength for each compound.

Molar absorptivity determination. 10 mM stock solutions of **3** and **5a-5k** in DMSO were individually diluted to the final concentration of 25 μM (500 μL) using 50:50 ACN/PBS. The absorbance spectrum of each derivative (150 μL) was measured on a Thermo Scientific Genesys 150 UV-Vis spectrometer, using 50:50 ACN/PBS as a blank. Then, the molar absorptivity of each derivative was calculated by using the Beer–Lambert law for solutions, $A = \varepsilon lc$, where A = absorbance at maximum wavelength, l = optical path length in cm, c = concentration of the solution (25 μM), ε = molar absorptivity.

Solvatochromic measurements. 10 mM stock solution of **3**, **5b**, **5d**, **5j**, and **5k** in DMSO were individually diluted into six different organic solvents (toluene, dichloromethane, ACN, dimethyl formamide, dimethyl sulfoxide, and ethanol) to the final concentration of 25 μM (1000 μL). The fluorescence spectrum of each derivative (150 μL) was measured on a PTI QuantaMaster™ 40 fluorescence spectrometer by excitation at the maximum absorption wavelength for each compound. The remaining 850 μL solution was used to obtain fluorescence images under a handheld 365 nm UV lamp.

pH measurements. 10 mM stock solutions of **5h** and **5j** in DMSO were individually diluted into ACN/PBS buffer (50:50 v/v) having different pH ranging from pH 2 – 11 with 25 μM (500 μL) of final concentration. For pH screening of **5h** and **5j**, PBS buffer solutions were prepared, then adjusted the pH 2, 4, and 7 by adding 6 M hydrochloric acid, pH 9 and 11 by adding 5 M sodium hydroxide. Then, we have recorded the absorption and fluorescence spectra by using Thermo Scientific Genesys 150 UV-Vis spectrometer, followed by PTI QuantaMaster™ 40 fluorescence spectrometer by excitation with maximum absorption wavelength.

Viscosity measurements. As a representative example, we have measured the viscosity of **5k** (25 μM, 10 mM stock in DMSO) in glycerol/ethylene glycol (G/EG) mixtures, varying the glycerol percentage 0-90%. Then, the emission spectra were recorded using a PTI QuantaMaster™ 40 fluorescence spectrometer by excitation with 385 nm wavelength.

Photophysical Properties of 3 and 5a-5k in ACN

Table S12. Photophysical properties of the bimane derivatives 3 and 5a-5k in ACN.

Entry	Substituent (Ph-R)	Absorbance λ_{max}(nm)[a]	Emission λ_{max} (nm)[b]	Molar Absorptivity (ε)($M^{-1}cm^{-1}$)[c]	Stokes shift (nm)[d]	Fluorescence quantum yield (%)[e]
**3**	–	375	432	6121	57	77.7
**5a**	NO_2	332	432	37871	100	0.2
**5b**	CN	316	522	43238	206	1.4
**5c**	F	315	492, 520	28940	177	2.8
**5d**	H	316	492, 522	32724	176	3.1
**5e**	CH_3	325	490, 520	24426	165	3.4
**5f**	OCH_3	342	488, 518	34554	146	3.5
**5h**	OH	342	486, 518	29910	144	3.1
**5i**	NH(Boc)	348	490, 520	37555	142	3.4
**5j**	NH_2	378	540	32091	162	1.5
**5k**	NMe_2	402	568	24742	166	7.5

[a]Maximum absorption wavelength, [b]Maximum emission wavelength, [c]Molar absorption coefficients at maximum absorption wavelength, [d]Difference between maximum absorption wavelength and maximum emission wavelength, [e]Fluorescence quantum yield (error limit within ±5). Final probe concentration is 25 μM in ACN.

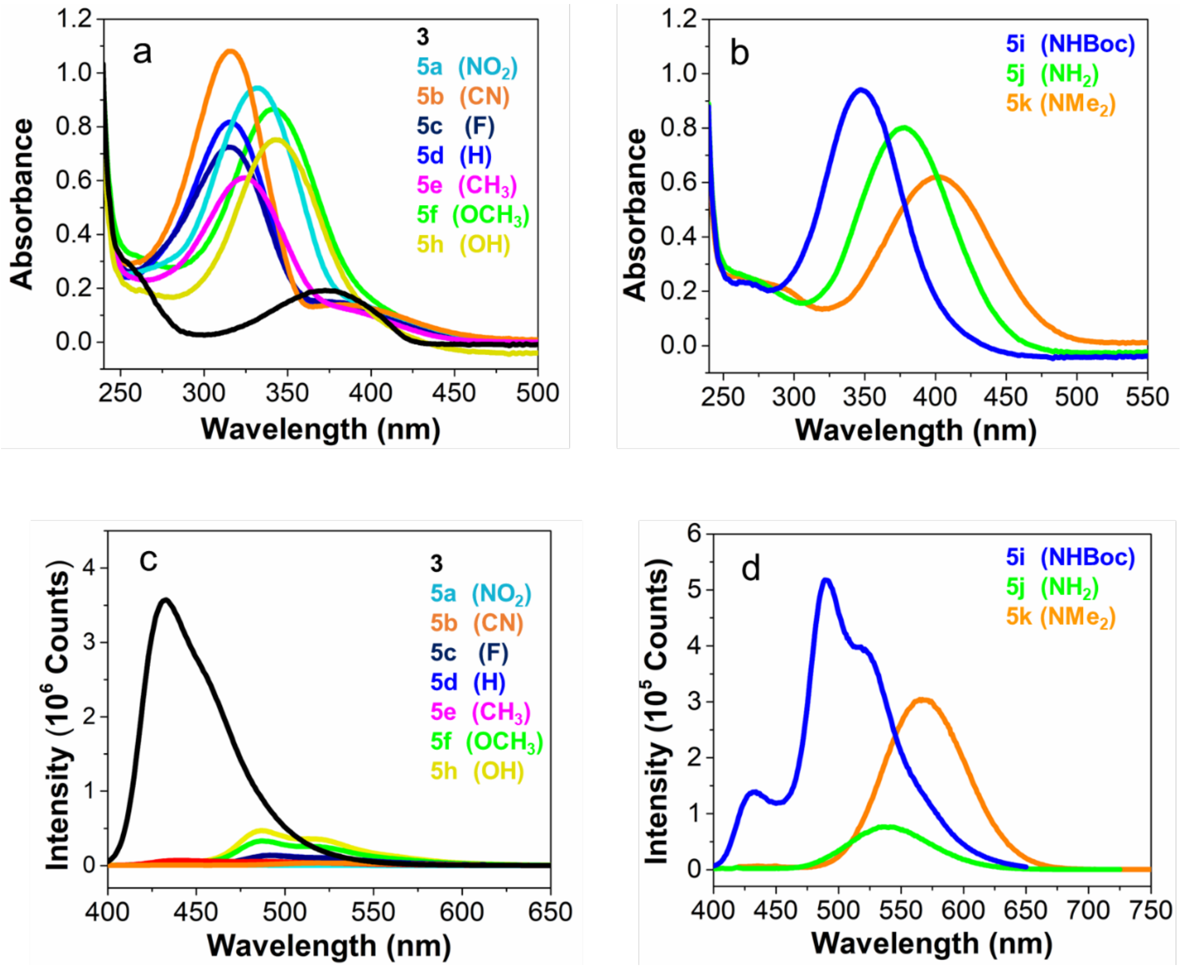

Fig. S5. Photophysical properties of **3** and **5a-5k** in ACN solvent. (a) Absorption spectra of **3** and **5a-5f** and **5h** (b) Absorption spectra of **5i-5k**, (c) Fluorescence spectra of **3** and **5a-5f** and **5h**, and (d) Fluorescence spectra of **5i-5k**. Final probe concentration is 25 μM. Fluorescence spectra were measured at their maximum absorption wavelength.

Solvatochromism of 3, 5b, 5d and **5j**

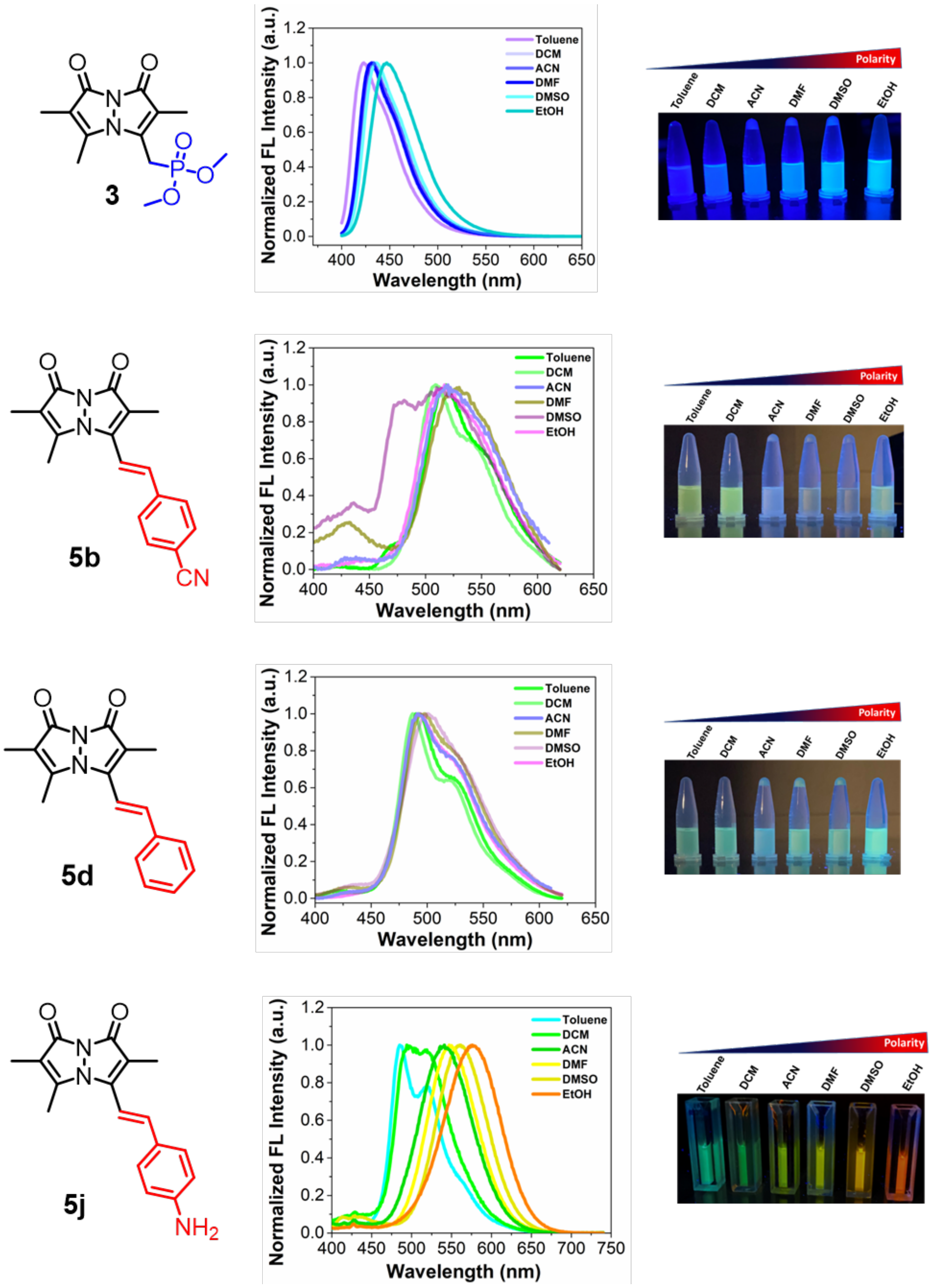

Fig. S6. Solvatochromism of **3, 5b, 5d** and **5j**. Left: Normalized fluorescence spectra in different solvents. Right: Fluorescence image taken under handheld UV lamp at 365 nm. Probe concentration 25 μM and excitation wavelength is 384 nm for **3**, 320 nm for **5b** and **5d,** and 418 nm for **5j**.

Fluorescent Quantum Yield Measurements of 3 and 5a-5k

Fluorescent quantum yield (QY) measurements. For each QY measurement, the incident excitation light spectrum was collected with 1 mL of solvent. After measuring the incident light intensity, dye (**3** and **5a-5k**) was added to the solvent from a concentrated stock solution to a final concentration of 500 μM and the new spectrum (fluorescence intensity and new incident light intensity) was collected. Using the JASCO Quantum Yield Software, the dye QY was calculated by dividing the dye fluorescence intensity by the difference in incident light intensity in the presence and absence of dye. The minimum excitation wavelength for a full excitation incident spectrum using this setup is 360 nm. QY values are reported in Table S1 and in main text Table 2.

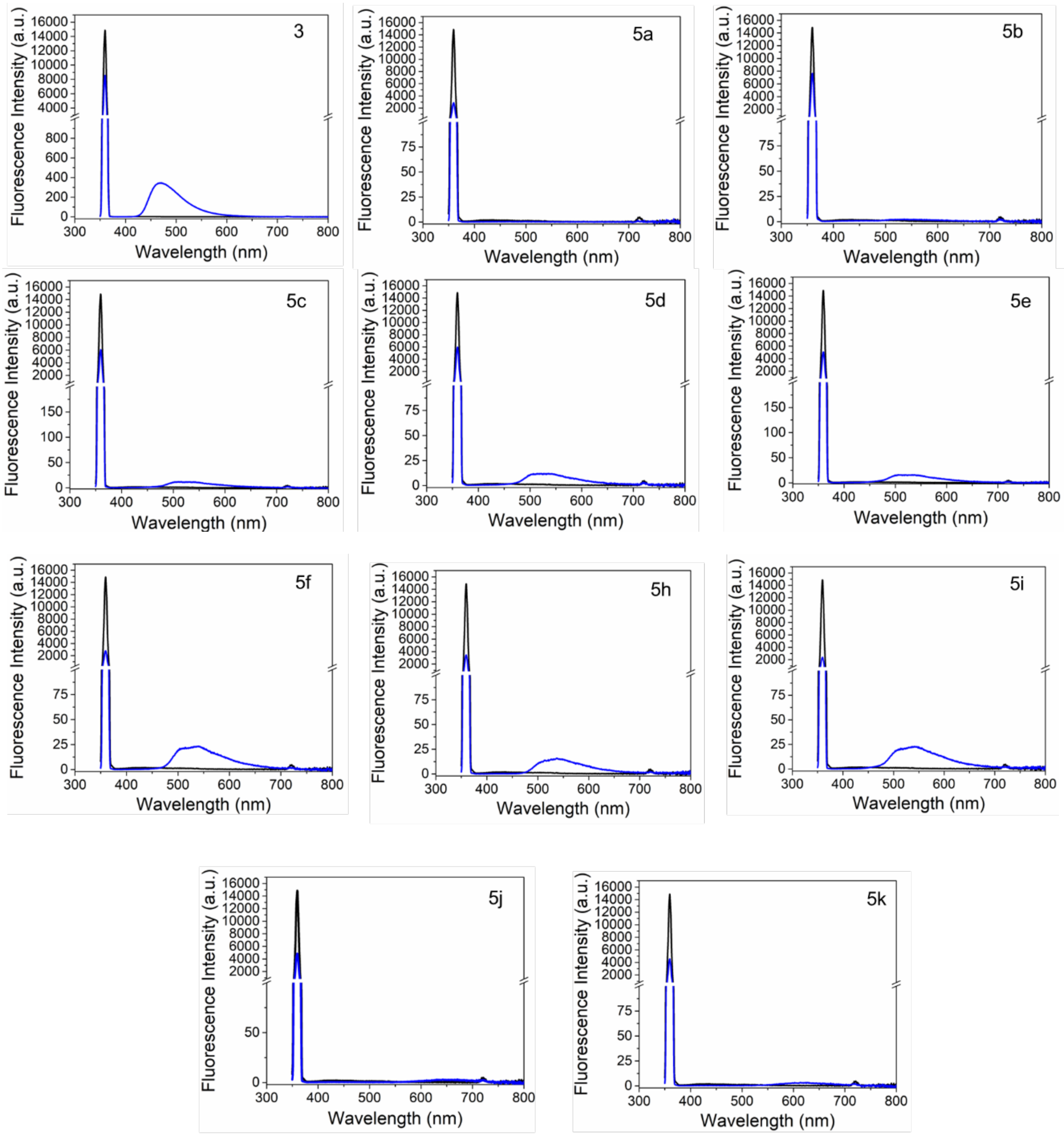

Fig. S7. Representative QY acquisitions of **3** and **5a-f, 5h-k** in ACN/PBS buffer (50:50). All had consistent excitation (360 nm) and spectral collection (350–800 nm) parameters. Final concentration of the probe is 500 μM. Black line indicates incident light and blue line indicates sample spectrum.

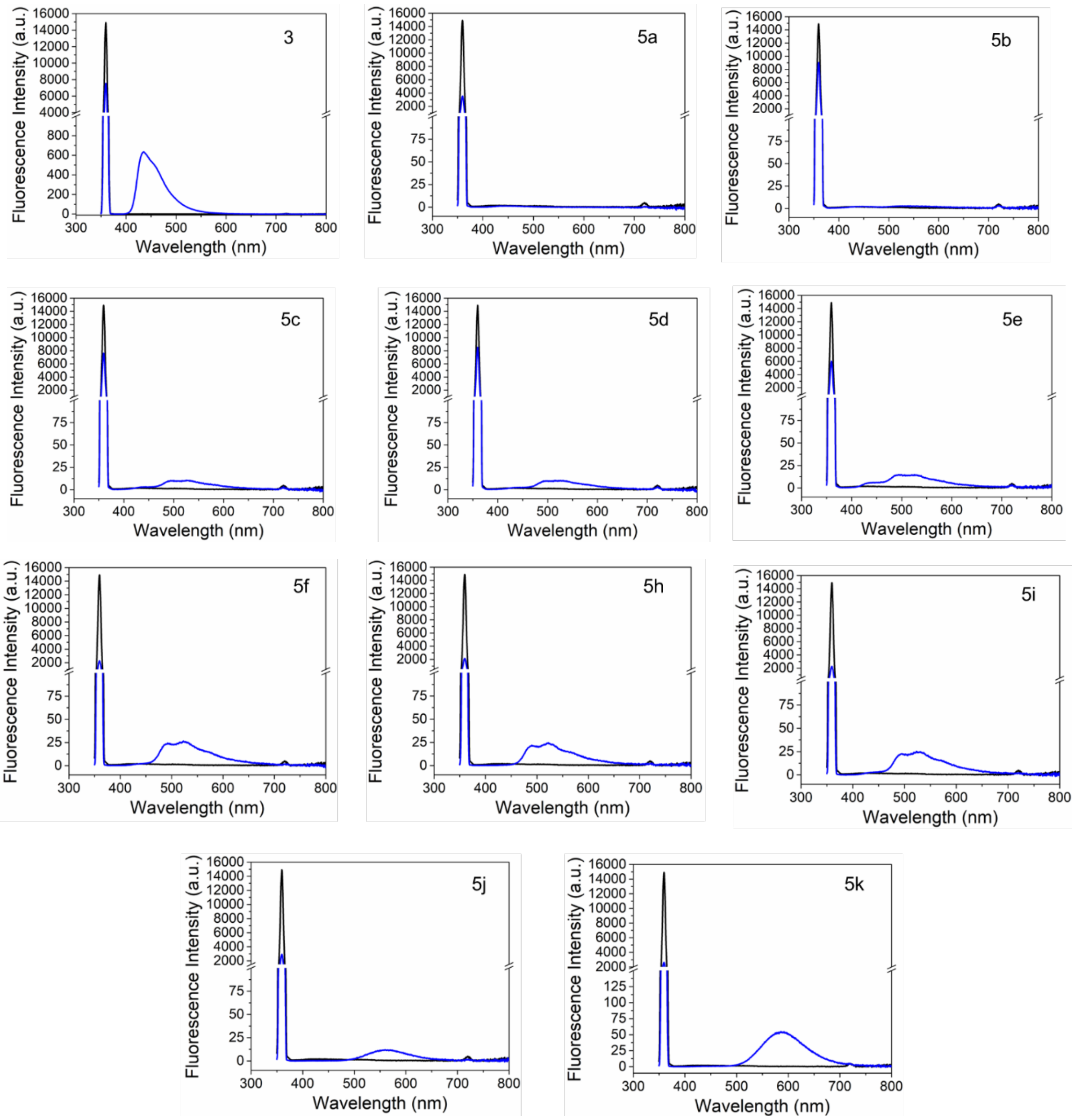

Fig. S8. Representative QY acquisitions of **3** and **5a-f, 5h-k** in ACN solvent. All had consistent excitation (360 nm, slit width 10 nm) and spectral collection (350-800 nm) parameters. Final concentration of the probe is 500 μM. Black line indicates incident light and blue line indicates sample spectrum.

Effect of pH on 5h and 5j

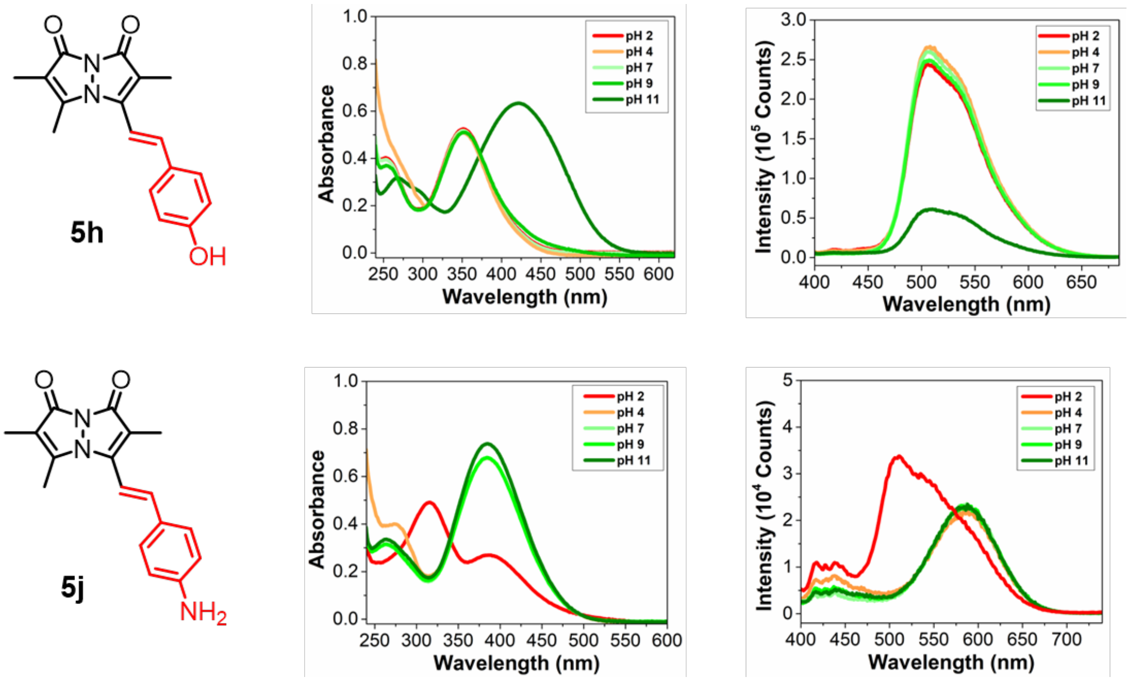

Fig. S9. Effect of pH on absorption and emission of **5h** and **5j**. Final concentration of the probe is 25 μM. For pH screening of **5h** and **5j,** PBS buffer solutions were prepared, then adjusted the pH 2, 4, and 7 by adding 6 M hydrochloric acid, pH 9 and 11 by adding 5 M sodium hydroxide. Emission spectra were measured by the excitation wavelength 354 nm for **5h** and 385 nm for **5j**.

Fluorescence Lifetime Measurements of 3 and 5c-5f

Fluorescence lifetime measurements. Time correlated single photon counting (TCSPC) measurements of fluorescence lifetime decays for 100 μM solutions of dyes (**3 and 5c-f**) in ACN/PBS buffer (50:50 v/v) were collected with the PTI Quantamaster TM 40 using a pulsed LED with a maximum emission at 340 nm. Fluorescence emission was collected at the indicated wavelength for each dye with 20 nm slit widths. The instrument response function (IRF) was collected under identical conditions. Data analysis was performed with FluoFit software (PicoQuant GmbH; Berlin, Germany) using an exponential decay model. Lifetime values are reported in Table S13 and in main text Table 2.

Table S13. Fluorescence lifetime measurements of **3** and selected probes **5c-f** in ACN/PBS buffer (50:50 v/v) and fit χ^2 values.[a]

Probe	Ar–R	Lifetime (ns)	χ^2
**3**	-	2.32 ± 0.01	1.099
**5c**	F	1.07 ± 0.003	1.017
**5d**	H	1.02 ± 0.004	0.942
**5e**	CH_3	1.28 ± 0.004	1.089
**5f**	OCH_3	1.31 ± 0.004	0.932

[a]TCSPC data were collected at 540 nm emission for **5c-f** and 464 nm emission for **3**. Excitation wavelength 340 nm.

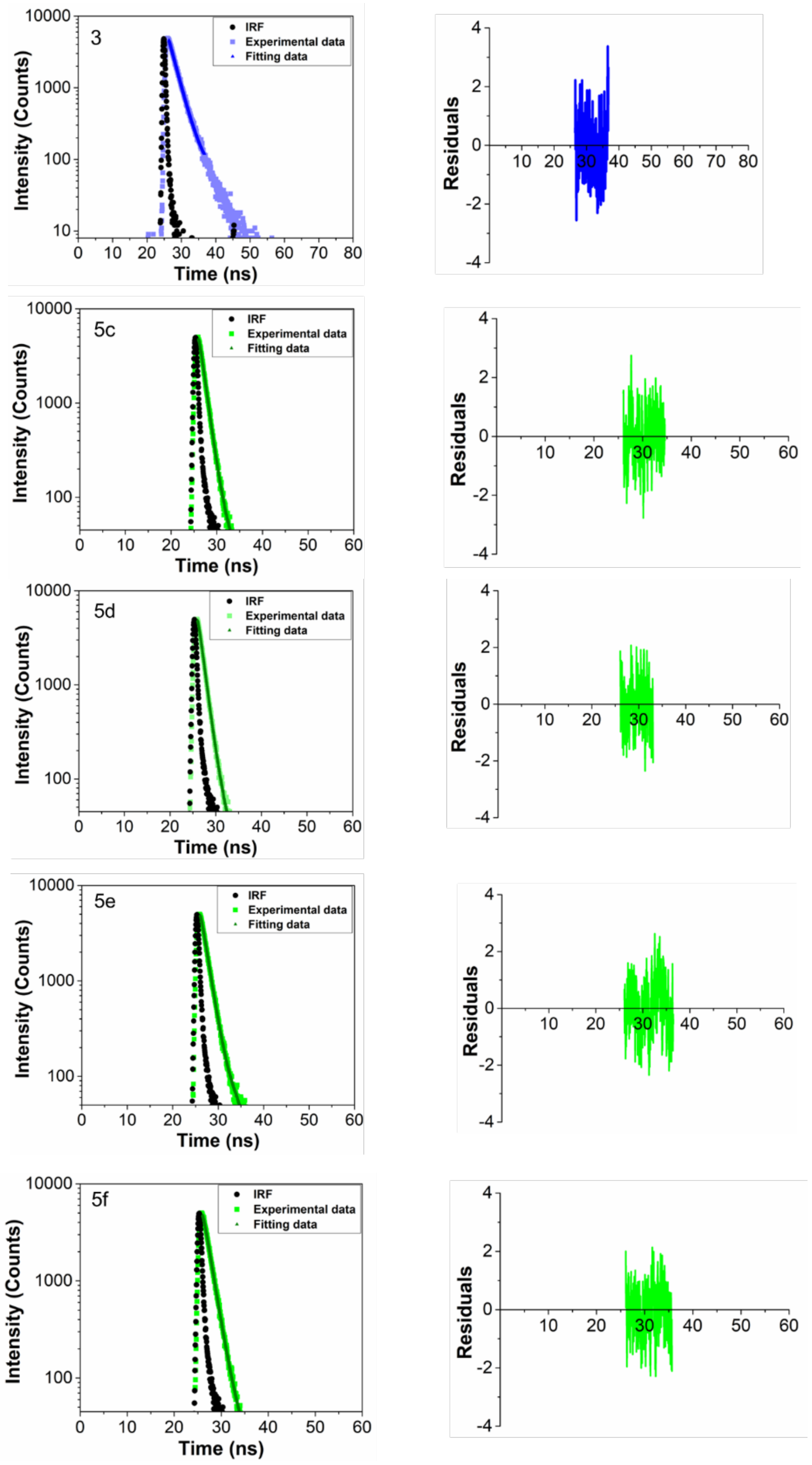

Fig. S10. Fluorescence lifetime measurements in ACN/PBS buffer (50:50 v/v). Lifetime measurements with exponential fits shown at left and residuals after fitting shown at right. TCSPC data were collected at 540 nm emission for **5c-5f** and 464 nm emission for **3**. Excitation wavelength 340 nm.

Computational Studies

Methods. All calculations were performed employing the APF-D density functional as implemented in the Gaussian16™ suite of programs with the 6-311+G (2d,p) basis set.[3] Geometry optimizations and energies were calculated for selected bimane derivatives **5a-f**, **5h**, **5j**, and **5k** compounds. The HOMO-LUMO energy gaps between the ground state (S_0) and the first excited state (S_1) of the bimane derivatives were calculated and are shown below. All structures are ground-state minima according to the analysis of their vibrational frequencies, which showed no negative value.

Table S14. Calculation of the Energy Gaps of Bimane Derivatives in Water (H_2O).

Compound	Hammett Constant (σ_p)	Absorbance Wavelength (nm)[a]	HOMO (eV)	LUMO (eV)	#LUMO-#HOMO Transition	LUMO-HOMO Δ(eV)
**5a (NO$_2$)**	0.778	335	-6.547	-3.232	86-85	3.315
**5b (CN)**	0.66	320	-6.523	-2.827	81-80	3.696
**5c (F)**	0.062	321	-6.419	-2.506	79-78	3.912
**5d (H)**	0	321	-6.430	-2.514	75-74	3.915
**5e (CH$_3$)**	-0.17	332	-6.351	-2.470	79-78	3.881
**5f (OCH$_3$)**	-0.268	348	-6.146	-2.420	83-82	3.726
**5h (OH)**	-0.37	354	-6.192	-2.420	79-78	3.772
**5j (NH$_2$)**	-0.66	385	-5.812	-2.383	79-78	3.429
**5k (NMe$_2$)**	-0.83	418	-5.528	-2.356	87-86	3.172

[a]Absorbance spectra recorded in ACN:PBS buffer (50:50 v/v).

HOMO-LUMO gap calculations. We have analyzed the correlation between the photophysical properties of bimane derivatives and the electronic properties of the substituents to understand the mechanism of fluorescence tuning of the bimane scaffold. It is generally accepted that the absorption wavelength (λ_{abs}) and emission wavelength (λ_{em}) correlate well with the energy gap between HOMO and LUMO as determined by simple electronic structure calculations. Relative energy levels of HOMO and LUMO were calculated using APF-D/6-311+G (2d,p) density functional theory (DFT) calculations. Representative compounds **5e** (CH_3), **5f (**OCH_3), **5h** (OH), **5j** (NH_2), and **5k** (NMe_2), with electron donating groups at the *para*-position, showed a decrease

in HOMO-LUMO gap with more electron donating substituents that matched well with red-shifts of the UV-Vis absorbance (Table S3 and Fig. S10). Interestingly, compounds with electron withdrawing substituents, **5a** (NO_2)**, 5b** (CN), and **5c** (F) showed a shallower correlation of UV-Vis absorbance with HOMO-LUMO gap, indicative of a change in excitation mechanism (Fig. S10). The change in mechanism can be explained by examination of the HOMO and LUMO of these compounds (Fig. S11). For compounds with electron-withdrawing substituents such as **5a**, the electrons move from a HOMO centered on the bimane group to a LUMO centered on the styryl group. In contrast, for compounds with electron-donating substituents such as **5k**, the electrons move from a HOMO centered on the styryl group to a LUMO centered on the bimane group. This is similar to our observations of the changes in λ_{abs} with the Hammett constant in **Fig. 1** in the main text.

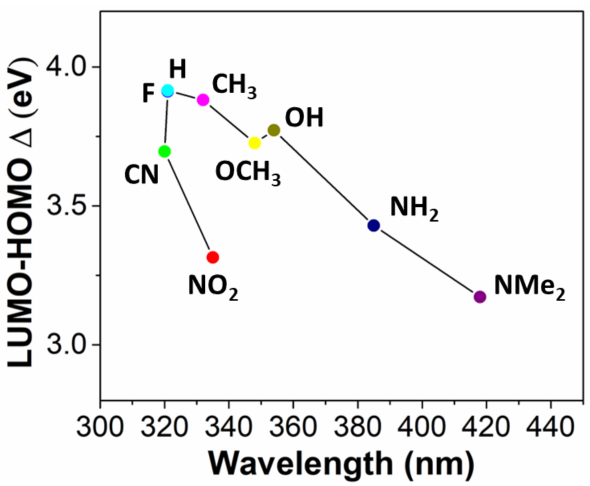

Fig. S11. Correlation between HOMO-LUMO energy gap (eV) compared to experimentally measured UV-Vis absorption maximum (nm) of **5a-5f**, **5h**, **5j**, and **5k**. Maximum UV-Vis absorption wavelengths were obtained in ACN/PBS buffer (50:50 v/v).

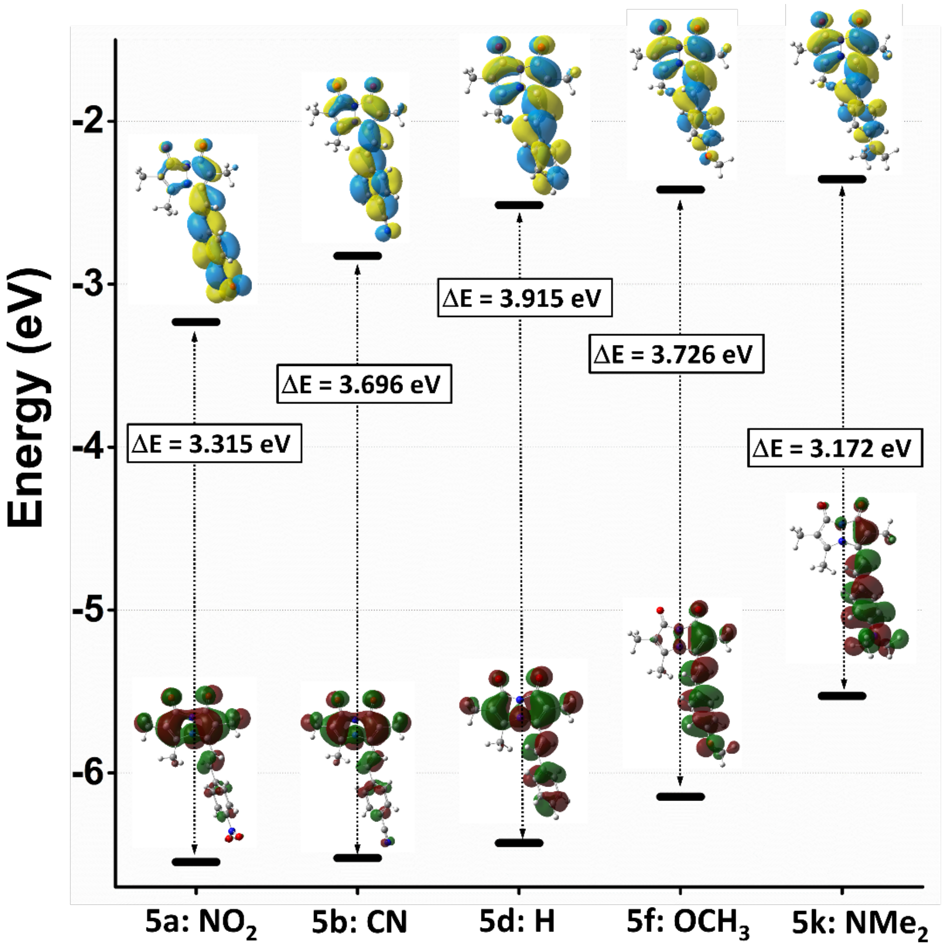

Fig. S12. Relative HOMO-LUMO energy gap (eV) of selected bimane derivatives.

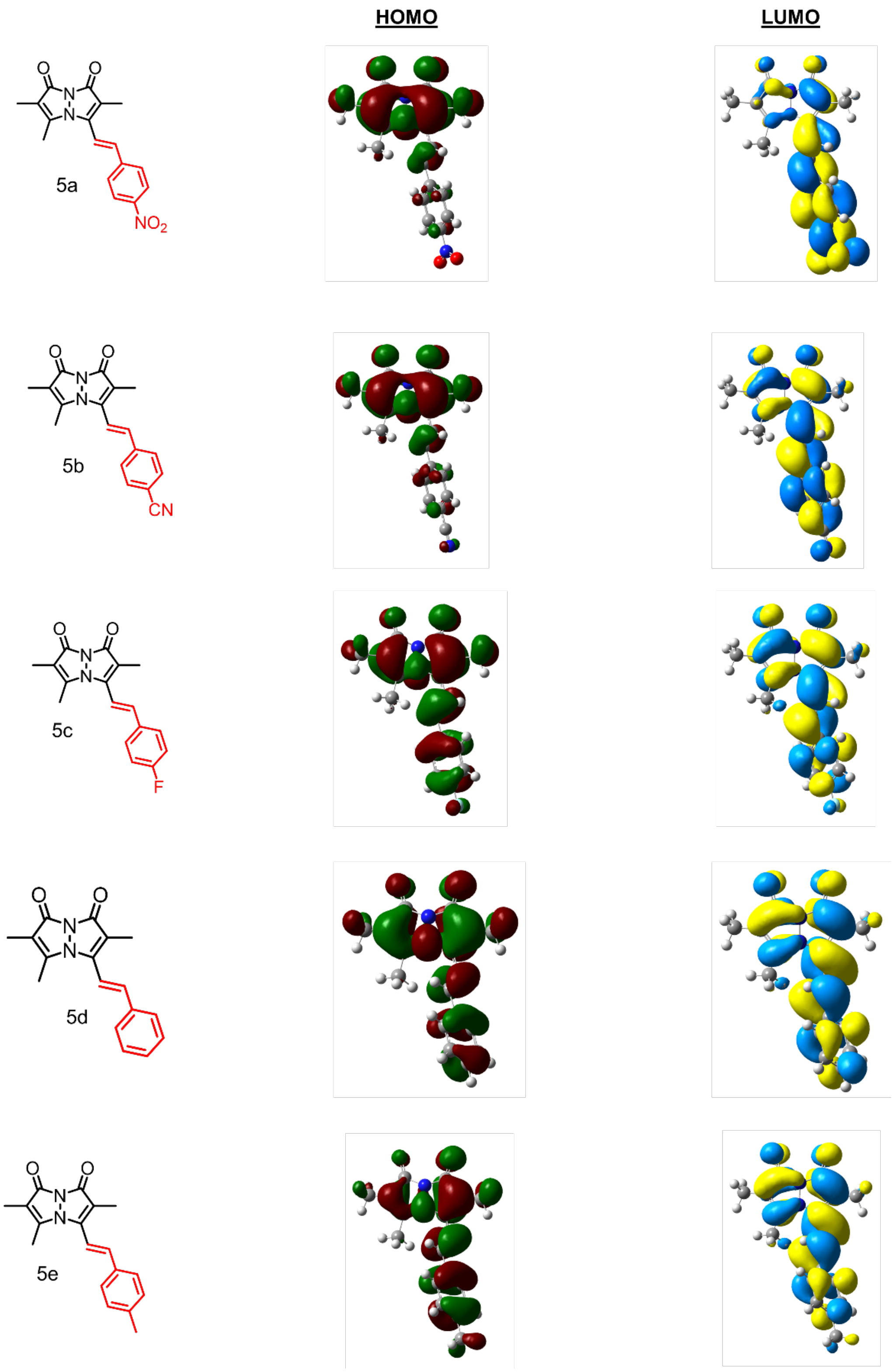

Fig. S13. HOMO and LUMO MOs of **5a-5e**.

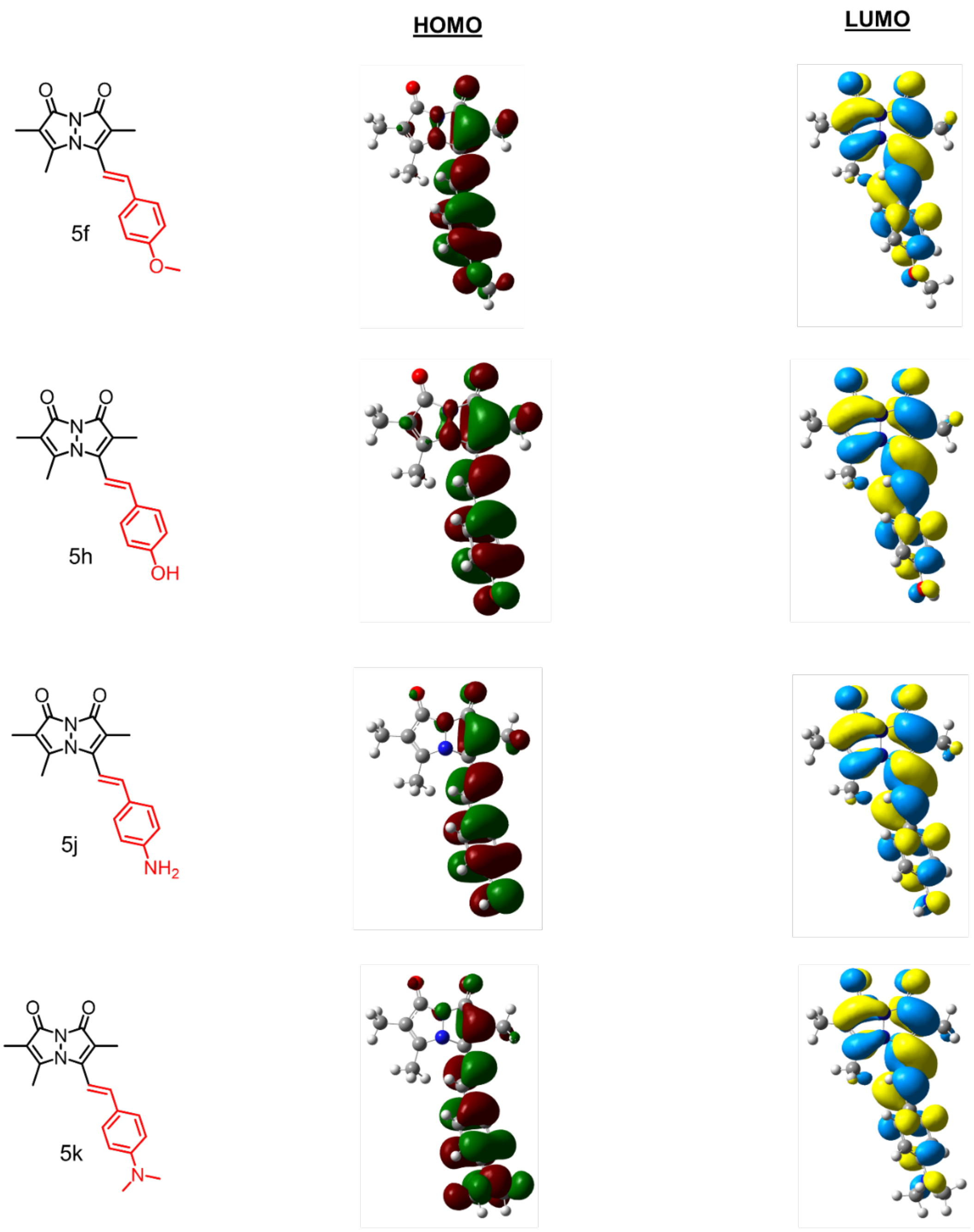

Fig. S14. HOMO and LUMO MOs of **5f, 5h, 5j,** and **5k.**

Production of α-Synuclein and 1N4R Tau

α-Synuclein production. α-Synuclein (αS) was expressed and purified as previously described with slight modifications.[4] Human αS with a C-terminal intein-His_6 fusion was transformed into *Escherichia coli* (*E. coli*) BL21 cells and plated on ampicillin plates (100 µg/mL). A single colony was then inoculated into a 5 ml primary culture containing ampicillin (100 µg/mL) in Luria-Bertain (LB) media and grown for 5-6 h with shaking (250 rpm) at 37 °C. The primary culture was then transferred to 1 L LB containing ampicillin (100 µg/mL) and grown as previously described until reaching an optical density (OD_{600}) of 0.8-1.0. At this stage, protein production was induced by the addition of isopropyl β-D-1-thiogalactopyranoside (IPTG) to 1 mM final concentration, and the culture was grown with shaking (250 rpm) overnight at 18 °C. The following day, cells were harvested by centrifugation at 4 °C for 20 min at 4000 rpm (Sorvall GS3 rotor). Pellets were then re-suspended in 20 mL/L culture of 40 mM Tris, pH 8.3 supplemented with EDTA-free protease inhibitor tablets (Pierce Biotechnology; Waltham, MA, USA) and transferred to a metal cup for sonication. Cells were lysed by sonication on ice with a Q700 probe sonicator (QSonica LLC; Newtown, CT, USA) with the following settings: Amplitude 50, Process Time 2-3 min, Pulse-ON Time 1 s, Pulse-OFF Time 1 s. Crude lysate was then transferred to 50 mL centrifugation tubes and clarified via centrifugation at 14,000 rpm for 45 min (Sorvall SS34 rotor). Following centrifugation, supernatant was removed and transferred to a 50 mL Falcon tube. Then, 5 ml of nickel agarose resin (GoldBio; St. Louis, MO, USA) was added, and the lysate-nickel mixture was incubated with nutation at 4 °C for 1 h. The lysate-nickel mixture was then poured into a 20 mL fritted column, and the flowthrough was saved. The remaining resin was then washed with ~20 mL Wash Buffer 1 (50 mM HEPES buffer, pH 7.5), ~20 mL Wash Buffer 2 (50 mM HEPES, 5 mM imidazole, pH 7.5), and eluted with 12 mL Elution Buffer (50 mM HEPES, 300mM imidazole, pH 7.5). Then 2-mercaptoethanol (Bio-Rad Laboratories; Hercules, CA, USA) was added to crude lysate (200 mM final concentration), and the mixture was allowed to incubate with nutation at room temperature overnight. The resulting cleaved protein was dialyzed against 20 mM Tris pH 8.0 for 8-10 h. The resulting dialysate was then treated with 5 mL nickel agarose resin and incubated with nutation at 4 °C for 1-2 h. The mixture was then applied to a 20 mL fritted column and flowthrough containing αS was collected in a 15 mL Falcon tube. The resulting enriched protein mixture was then dialyzed against 20 mM Tris, pH 8.0 overnight and purified via

FPLC using a 5 mL HiTrap Q-HP column (Cytiva; Marlborough, MA, USA) using the following method: Buffer A: 20 mM Tris, pH 8.0; Buffer B: 20 mM Tris, 1 M NaCl, pH 8.0; Gradient: 0% Buffer B – 5 column volumes, 0 – 10% Buffer B – 5 column volumes, 20-30% Buffer B – 20 column volumes, 30-100% Buffer B – 10 column volumes; flow rate 3 mL/min. The resulting fractions were then assessed for purity via MALDI MS, and pure fractions were combined. Protein was then concentrated, and buffer exchanged into PBS (NaCl 137 mM, KCl 2.7 mM, Na_2HPO_4 10 mM, KH_2PO_4 1.8 mM) to a final concentration of 100-200 µM via Amicon 3 kDa MWCO filters (Millipore Sigma; St. Louis, MO, USA). Purified protein was aliquoted into 1.5 mL tubes and stored at -80 °C until further use. MALDI MS $[M+H]^+$ calcd: 14460, found: 14457.

Tau 1N4R protein production: Tau (1N4R) (Tau) was expressed and purified as previously described, with slight modifications.[5] Using a fresh transformation of Tau 1N4R plasmid transformed into *E. coli* BL21 cells and plated on ampicillin plates (100 µg/mL), single colony was inoculated into a 5 mL primary culture containing ampicillin (100 µg/ml) in LB media and grown for 5-6 h with shaking (250 rpm) at 37 °C. The primary culture was then transferred to 1 L LB containing ampicillin (100 µg/ml) and grown until reaching an OD_{600} of 0.4-0.6. At this stage, protein production was induced by the addition of IPTG to 1 mM final concentration, and the culture was grown with shaking (250 rpm) overnight at 16 °C. The following day, cells were harvested by centrifugation at 4 °C for 20 min at 4000 rpm (Sorvall GS3 rotor). Pellets were then re-suspended in 15 mL Ni-NTA Buffer A (50 mM Tris pH 8, 500 mM NaCl, and 10 mM Imidazole) with 1mg/mL (chicken egg white) lysozyme, 1 tablet of EDTA-free protease inhibitor and 1 mM PMSF. The suspension was sonicated on ice for 1 minute 40 seconds, 1 second on/2 seconds off, power set to 50 W. Cellular debris was then transferred to 50 mL centrifugation tubes and clarified via centrifugation at 20,000 x g for 30 min (Sorvall SS34 rotor). Following centrifugation, supernatant was removed and filtered with a 0.22 µm syringe filter into a 50 mL Falcon tube. Then, 5 – 7 mL of nickel agarose resin was prepared and equilibrated with ~30 mL of Ni Buffer A. Then supernatant-nickel mixture was incubated with nutation at 4 °C for 1 h. Supernatant-nickel mixture was then poured into a 20 mL fritted column, and the flowthrough was saved. The remaining resin was then washed with ~30 mL Ni-NTA Buffer A and eluted with ~15 mL Ni-NTA Buffer B (50 mM Tris pH 8, 500 mM NaCl, and 400 mM imidazole). The nickel

column was cleaned by running more Ni-NTA Buffer B through the column and then re-equilibrated with Ni-NTA Buffer A. The elution was then concentrated to ~1 mL in an Amicon concentrator (10 kDa MWCO) and the following were added 25 µM TEV, and 1 mM DTT from fresh 1 M DTT stock. The mixture was allowed to incubate with nutation at 4 °C overnight and then the buffer was exchanged back to Ni-NTA Buffer A (2 cycles of 15 mL) using an Amicon concentrator. The resulting dialysate was then treated with 5 – 7 mL nickel agarose resin and incubated with nutation at 4 °C for 1 h. The mixture was then applied to a 20 mL fritted column and flowthrough containing 1N4R tau was collected in a 15 mL Falcon tube. The resulting enriched protein mixture was then concentrated, and buffer exchanged two times into Ni-NTA Buffer C (25 mM Tris pH 8, 100 mM NaCl, 1 mM EDTA, and 1 mM TCEP) using an Amicon concentrator and concentrated down to 1 mL before filtering the solution using a 0.22µm filter. Then, the protein was purified via FPLC using a size exclusion column (S200, HiLoadc16/60cSuperdex 200 pg) using the following method: isocratic elution: 100 % Ni-NTA Buffer C, – 1.2 column volumes with flowrate 0.5 mL/min. The purity of the fractions was checked by SDS-PAGE gel and the clean fractions were combined, concentrated, and the buffer exchanged into PBS to a final concentration of 100-200 µM via Amicon 10 kDa MWCO filters and stored at -80 °C until further use. MALDI MS $[M+H]^+$ calcd: 43111, found: 43109.

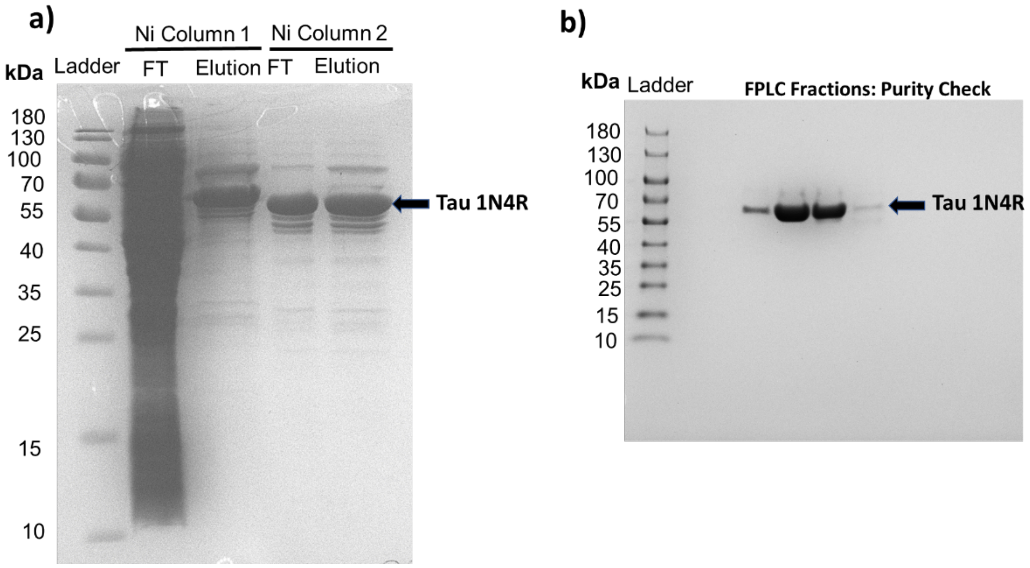

Fig. S15. Protein purification and cleavage of 1N4R tau. (a) nickel purification visualized by a Coomassie stained 10% SDS-Page gel and (b) FPLC purification fractions by SDS-PAGE gel.

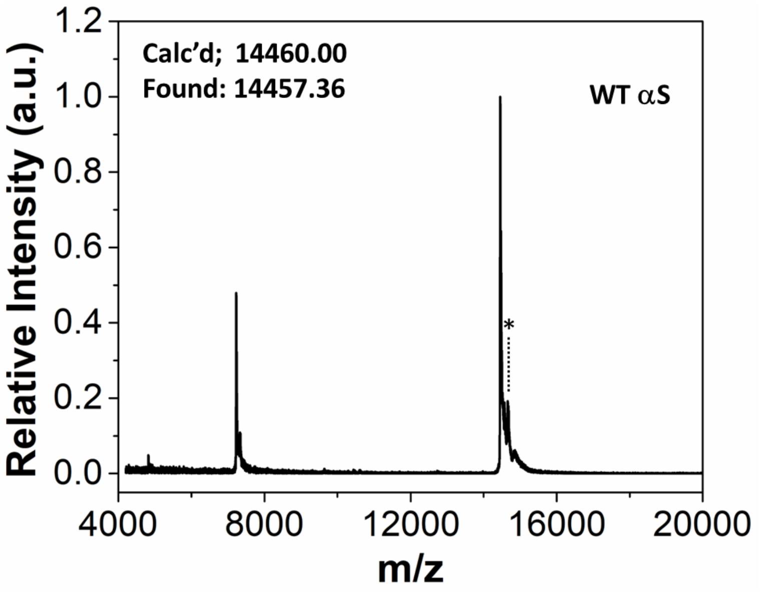

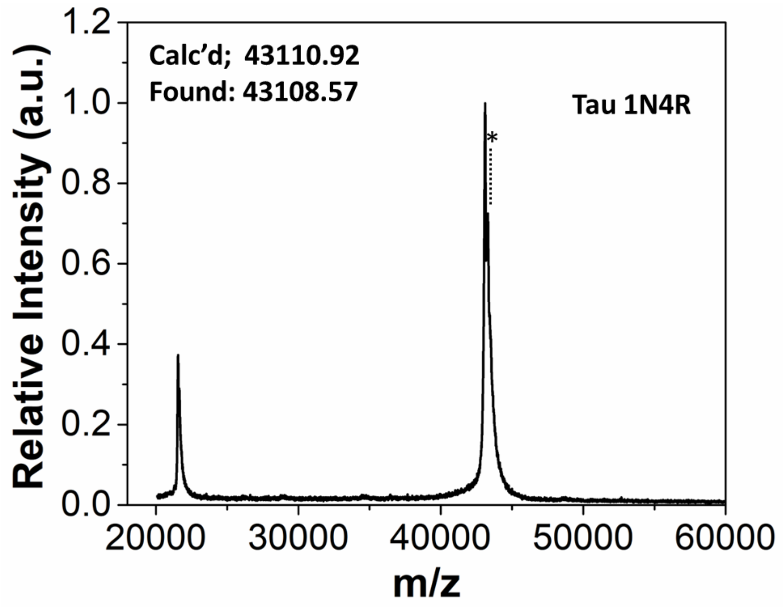

Fig. S16. MALDI MS characterization of WT αS (Top) and tau 1N4R (Bottom). On each plot matrix adduct peaks are marked with *.

Fibril Preparation

αS fibril preparation. 100 μM final concentration of αS monomer in PBS buffer (500 μL, 1.5 mL tube) at pH 7 was sealed with Teflon tape followed by parafilm and then incubated at 37 ˚C with shaking at 1300 rpm for 7 days in an IKA MS3 control orbital shaker (Wilmington, NC, USA) to get αS fibrils.[4]

1N4R Tau fibril preparation. 50 μM final concentration of 1N4R tau monomer in PBS buffer (200 μL) at pH 7.4 along with 100 μM of DTT and 12.5 μM of heparin was sealed with Teflon tape followed by parafilm incubated at 37 ˚C with shaking at 1300 rpm for 72 h in an IKA MS3 control orbital shaker (Wilmington, NC, USA) to get tau fibrils.[5]

$A\beta_{1-42}$ fibril preparation. Using 1 mg of the commercially available $A\beta_{1-42}$ (β-Amyloid (1-42), human, Genscript Cat. No. RP10017) 1 mg, a 1 mM stock solution was made by adding 222 μL hexafluoroisopropanol (HFIP) directly to the vial containing lyophilized powder through the rubber septum. After the peptide completely dissolved, the septum was pierced with a syringe needle to release the vacuum. The $A\beta_{1-42}$-HFIP solution was incubated for 30 min at room temperature. Using a positive displacement pipette, 100 μL aliquots of the solution (0.45 mg) were transferred into Eppendorf tubes and the HFIP was allowed to evaporate in the open tubes in a fume hood. The tubes were then dried on a vacuum centrifuge for 1 h without heating to remove any remaining trace amount of HFIP. To make a 1 mM stock solution of $A\beta_{1-42}$ fibrils in 10 mM Phosphate buffer at pH 7.4, 0.45 mg of $A\beta_{1-42}$ was dissolved in 10 μL DMSO with addition of 10 μL of 10 mM of NaOH and sealed in an Eppendorf tube with Teflon tape followed by parafilm, then incubated at 37 ˚C with shaking at 500 rpm for 5 days in an IKA MS3 control orbital shaker (Wilmington, NC, USA).[6,7]

Gel-based quantification of protein in fibrils. Following completion of aggregation, fibrils were pelleted by centrifugation in a bench top centrifuge at 13,200 rpm for 90 min at 4 °C. The supernatant was then carefully removed, and the fibril pellet resuspended in aggregation buffer with vortexing. Then 10 µL from each sample was transferred into an individual 0.6 mL Eppendorf tube and 2 µL 150 mM SDS in water was added (final concentration: 25 mM SDS). The tubes were then capped and heated to ~100 °C for 15-20 min. Then the tubes were placed on ice to cool for 5-10 min, and 3 µL 4X gel loading was added and the supernatant and pellet were run on an SDS-PAGE along with a monomer standard at a known concentration. After running the gel, the bands were quantified by ImageJ software to estimate the percentage in fibrils (pellet). This was used with the initial monomer concentration in the aggregation reactions to determine the concentration of fibrils for dye binding experiments.

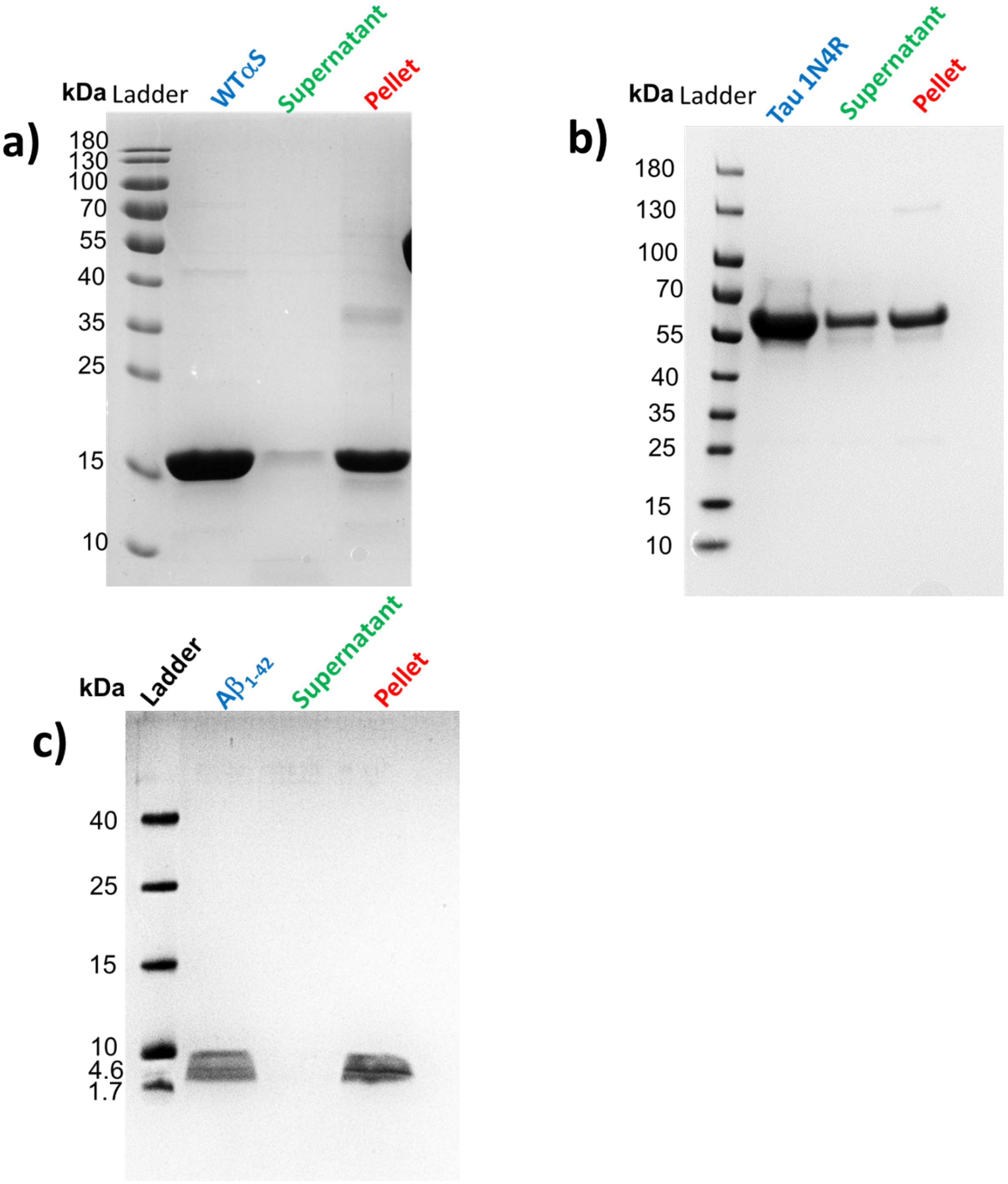

Fig. S17. Gel-based quantification of protein in αS (a), 1N4R tau (b), and A$\beta_{1\text{-}42}$ (c) fibrils.

Fibril Binding Characterization

Probe binding (K_d) measurements with αS fibrils. αS fibrils (100 μM) in PBS buffer were incubated with varying **5k** concentrations (0, 0.01, 0.1, 0.5, 1, 3, 5, 7, and 10 μM) from concentrated 10 mM DMSO stock solutions in Greiner 96 well flat black ½ area plates at 37 ˚C with shaking at 500 rpm for 15 min in an IKA MS3 control orbital shaker (Wilmington, NC, USA). After 15 min incubation, fluorescence intensity measurements were obtained with a Tecan Spark plate reader (Männedorf, Switzerland) by excitation with $\lambda_{ex}/\lambda_{em}$ = 463/580 nm for **5k**, using the following parameters: excitation and emission bandwidth 5 nm, delay time 0 μs, integration time 40 μs. Averages and standard deviations were calculated from at least 3 independent measurements at each probe concentration. The resulting binding curve was fit to the following equation (modified from the One site -- Total, accounting for ligand depletion model) in Graphpad Prism 9 (San Diego, CA, USA), from which the K_d was determined.

$$Y = \frac{a}{2}\left(b - (b^2 - 4c)^{\frac{1}{2}}\right)$$

$$b = R_t + X + K_d \qquad \text{and} \qquad C = R_t X$$

X = total probe added in μM, Y: measured fluorescence emission, K_d: Dissociation constant in μM, a: fluorescence counts/μM probe bound, and R_t = maximum probe occupancy (set to 3.3 μM based on B_{max} observed in prior radioligand binding studies).[7] We note that variation of R_t from 1 to 10 does not significantly change the fitted K_d value (less than 2-fold change).

Absorbance and Fluorescence Measurements with αS Fibrils

Fluorescence spectra with αS fibrils. Fluorescence measurements were performed using a PTI QuantaMaster™ 40 fluorescence spectrometer. Fluorescence spectra of 10 μM probes (concentrated stock solution prepared as 10 mM in DMSO) (**5b-5f** and **5h**) in PBS buffer were measured by excitation at their absorbance maximum wavelength in the absence and presence of 50 μM αS fibrils (stock solution 100 μM αS fibrils in PBS buffer).

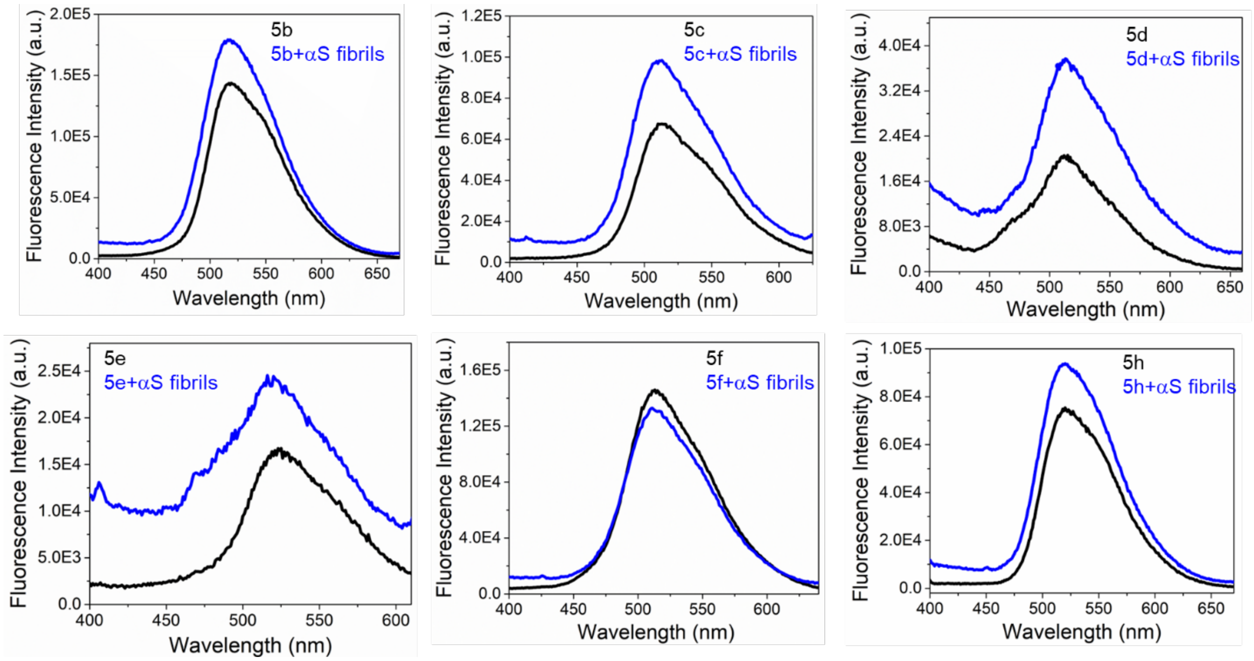

Fig. S18. Fluorescence spectra of probes (**5b-5f** and **5h**) with and without αS fibrils. Final concentrations of probes and αS fibrils were 10 μM and 50 μM, respectively. Emission spectra collected at each dye's maximum absorption wavelength. Ex/Em slit widths: 3 nm/3 nm.

Absorbance spectra with αS fibrils. Absorption measurements were performed using Thermo Scientific Genesys 150 UV Vis spectrometer. Absorption spectra of 10 μM probes (concentrated stock solution prepared as 10 mM in DMSO) (5j and 5k) in PBS buffer were measured in the absence and presence of 50 μM αS fibrils (stock solution 100 μM αS fibrils in PBS buffer).

Excitation spectra with αS fibrils. Excitation spectral measurements were performed using a PTI QuantaMaster™ 40 fluorescence spectrometer. Excitation spectra of 10 μM probes (concentrated stock solution prepared as 10 mM in DMSO) (5j and 5k) in PBS buffer were measured in the absence and presence of 50 μM αS fibrils (stock solution 100 μM αS fibrils in PBS buffer).

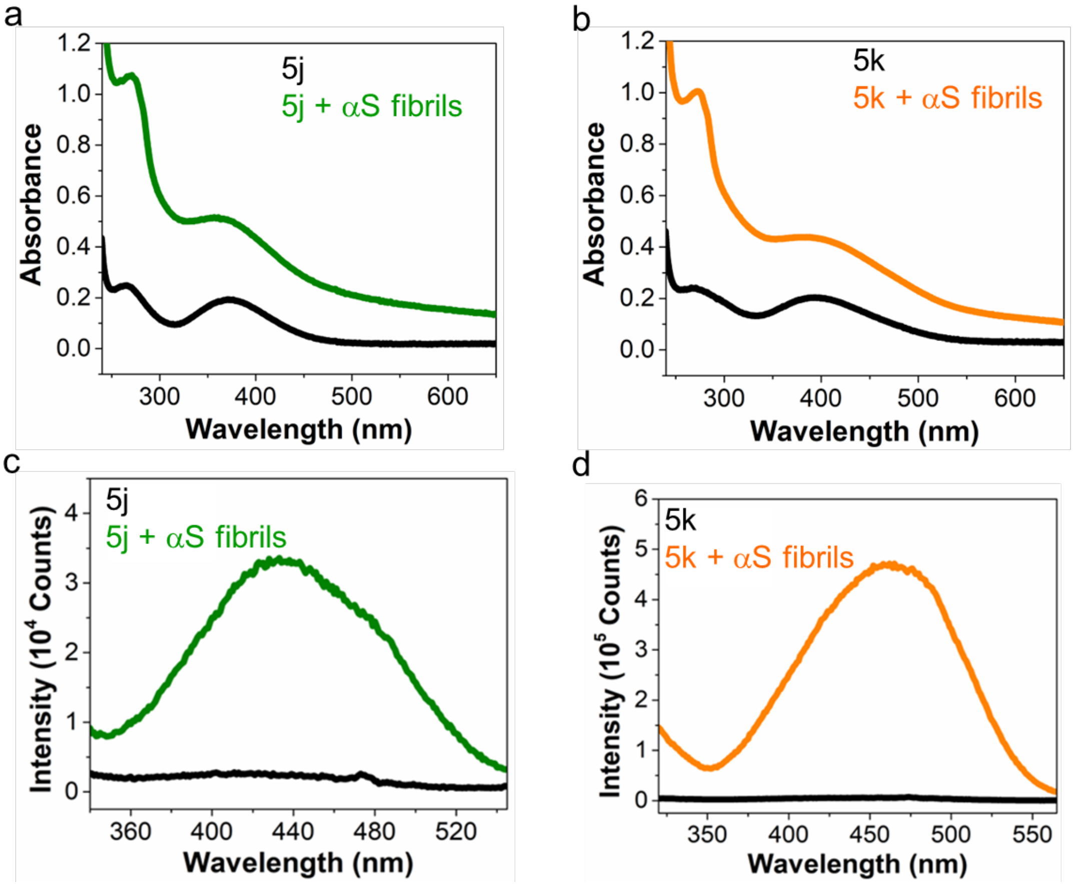

Fig. S19. Absorbance (a, b) and excitation spectra (c, d) of probes **5j** and **5k** with and without αS fibrils. Final probe and fibrils concentrations were 10 μM and 50 μM, respectively. Final probe and fibril concentrations were 10 μM and 50 μM, respectively. Emission wavelengths were 575 nm for **5j**, 580 nm for **5k**. Ex/Em slit widths: 3 nm/3 nm.

Fluorescent QY measurements of 5j and 5k with αS fibrils. For each QY measurement, the incident excitation light spectrum was collected with 100 μL of probes (100 μM) in PBS buffer. After measuring the incident light intensity, probes (**5j and 5k**) were added to the αS fibrils (100 μM) from a concentrated stock solution (10 mM in DMSO) to a final concentration of 100 μM and the new spectrum (fluorescence intensity and new incident light intensity) was collected. Using the JASCO Quantum Yield Software, the dye QY was calculated by dividing the dye fluorescence intensity by the difference in incident light intensity in the presence and absence of fibrils.

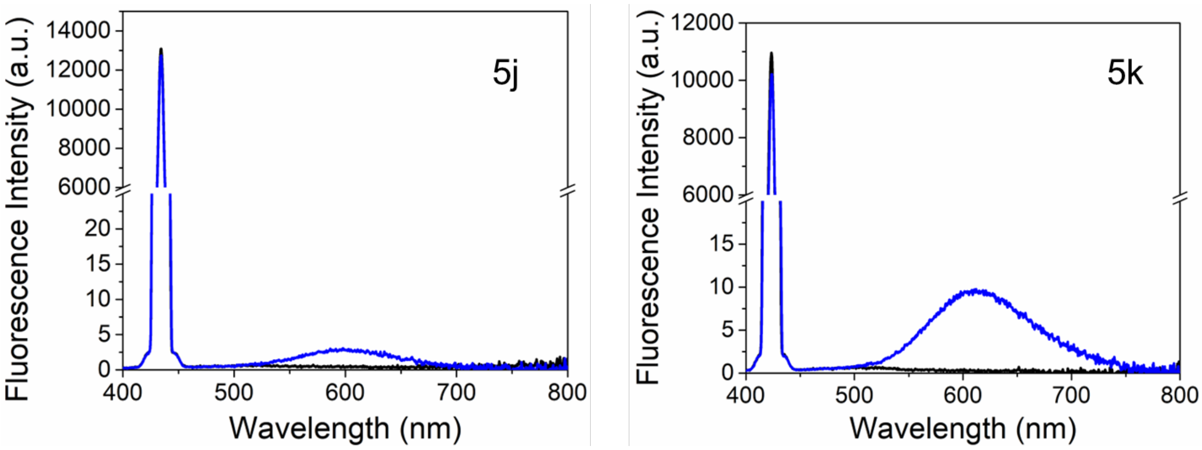

Fig. S20. Representative QY acquisitions of **5j** and **5k** with αS fibrils. Black line indicates incident light (αS fibrils without probe) and blue line indicates sample (αS fibrils with probe). Excitation at λ_{exc} = 435 nm for **5j** and λ_{exc} = 463 nm for **5k** and spectral collection (400-800 nm). Final concentrations of the probe and αS fibrils are the 100 μM. Ex/Em slit widths: 5 nm/5 nm.

Fluorescence lifetime measurements of probes with αS fibrils. TCSPC measurements of fluorescence lifetime decays for 100 μM dyes in the presence of 100 μM αS fibrils were collected with the PTI Quantamaster TM 40 using a pulsed LED with a maximum emission at 486 nm. Fluorescence emission was collected at the indicated wavelength for each dye with 20 nm slit widths. The IRF was collected under identical conditions. Data analysis was performed with FluoFit software using an exponential decay model.

Table S15. Fluorescence lifetime measurements of αS fibrils with probes **5j** and **5k** in PBS buffer and fit χ^2 values.[a]

Probe	Lifetime (ns)	χ^2
**5j**	1.90 ± 0.01	1.221
**5k**	2.27 ± 0.01	1.079

[a]Emission wavelength collected at 575 nm for **5j** and 580 nm for **5k**. Final concentrations of the probe and αS fibrils were 100 μM.

Fluorescence Measurements with Tau Fibrils

Fluorescence spectra with tau fibrils. Fluorescence measurements were performed using a PTI QuantaMaster™ 40 fluorescence spectrometer. Fluorescence spectra of 5 μM probes (concentrated stock solution prepared as 10 mM in DMSO) (5j, 5k, and ThT) in PBS buffer were measured in the absence and presence of 25 μM tau fibrils (stock solution 32 μM τau fibrils in PBS buffer).

Excitation spectra with tau fibrils. Excitation spectral measurements were performed using a PTI QuantaMaster™ 40 fluorescence spectrometer. Excitation spectra of 5 μM probes (concentrated stock solution prepared as 10 mM in DMSO) (5j, 5k, and ThT) in PBS buffer were measured in the absence and presence of 25 μM tau fibrils (stock solution 32 μM tau fibrils in PBS buffer).

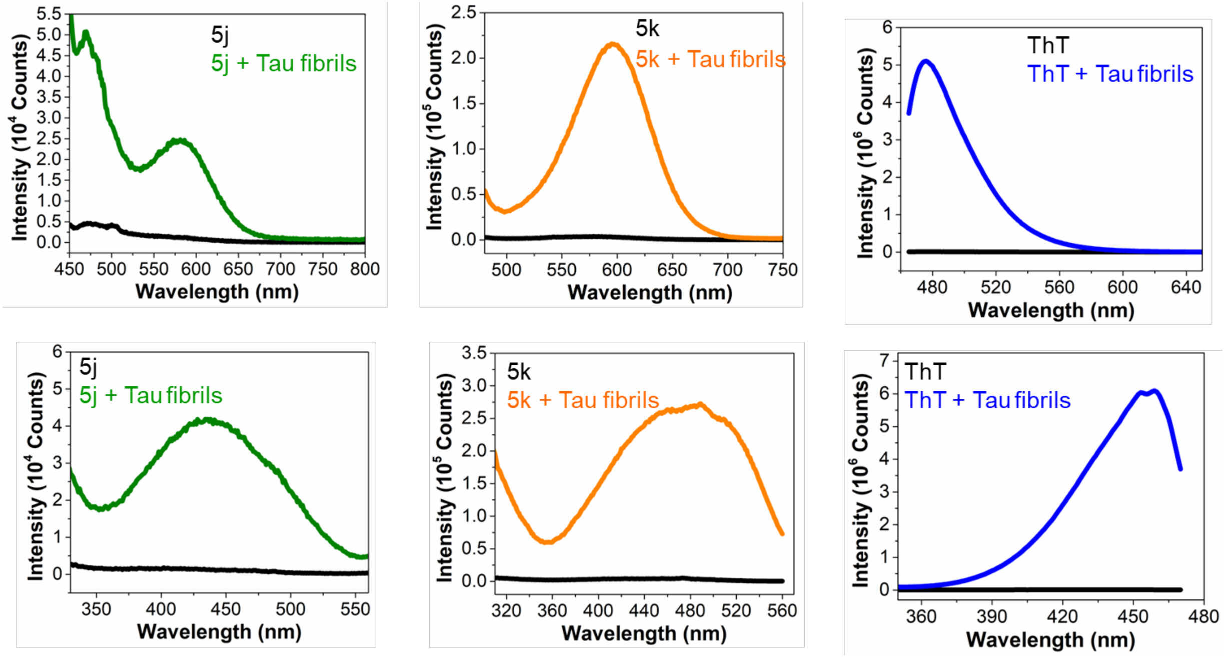

Fig. S21. Fluorescence spectra (a-c) and excitation spectra (d-f) of **5j**, **5k** and **ThT** with and without tau fibrils. Final probe and tau fibril concentrations were 5 μM and 25 μM, respectively. Excitation at λ_{exc} = 435 nm for **5j**, λ_{exc} = 463 nm for **5k**, λ_{exc} = 450 nm for **ThT**. Emission wavelengths were 575 nm for **5j**, 580 nm for **5k**, and 482 nm for ThT . Ex/Em slit widths: 3 nm/3 nm.

Fluorescence Measurements with Aβ_{1-42} Fibrils

Fluorescence spectra with Aβ_{1-42} fibrils. Fluorescence measurements were performed using a PTI QuantaMaster™ 40 fluorescence spectrometer. Fluorescence spectra of 10 µM probes (concentrated stock solution prepared as 10 mM in DMSO) (5j, 5k and ThT) in PBS buffer were measured in the absence and presence of 50 µM Aβ_{1-42} fibrils (stock solution 1 mM Aβ_{1-42} in 10 mM phosphate buffer, pH 7.4).

Excitation spectra with Aβ_{1-42} fibrils. Excitation spectral measurements were performed using a PTI QuantaMaster™ 40 fluorescence spectrometer. Excitation spectra of 10 µM probes (concentrated stock solution prepared as 10 mM in DMSO) (5j, 5k and ThT) in PBS buffer were measured in the absence and presence of 50 µM Aβ_{1-42} fibrils (stock solution 1 mM Aβ_{1-42} in phosphate buffer).

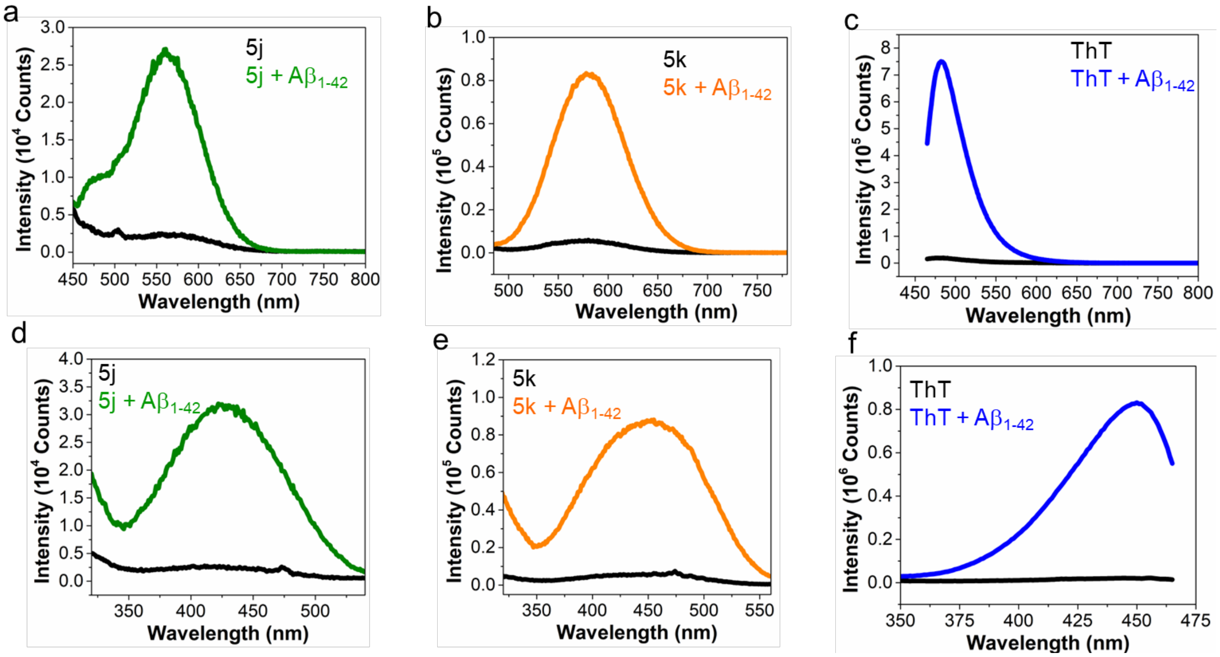

Fig. S22. Fluorescence spectra (a-c) and excitation spectra (d-f) of **5j**, **5k** and **ThT** with and without Aβ_{1-42} fibrils. Final probe and fibrils concentrations were 10 µM and 50 µM. Excitation at λ_{exc} = 435 nm for **5j**, λ_{exc} = 463 nm for **5k**, and λ_{exc} = 450 nm fot **ThT**. Emission wavelengths were 575 nm for **5j**, 580 nm for **5k**, and 482 nm for ThT. Ex/Em slit widths: 3 nm/3 nm.

HEK cell lysate preparation. HEK 293T cells were grown on 150 mm dishes in sterile-filtered Dulbecco's modified Eagle's medium (Gibco, Thermo Fisher) supplemented with 10% fetal bovine serum (FBS), 1% penicllin/streptomycin (Mediatech, 10 mg/ml each). Cells were then washed with PBS and harvested in 5 mL PBS by scraping. Cells were pelleted by centrifugation at 1000 rpm for 5 min, and the supernatant was removed. The resulting cell pellet was re-suspended in 300-500 μL PBS buffer with 0.1% Triton X-100 (Bio-Rad) and 1x HALT protease inhibitor cocktail without EDTA (Thermo Fisher), then lysed by sonication for two 30 s cycles (cycle 1: 2s on 2s off, amp 50; cycle 2: 2s on 2s off, amp 55) using a QSonica Q700 sonicator fitted with a microtip. Following sonication, lysate was centrifuged at 13,200 rpm for 60 min to separate membrane and cytosolic protein fractions. The resulting lysate cytosolic or membrane fraction was then used immediately after concentration determination by DC protein assay (Bio-Rad).

Selectivity of probe 5k binding with αS fibrils in HEK cell lysate. Probe **5k** in PBS buffer (1 μM, the stock solution prepared as 10 mM in DMSO) were incubated with different αS fibril concentrations (0, 5, 10, 25 and 50 μM) in the presence of 10 mg/mL HEK cell lysate in Greiner 96 well flat black ½ area plates at 37 ˚C with shaking at 500 rpm for 15 min in an IKA MS3 control orbital shaker. After 15 min incubation, fluorescence intensity measurements were obtained with a Tecan Spark plate reader (Männedorf, Switzerland) by excitation with $\lambda_{ex}/\lambda_{em}$ = 463/580 nm for **5k**, using the following parameters: excitation and emission bandwidth 5 nm, delay time 0 μs, integration time 40 μs.

Experimental procedure for the fluorescent measurements of 5k with TMAO: A 1 mM stock solution of 5k in DMSO and a 6.5 M stock solution of trimethylamine *N*-oxide (TMAO) in Tris buffer at pH 7.4 were prepared. From these stocks, the fluorescence measurements of probe **5k** (10 μM) were carried out with and without αS monomer (50 μM) at different concentrations of TMAO (2 M, 4 M and 6 M) diluted with the Tris buffer. The solutions were mixed and then the spectra were measured using a Photon Technology International (PTI) QuantaMaster™ 40 fluorescence spectrometer with excitation at 418 nm.

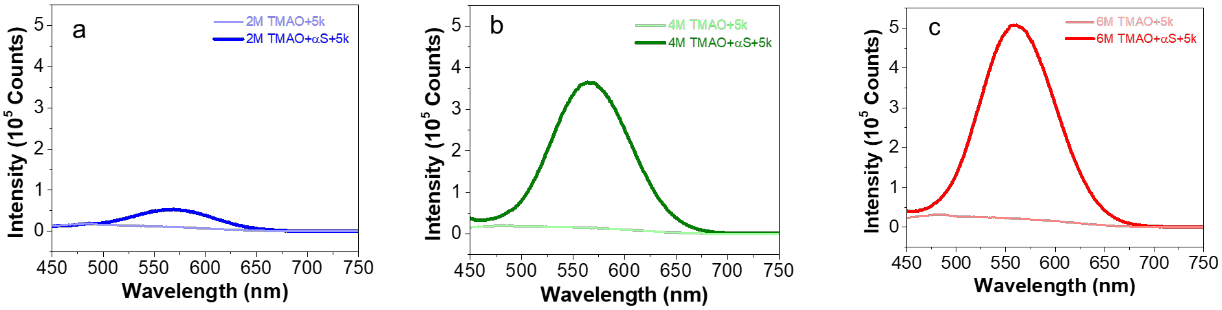

Conc. of TMAO (M)	2	4	6
λ_{max}(nm) of 5k	567	566	560

Fig. S23. Fluorescence spectra of probe **5k** (10 μM) with and without αS monomer (50 μM) in different TMAO concentrations. (a) 2 M TMAO, (b) 4 M TMAO, (c) 6 M TMAO, and tabulated λ_{max} (λ_{em}) of **5k** with increasing TMAO concentrations. Excitation wavelength for **5k** is 418 nm. Ex/Em slit widths: 3 nm/3 nm.

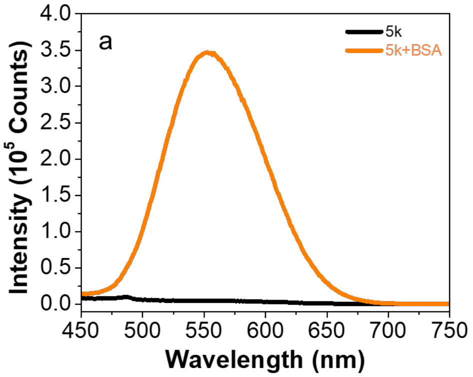

b

Conc. of BSA	50 μM
λ_{max} of 5k (10 μM)	555 nm

Fig. S24. (a) Fluorescence spectra of probe **5k** (10 μM) with BSA (50 μM) and (b) Tabulated λ_{max} (λ_{em}) of **5k**. Excitation wavelength for **5k** is 418 nm. Ex/Em slit widths: 3 nm/3 nm.

Patient Tissue Characterization and Amplified Fibril Preparation

Preparation of sarkosyl-insoluble αS from disease and control brains. All procedures were performed in accordance with local institutional review board guidelines. Written informed consent for autopsy and analysis of tissue sample data was obtained either from patients themselves or their next of kin. Frozen postmortem human frontal cortex brain tissues from three patients diagnosed with Alzheimer's disease (AD) and three patients diagnosed with Parkinson's disease with dementia (PDD) were selected for sequential extraction of αS aggregates based on a high burden of αS pathology determined by immunohistochemical staining using previously established methods.[9] In brief, 5–10 g of frontal cortical gray matter were homogenized in five volumes (W/V) of high-salt (HS) buffer (50 mM Tris– HCl pH 7.4, 750 mM NaCl, 10 mM NaF, 5 mM EDTA) with protease and protein phosphatase inhibitors, incubated on ice for 20 min and centrifuged at 100,000 ×g for 30 min. The pellets were then re-extracted with HS buffer, followed by sequential extractions with five volumes of 1% Triton X-100-containing HS buffer and 1% Triton X-100-containing HS buffer with 30% sucrose. The pellets were then re-suspended and homogenized in 1% sarkosyl-containing HS buffer, rotated at room temperature for 2 h or at 4 °C overnight and centrifuged at 100,000 ×g for 30 min. The resulting sarkosyl-insoluble pellets were washed once with Dulbecco's PBS (DPBS) and re-suspended in DPBS by sonication (QSonica Microson XL-2000; 50 pulses; setting 2; 0.5 s per pulse). These final sarkosyl-insoluble fractions are referred to as "brain extracts.: The amount of αS in the brain extracts was determined by sandwich ELISA using Syn9027, a mono-clonal antibody (Mab) to αS, and the protein concentrations were examined by bicinchoninic acid (BCA) assay as previously described.[9] Total protein in AD cases ranged from 5 to 13 mg/mL and total protein in PDD cases ranged from 9 to 12 mg/mL. αS concentration in AD cases ranged from 36 to 43 µg/mL and total protein in PDD cases ranged from 24 to 33 µg/mL.

***In vitro* amplification of αS fibrils from brain extracts.** Brain-derived αS aggregates in extract samples from three AD and three PDD cases (Fig. S23a) were used as seeds for templated aggregation of recombinant αS monomer. Brain lysates were sonicated on high for 20 cycles of 30 s on, followed by 30 s off (Diagenode Biorupter UCD-300 bath sonicator) and diluted to 350 nM (based on sandwich ELISA) in DPBS (pH 7.4, without $Mg^{2+}$and Ca^{2+}) containing sufficient monomer for a final αS concentration of 35µM (1% patient-derived seed, 99% monomer) and

incubated at 37° C with constant shaking at 1000 rpm for 7 days. The resulting aggregates were termed amplified fibrils (AFs). After seven days of incubation, amplification progress was determined by removing an aliquot and diluting tenfold in DPBS to a final αS concentration of 3.5 μM. Samples were centrifuged at 45k rpm for 30 min at 10°C in a TLA-55 rotor (Beckman Coulter). The supernatants were removed, and the pellets were resuspended in DPBS. Equal volumes of supernatant and pellet fractions were analyzed on SDS-PAGE gels and Western blot, with amplified aggregates migrating to the pellet fraction (Fig. S23b). Primary mouse hippocampal neurons were then treated with the AFs to confirm they retained the characteristic pathological phenotype of the original brain lysate and were distinct from recombinant PFFs. Primary mouse neurons were prepared from the hippocampus of embryonic day E16–E18 CD1 mouse embryos as previously described 10. All procedures were performed according to the National Institutes of Health Guide for the Care and Use of Experimental Animals and were approved by the University of Pennsylvania Institutional Animal Care and Use Committee. Primary mouse hippocampal neurons were plated at 17.5k cells per well in 96-well plates and were treated with 50 ng per well of brain-derived, AFs, or recombinant PFFs at seven days in vitro and maintained for two weeks post-treatment. Cells were grown in Neurobasal medium supplemented with B27, 2 mM GlutaMax, and 100 U/ml penicillin/ streptomycin (ThermoFisher). Following fixation with 4% paraformaldehyde, 4% sucrose, and 1% Triton-X100, cultures were probed with antibodies for MAP2 (17028) and pSer-129 αS (81A) Patient-derived fibrils and AFs induce discrete, dense inclusions in cell bodies with little neuritic pathology while PFF induce predominantly small neuritic puncta and threads (Fig. S24).

Control sample preparation. Control samples for comparison to AFs in fluorescence binding experiments below were prepared as follows. "Lysate" controls used a portion of the same brain extracts from AD cases 1-3 and PDD cases 1-3, but were not subjected to amplification. These should contain the same concentration of patient-derived αS and other insoluble proteins as the AF samples, but no seeded fibrils. Pre-formed fibril (PFF) controls took these lysates and added αS fibrils, generated as described above for binding studies of the pure fibrils, to match the αS monomer concentration used in generating AFs (35 μM). Thus, they would have the same fibril content as an amplification reaction which went to completion, but with a fibril morphology that is not templated by the patient-derived αS aggregates.

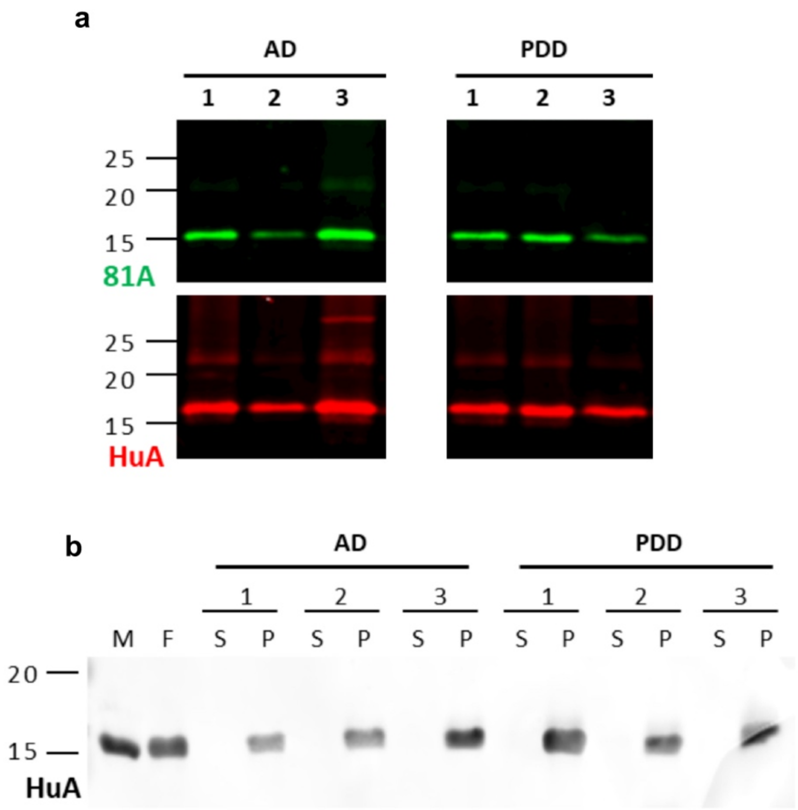

Fig. S25. Preparation of fibrils amplified from patient-derived α-synuclein aggregates. (a) Western blots of the final brain lysate samples extracted from AD and PDD patient brains. Membranes were probed with anti-pSer-129 αS (81A) or total αS (HuA). (b) Western blot of sedimentation results for amplified fibrils (AFs) after seven days of incubation. Supernatant (S) and pellet (P) fractions were run for each reaction with monomer (M) and PFF (F) as loading controls. The membrane was probed with HuA.

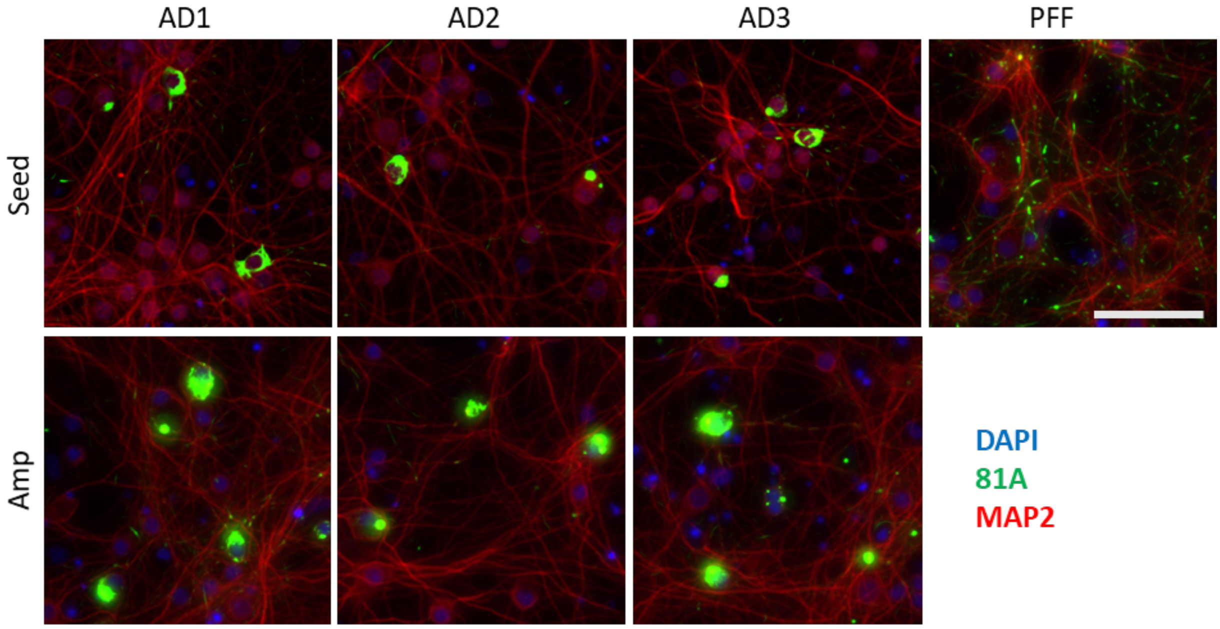

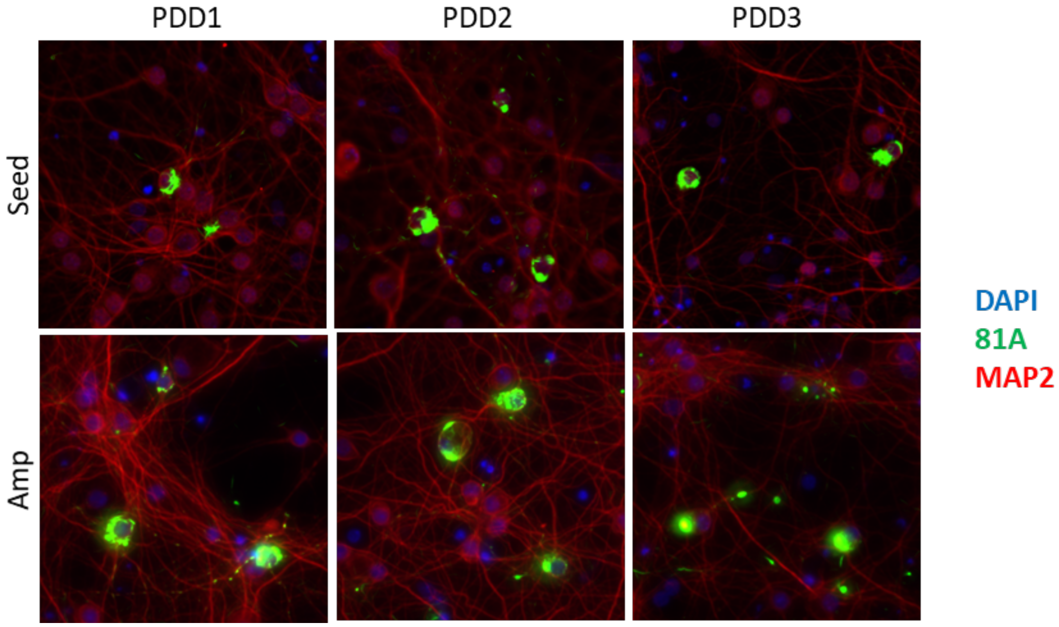

Fig. S26. Representative images of pathology induced in primary mouse hippocampal neurons for seeds and AFs (Amp) for each case, or PFFs. Probed with 81A (green), anti-MAP2 (red) and DAPI (blue). Scale bar 50 µm.

Fluorescence Measurements with Patient Tissue Lysate Samples

Fluorescence measurements of 5k and **ThT with AD and PDD patient cases.** Probe binding was tested with 7 μM stock concentrations of patient tissue samples from 3 different AD patient cases (AD1, AD2, and AD3) with 3 different conditions: lysate only, amplified αS fibrils (AFs), preformed αS fibrils (PFFs), all prepared as described above. Final samples were prepared in a Greiner 384–well small volume microplate by diluting to 1 μM final concentration with PBS buffer from each condition's stock in a 5 μL final volume. To this was added bimane probes **5k** and **ThT** at 1 μM probe concentration (10 mM stock of **5k** and **ThT** in DMSO and 5 mM stock of ThT in PBS buffer), then incubated at 37 ˚C with shaking at 500 rpm for 15 min in an IKA MS3 control orbital shaker. After 15 min of incubation, fluorescence intensity measurements were obtained with a Tecan Spark plate reader by excitation $\lambda_{ex}/\lambda_{em}$ = 463/580 nm for **5k** and $\lambda_{ex}/\lambda_{em}$ = 450/482 nm for **ThT**, using the following parameters: excitation and emission bandwidth 5 nm, delay time 0 μs, integration time 40 μs. Identical experiments were performed for 7 μM stock concentrations of patient tissue samples from 3 different PDD patient cases (PDD1, PDD2, and PDD3) with 3 different conditions: lysate only, amplified αS fibrils (AFs), preformed αS fibrils (PFFs).

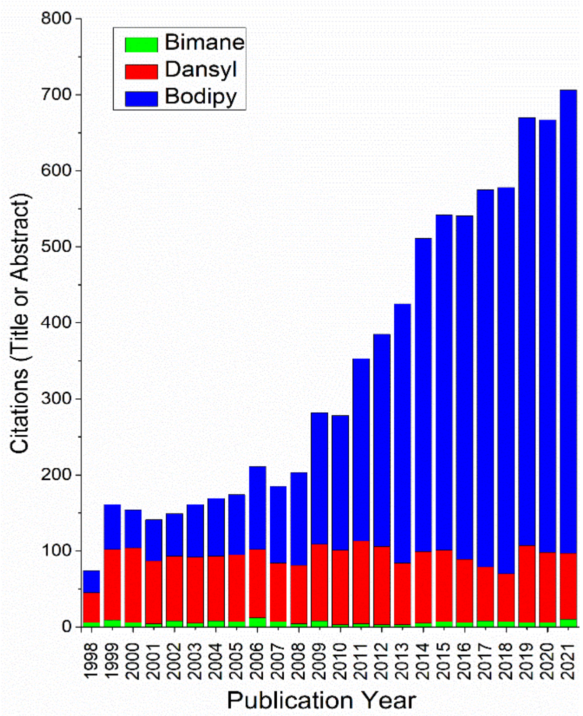

Fig. S27. Analysis of bimane literature relative to similar fluorophores such as BODIPY and Danysl fluorophores (https://www.webofscience.com/wos/woscc/basic-search).

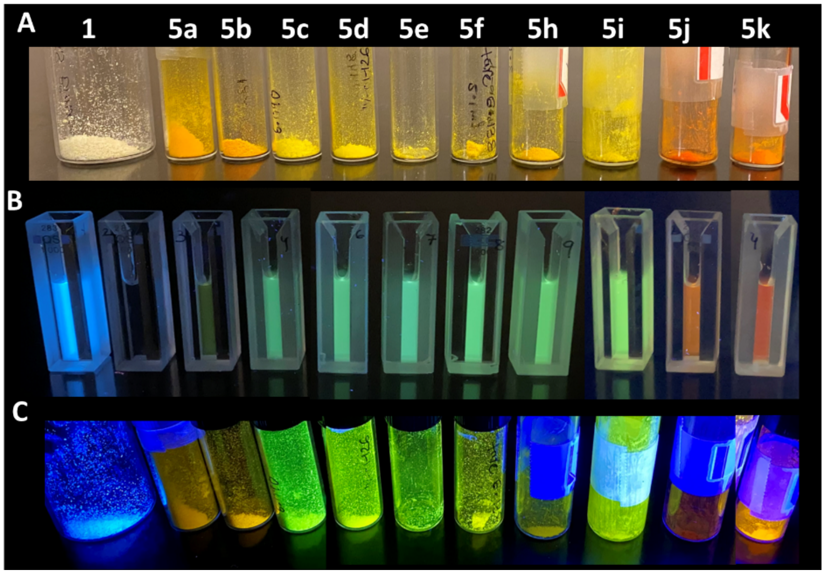

Fig. S28. Photophysical properties of **1** and **5a-f**, **5h-k**. (A) Photograph under ambient light, (B) Solution state fluorescence of 25 μM probe in ACN/PBS buffer (50:50 v/v) under handheld UV lamp (365 nm), and (C) Amorphous solid-state fluorescence under handheld UV lamp (365 nm).

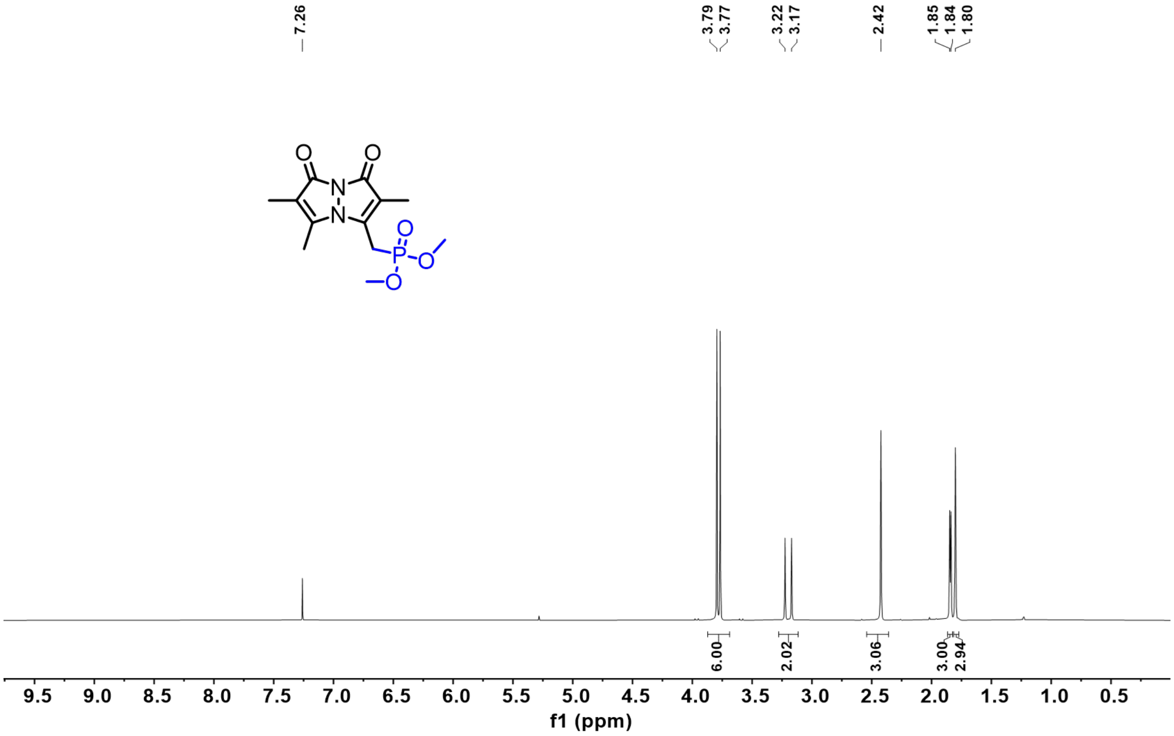

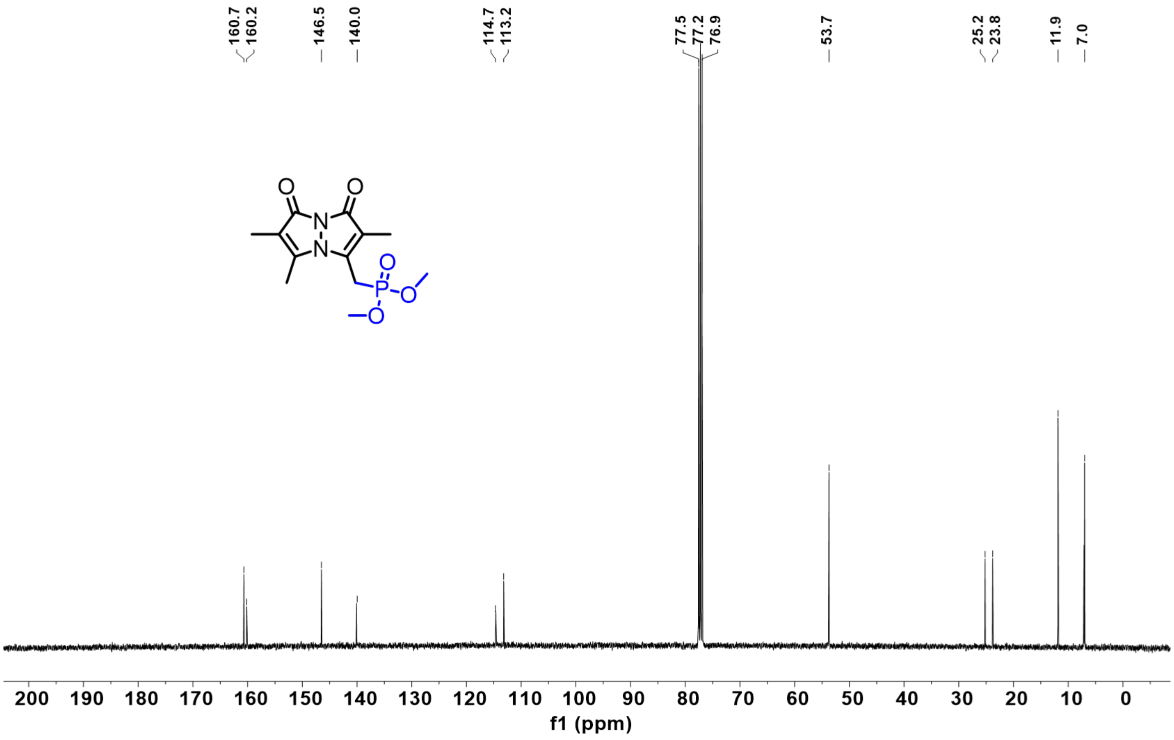

Fig. S29. ^{1}H NMR and ^{13}C NMR spectrum of **3** in $CDCl_3$.

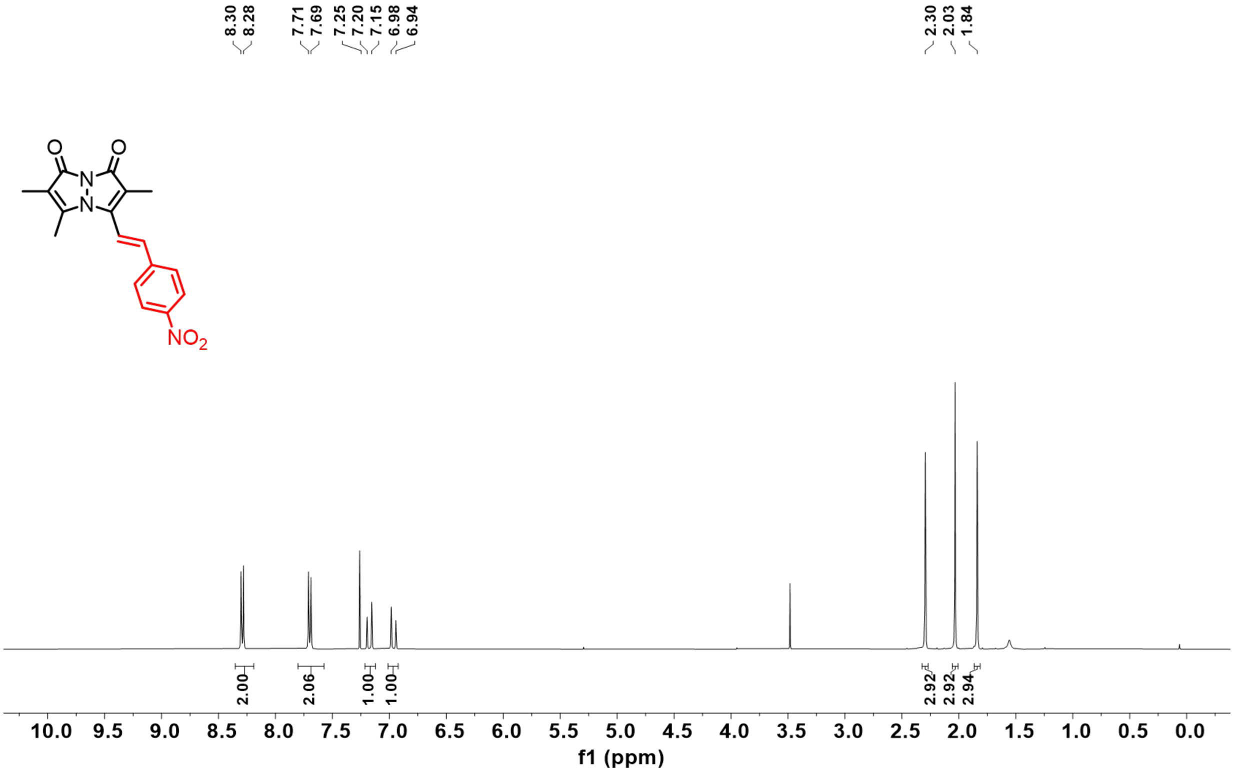

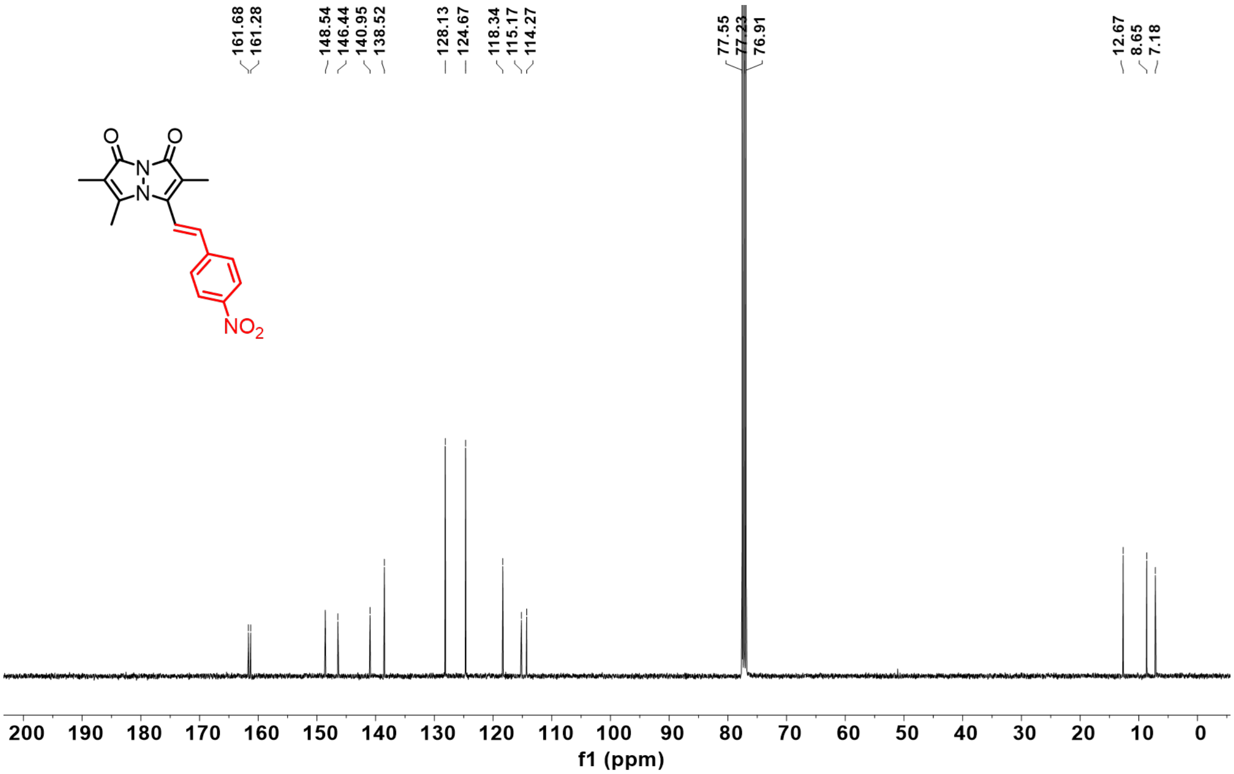

Fig. S30. ^{1}H NMR and ^{13}C NMR spectrum of **5a** in $CDCl_3$.

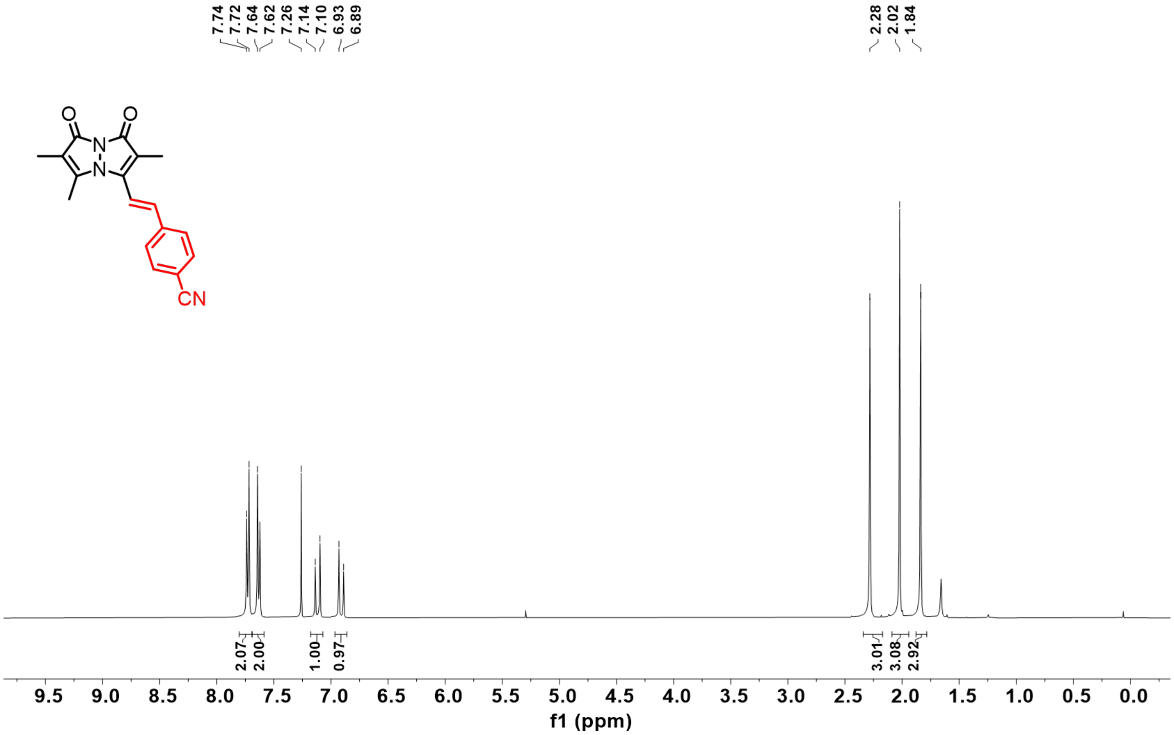

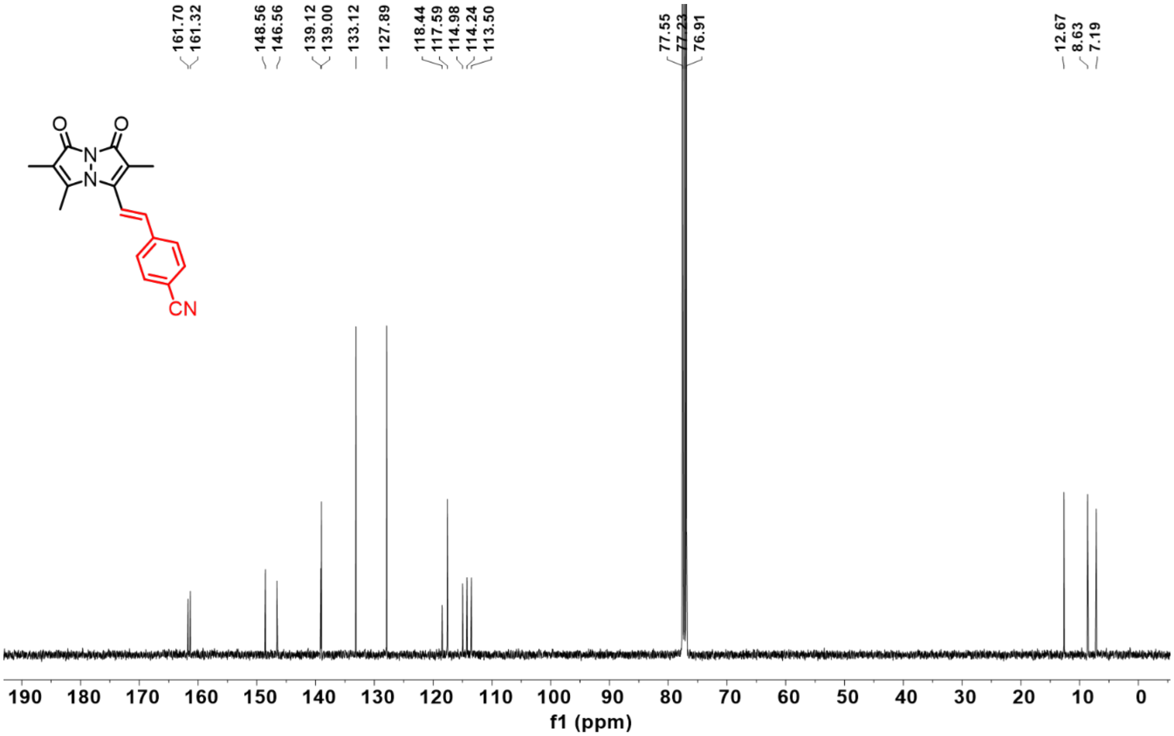

Fig. S31. ^{1}H NMR and ^{13}C NMR spectrum of **5b** in $CDCl_3$.

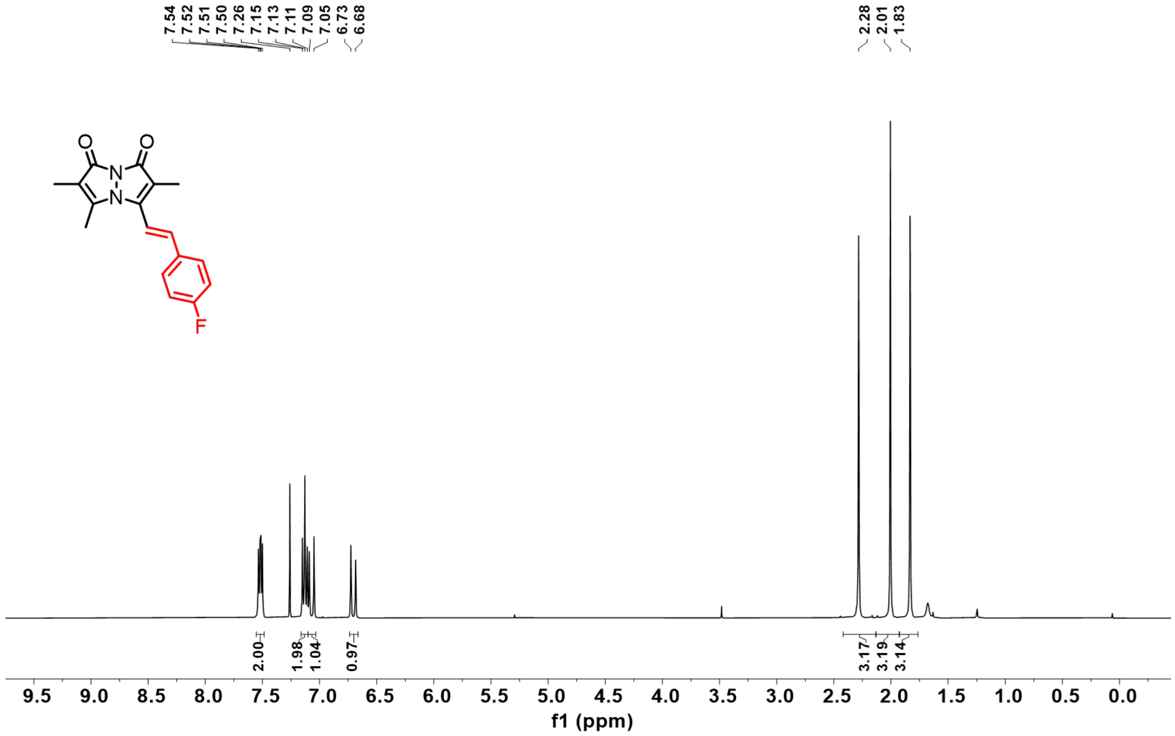

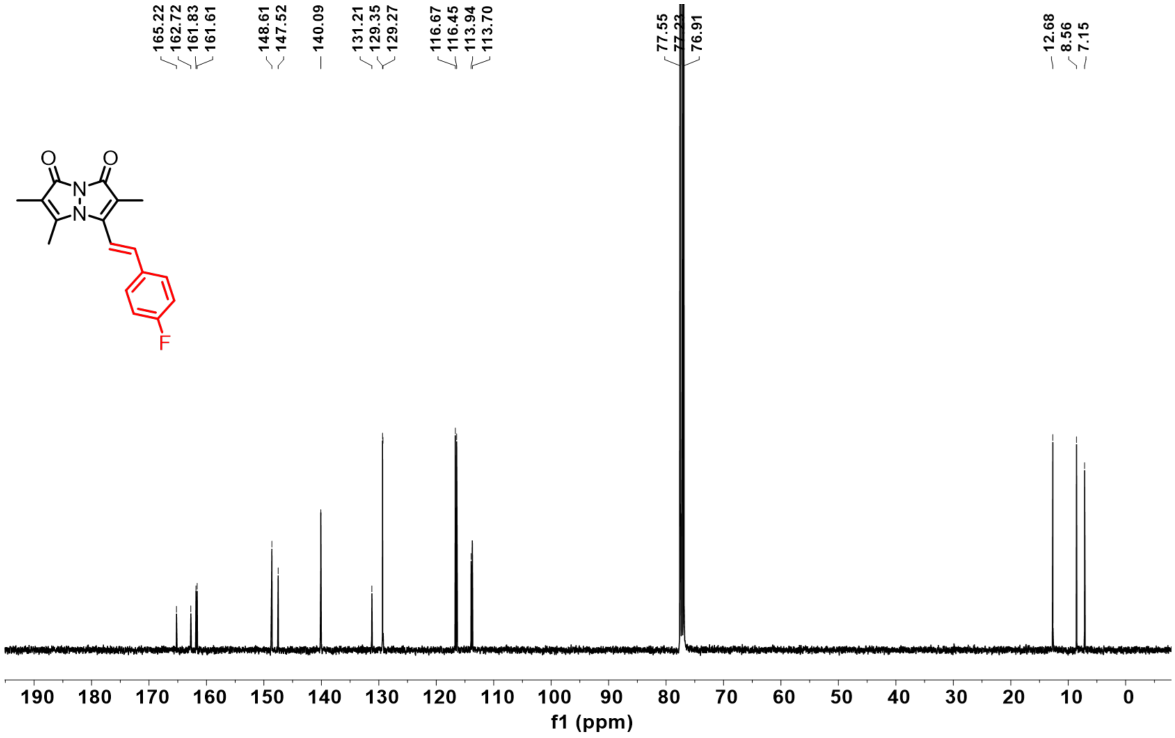

Fig. S32. ^{1}H NMR and ^{13}C NMR spectrum of **5c** in $CDCl_3$.

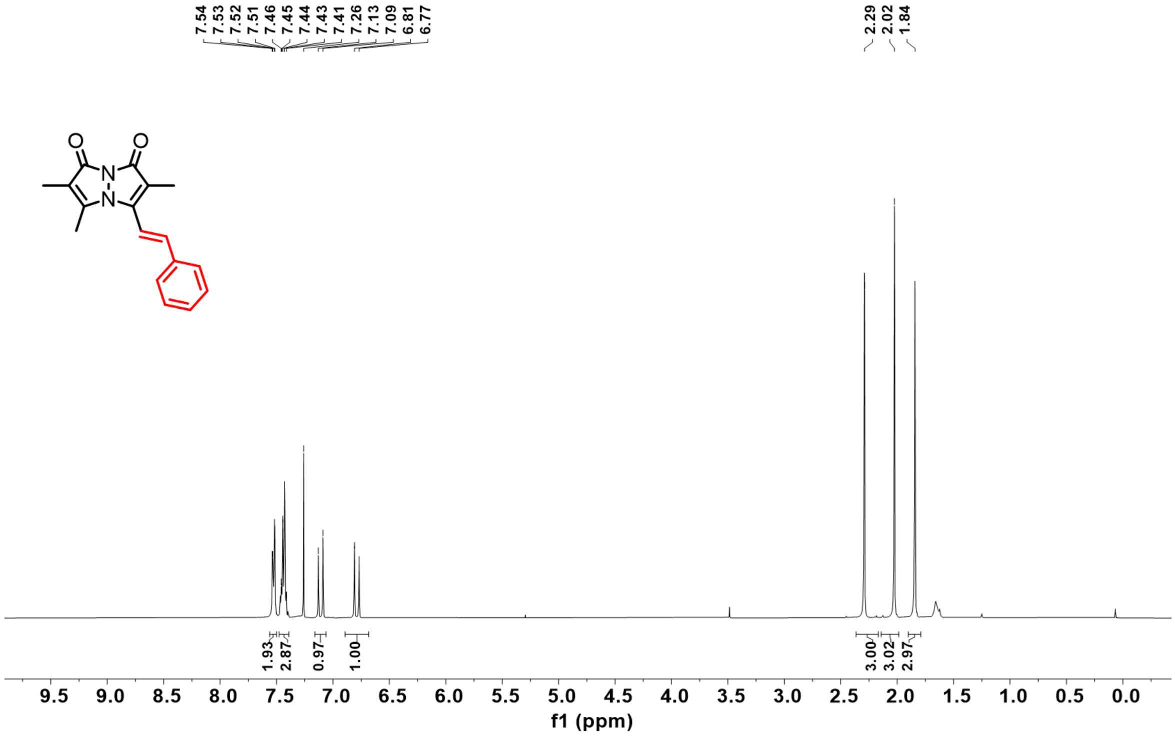

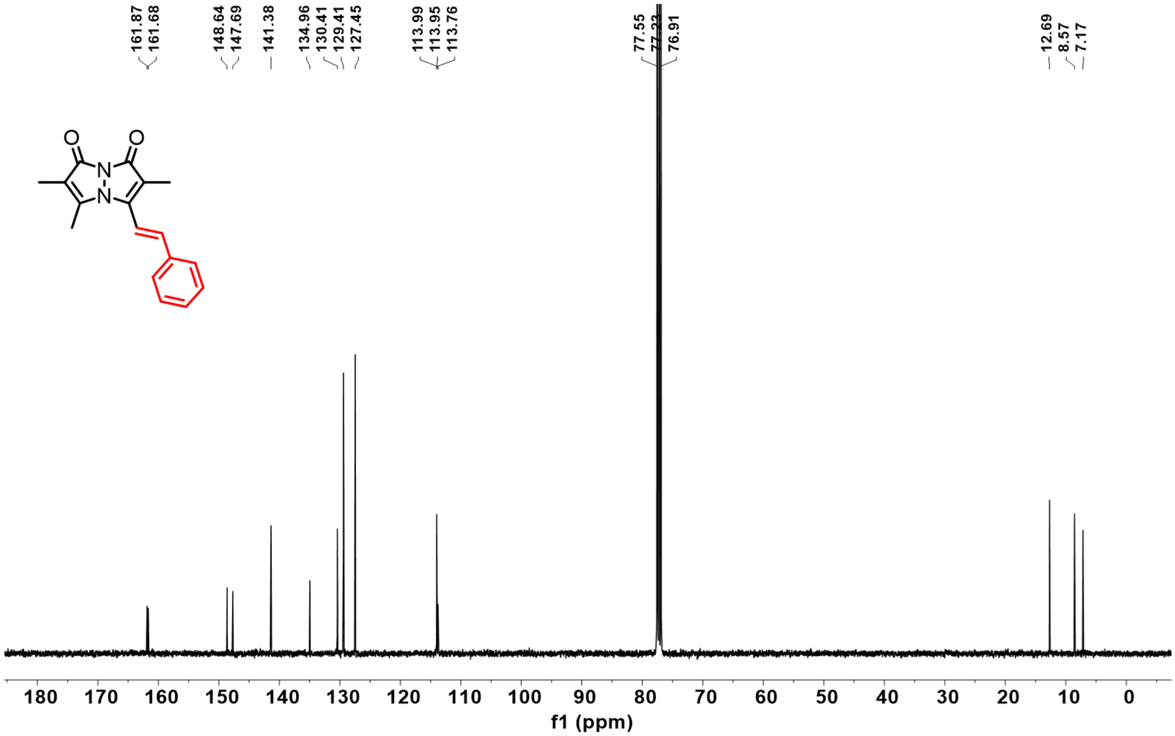

Fig. S33. ^{1}H NMR and ^{13}C NMR spectrum of **5d** in $CDCl_3$.

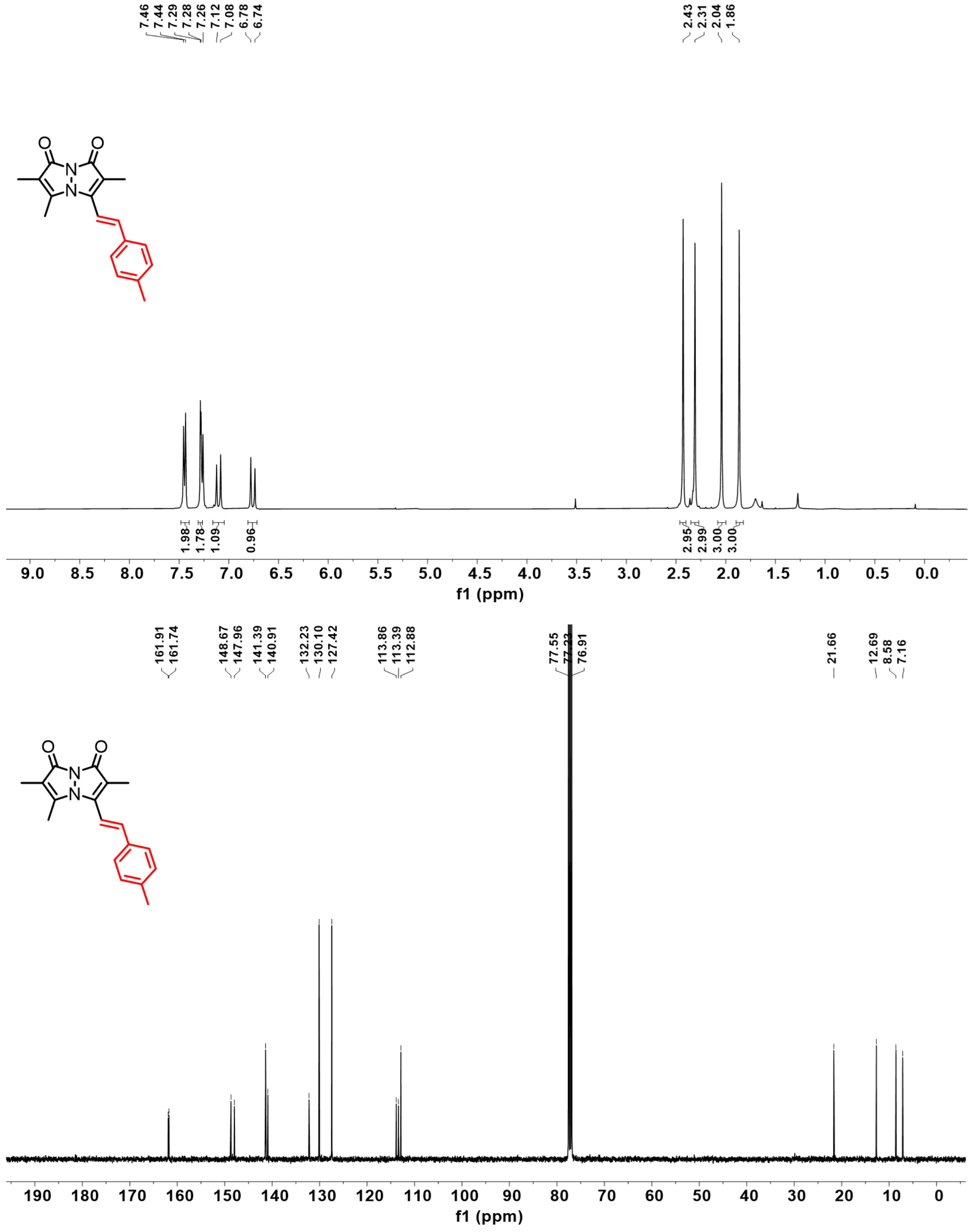

Fig. S34. ^{1}H NMR and ^{13}C NMR spectrum of **5e** in $CDCl_3$.

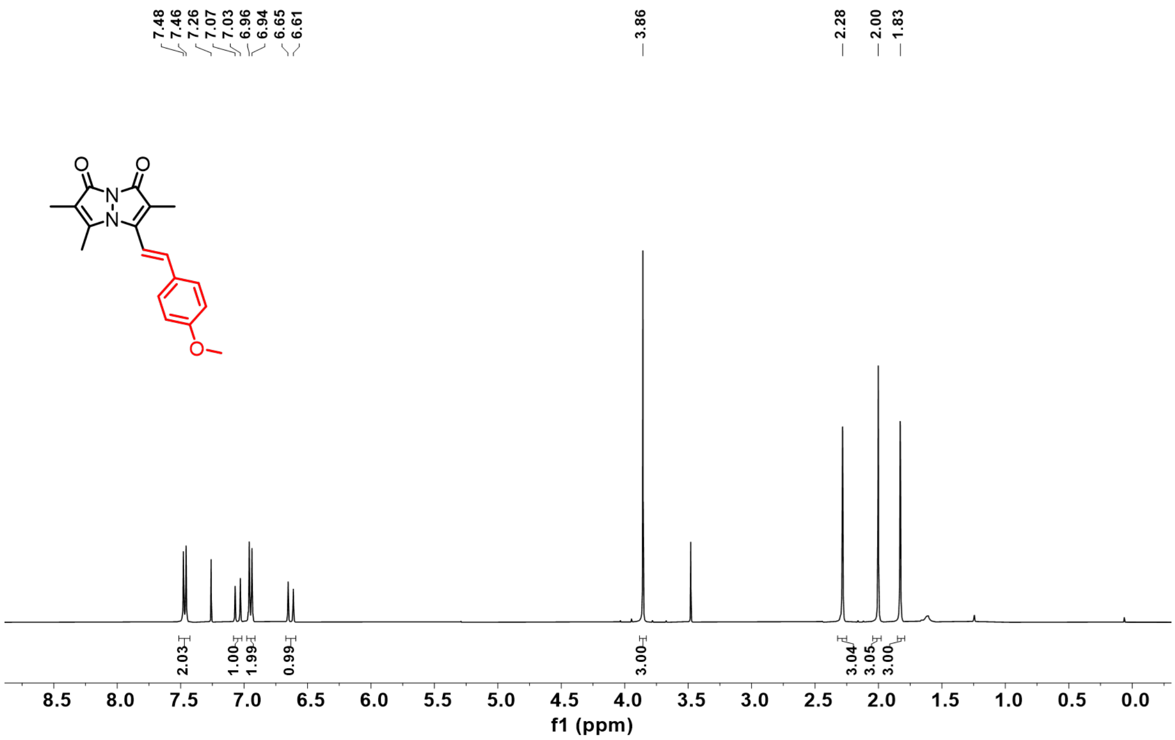

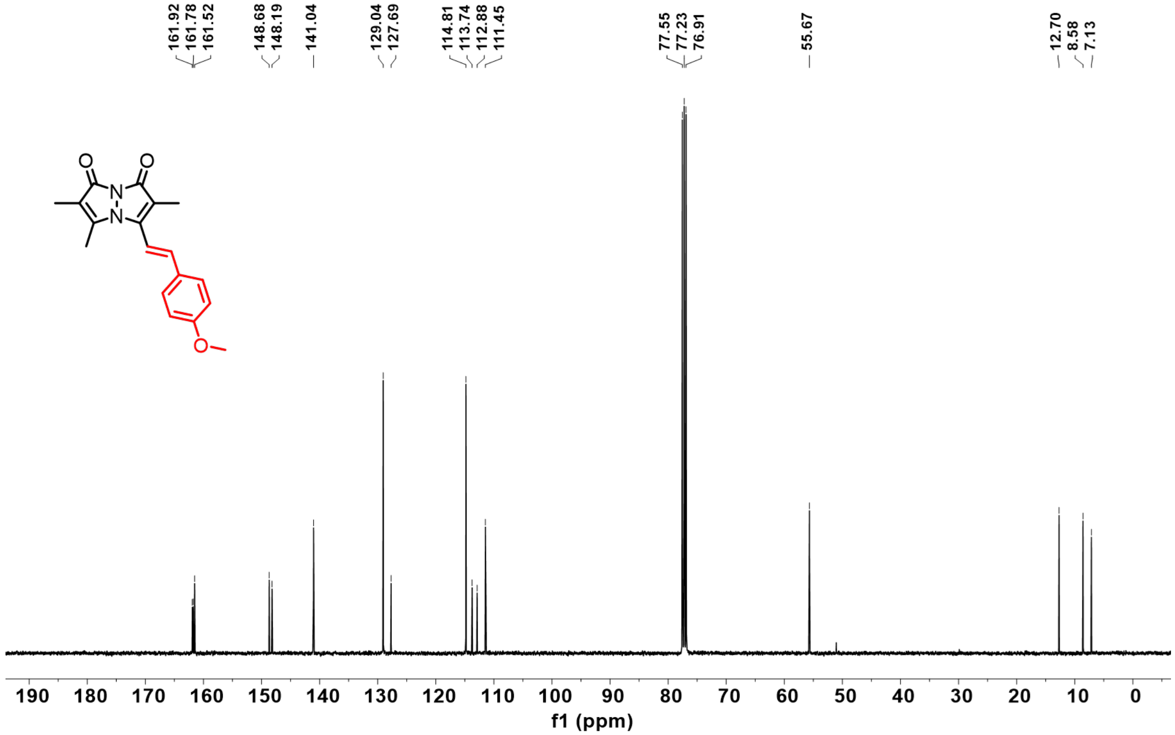

Fig. S35. ^{1}H NMR and ^{13}C NMR spectrum of **5f** in $CDCl_3$.

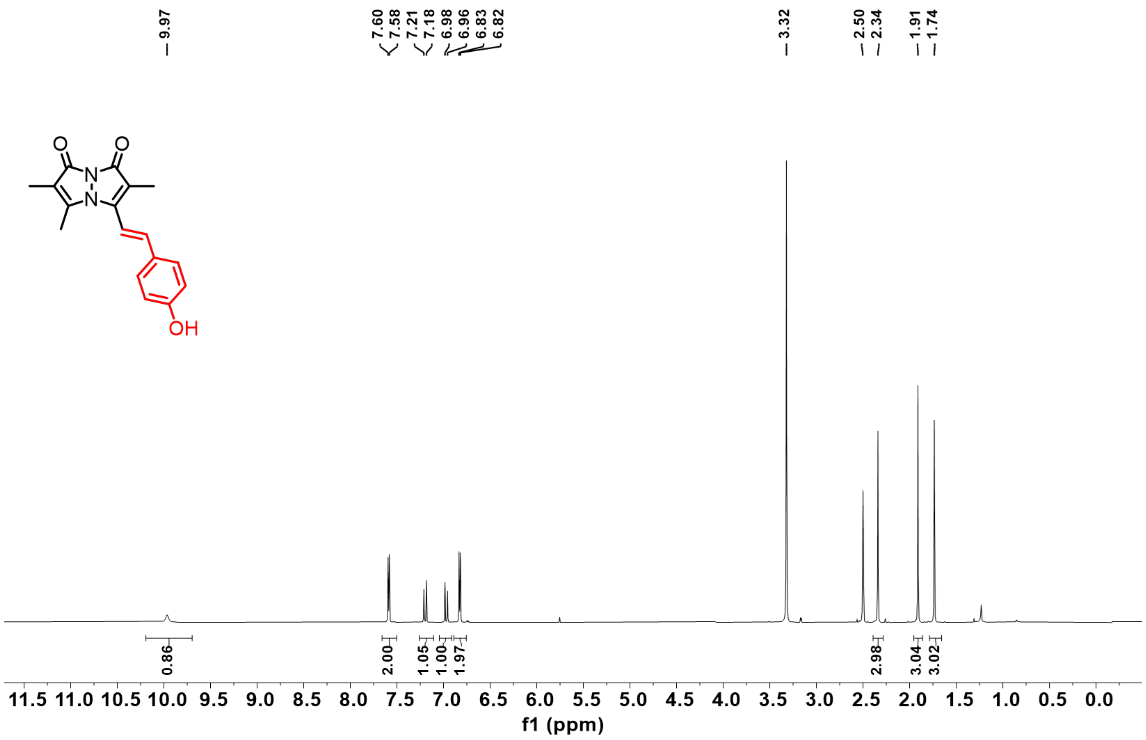

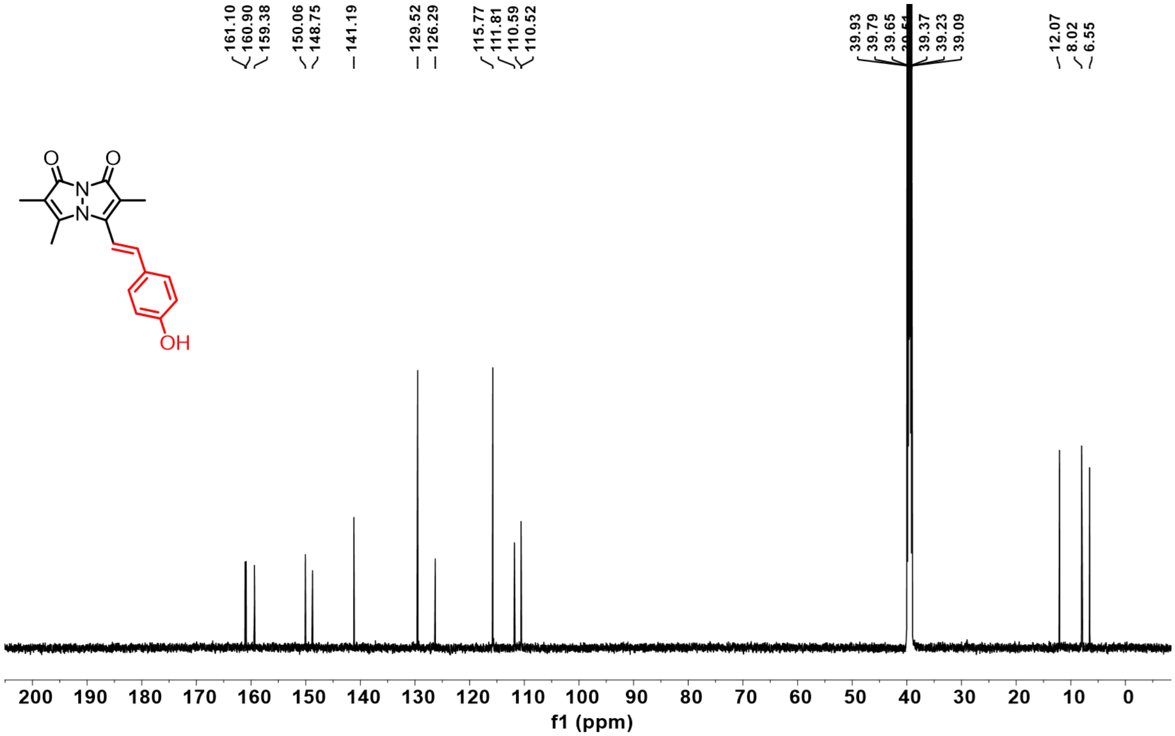

Fig. S36. ^{1}H NMR and ^{13}C NMR spectrum of **5h** in $CDCl_3$.

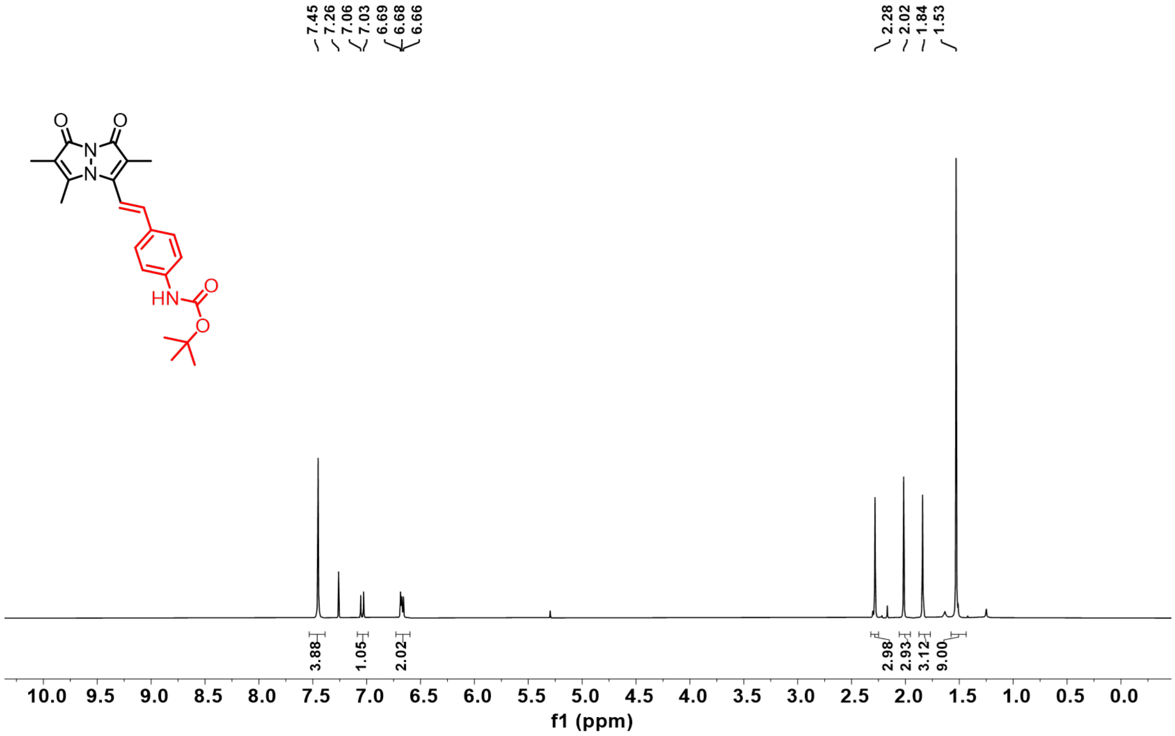

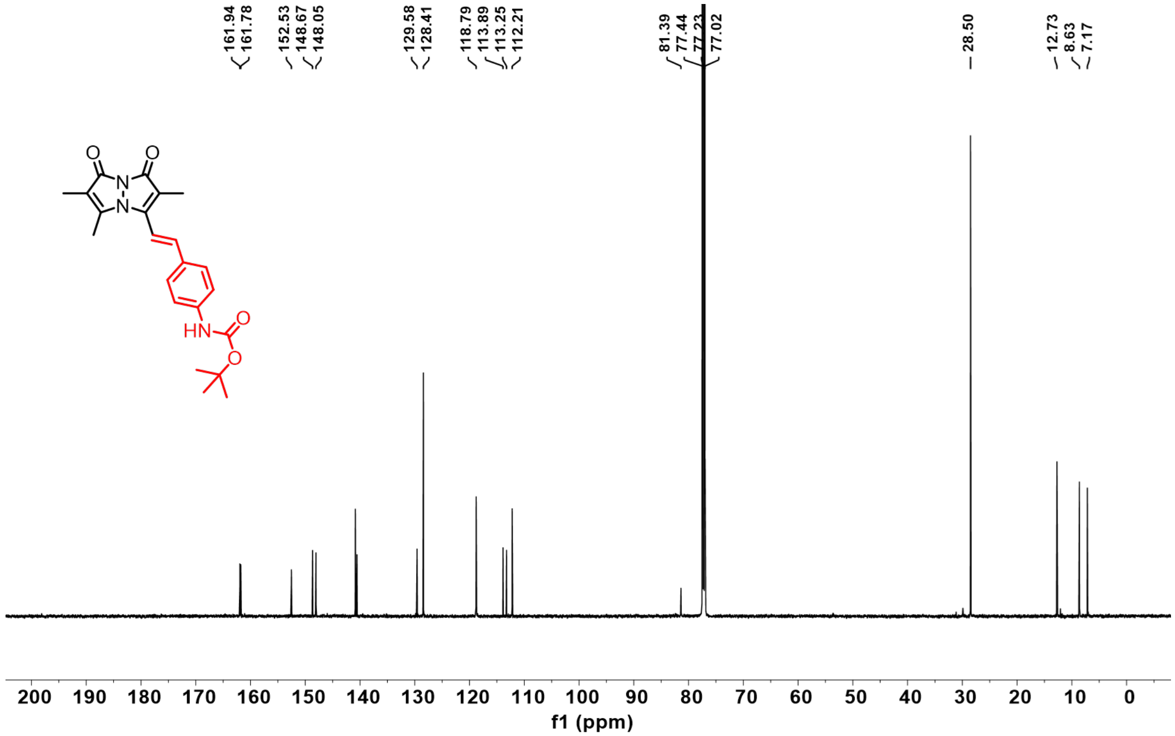

Fig. S37. ^{1}H NMR and ^{13}C NMR spectrum of **5i** in $CDCl_3$.

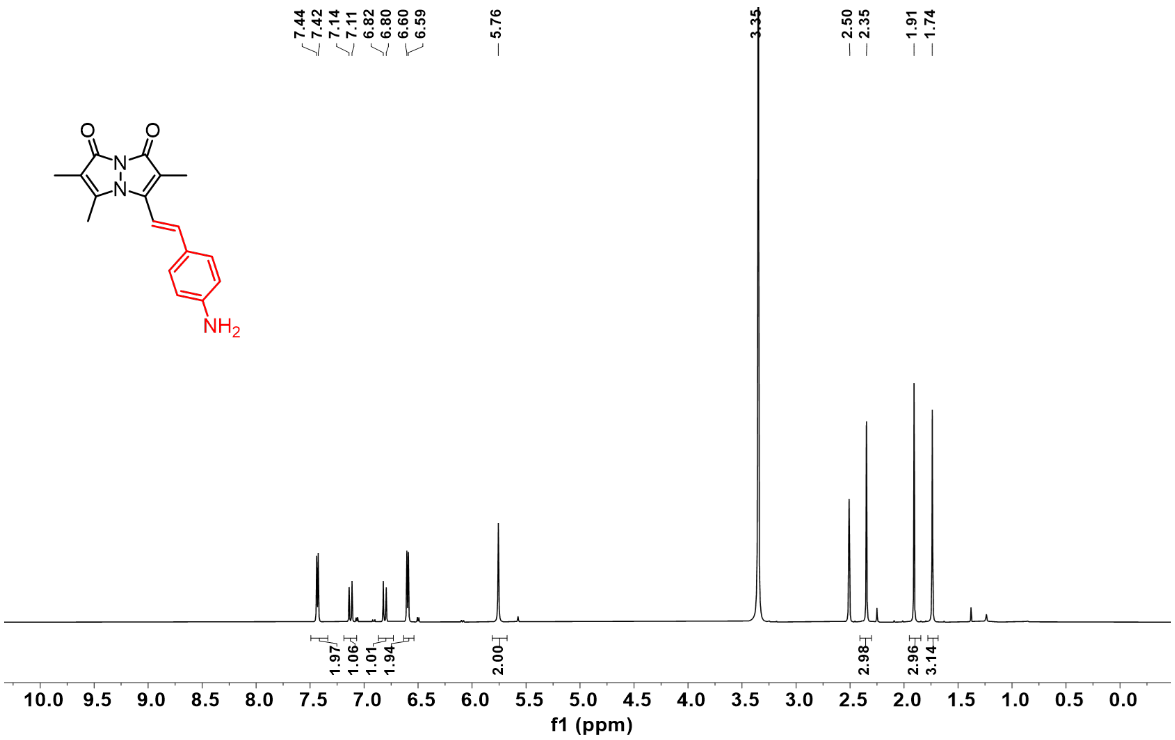

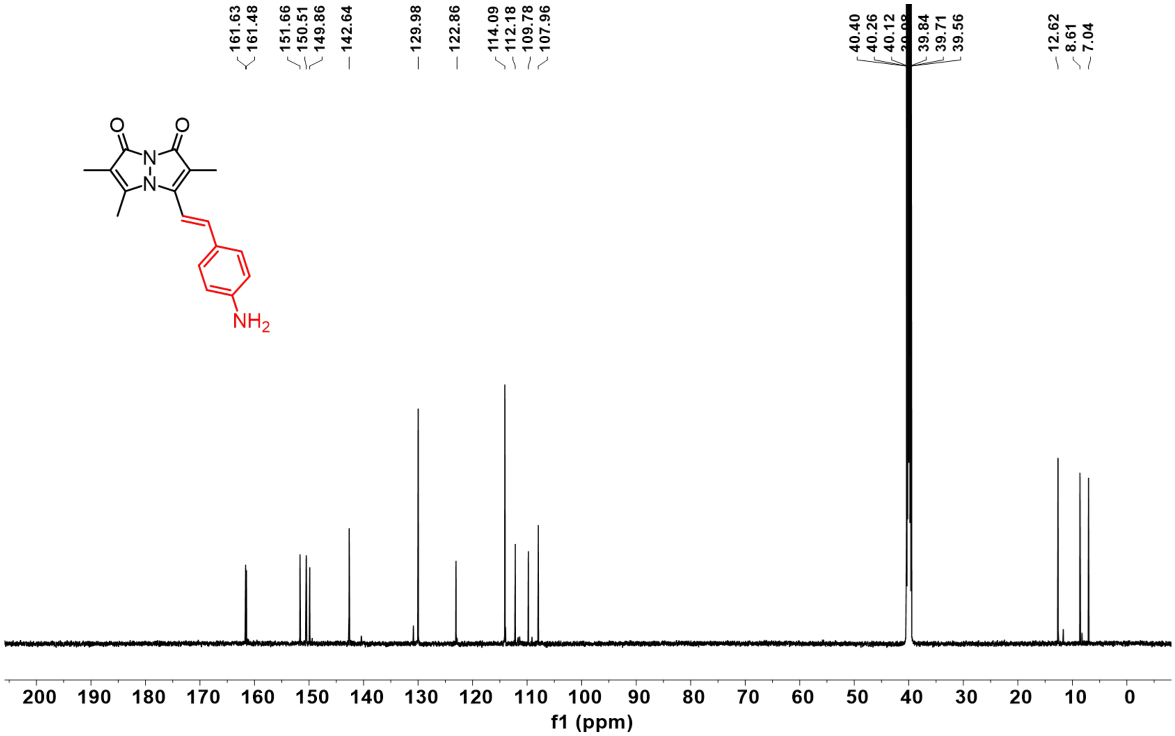

Fig. S38. ^{1}H NMR and ^{13}C NMR spectrum of **5j** in $CDCl_3$.

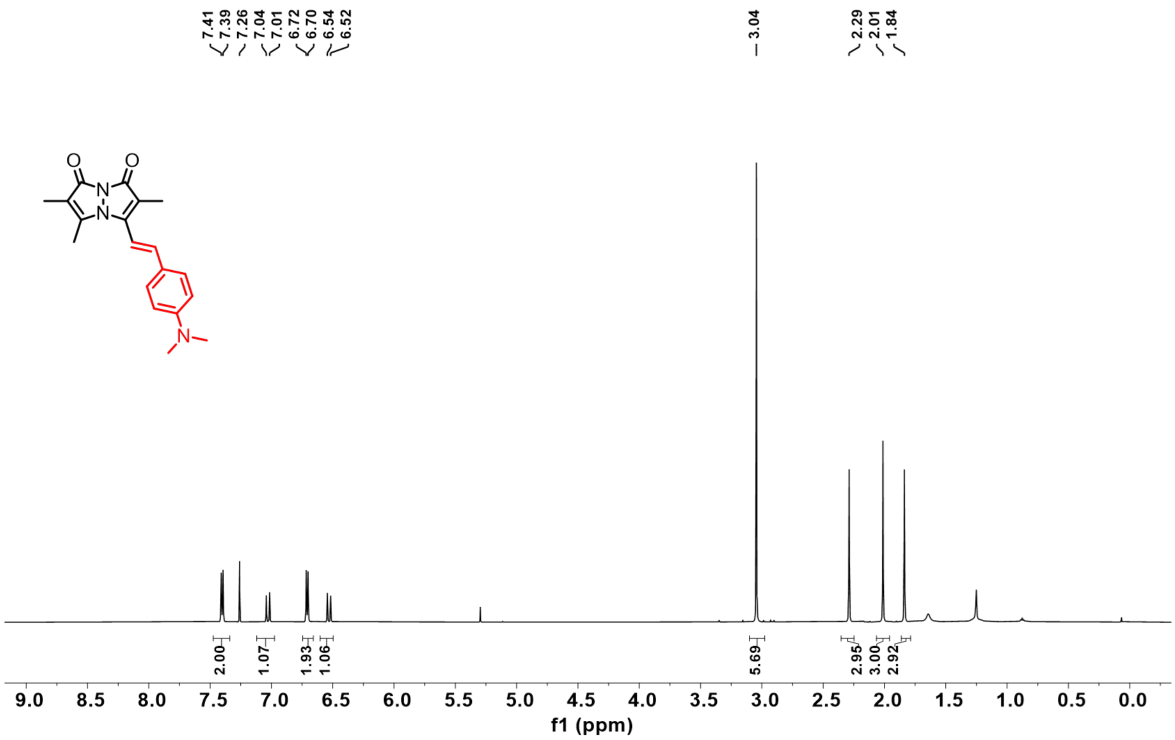

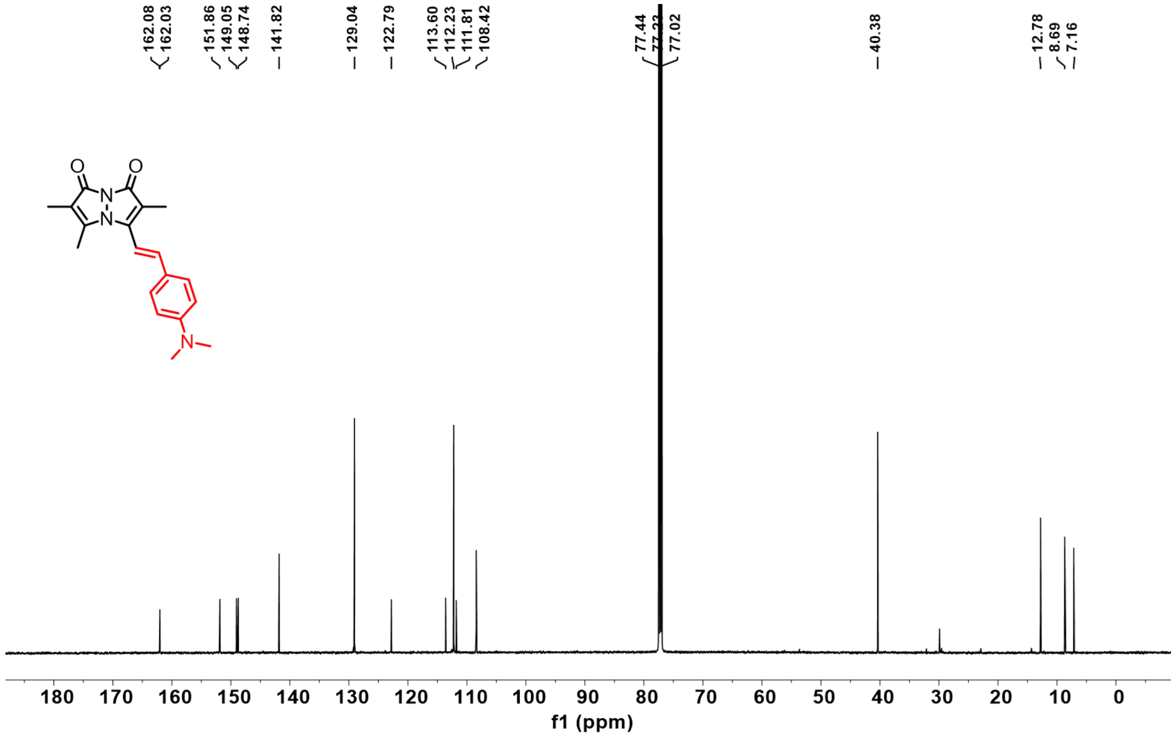

Fig. S39. ^{1}H NMR and ^{13}C NMR spectrum of **5k** in $CDCl_3$.

Cartesian coordinates of the optimized geometries: 5a

optimized at # **APFD/6-311+G (2d, p)** at ground state

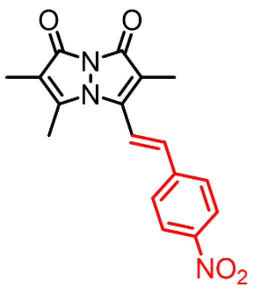

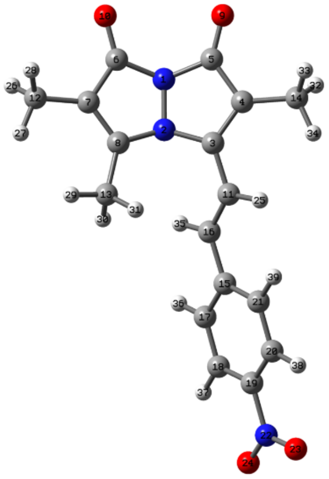

N	-3.579474	0.239347	-0.572001		C	2.833122	0.762288	0.763210
N	-2.299302	-0.179095	-0.279420		N	6.346465	-0.179185	0.021334
C	-1.565537	0.948845	0.101344		O	6.904811	0.459678	0.899523
C	-2.370043	2.04777	0.092737		O	6.934219	-0.879406	-0.788087
C	-3.696554	1.628687	-0.316770		H	0.248439	1.664982	0.987488
C	-4.497548	-0.789201	-0.239320		H	-4.721945	-3.673432	-0.185264
C	-3.678545	-1.868992	0.272888		H	-3.502670	-3.848146	1.080706
C	-2.386686	-1.454747	0.287703		H	-5.037600	-3.03964	1.425392
O	-4.714624	2.276812	-0.463436		H	-1.511011	-3.083121	1.301642
O	-5.702171	-0.711177	-0.385578		H	-0.554378	-2.49759	-0.061723
C	-0.138163	0.916306	0.304655		H	-0.592096	-1.576429	1.441256
C	-4.257531	-3.17677	0.671821		H	-2.135087	4.051378	-0.599358
C	-1.194392	-2.188545	0.768133		H	-2.688372	3.912896	1.064510
C	-2.020095	3.470545	0.321110		H	-0.993617	3.588994	0.667770
C	2.137264	0.054229	-0.227891		H	0.267664	-0.57914	-1.098809
C	0.689924	0.099489	-0.362846		H	2.335367	-1.297757	-1.891595
C	2.863078	-0.739992	-1.125457		H	4.805826	-1.430819	-1.746577
C	4.241194	-0.821114	-1.053632		H	4.751503	1.232565	1.609710
C	4.895858	-0.100635	-0.066021		H	2.296741	1.368714	1.483134
C	4.207909	0.690472	0.847159					

Cartesian coordinates of the optimized geometries: 5b

optimized at # **APFD/6-311+G (2d, p)** at ground state

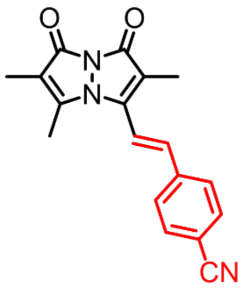

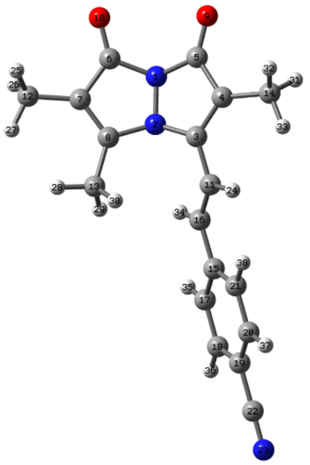

Atom	*X*	*Y*	*Z*		*Atom*	*X*	*Y*	*Z*
N	-3.175423	0.260862	-0.566947		C	4.616648	0.629971	0.863327
N	-1.896895	-0.170511	-0.285469		C	3.242428	0.715587	0.779611
C	-1.157479	0.944302	0.122826		C	6.742536	-0.238066	0.024200
C	-1.957814	2.046319	0.143090		N	7.891747	-0.310201	0.095217
C	-3.285923	1.644018	-0.275238		H	0.659186	1.626515	1.030180
C	-4.097905	-0.770661	-0.258242		H	-4.646372	-3.057872	1.354389
C	-3.284010	-1.866479	0.227124		H	-4.338909	-3.653211	-0.272395
C	-1.990056	-1.458914	0.250790		H	-3.116984	-3.866005	0.985267
O	-4.301799	2.299709	-0.404539		H	-1.121964	-3.116456	1.223149
O	-5.302436	-0.683402	-0.401663		H	-0.162738	-2.501284	-0.125440
C	0.269405	0.898653	0.326914		H	-0.195702	-1.617838	1.399826
C	-3.869206	-3.180533	0.595312		H	-1.735349	4.070576	-0.490987
C	-0.801166	-2.210297	0.712134		H	-2.250466	3.881592	1.180182
C	-1.600701	3.461198	0.408041		H	-0.566325	3.567162	0.734720
C	2.541104	0.035246	-0.226156		H	0.667988	-0.561324	-1.114802
C	1.093903	0.095273	-0.360914		H	2.731421	-1.281349	-1.917760
C	3.262673	-0.744463	-1.138830		H	5.189959	-1.441945	-1.779180
C	4.640341	-0.838321	-1.066582		H	5.152796	1.156947	1.643875
C	5.322094	-0.147649	-0.062807		H	2.710114	1.311865	1.511384

Cartesian coordinates of the optimized geometries: 5c

optimized at # **APFD/6-311+G (2d, p)** at ground state

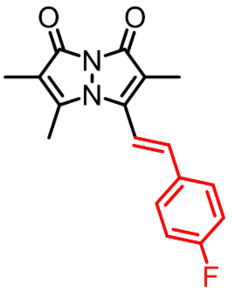

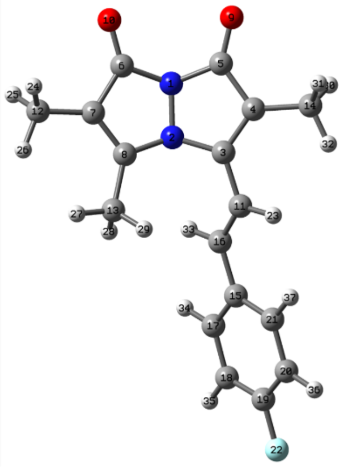

N	-2.940634	0.265317	-0.574339		C	4.863449	0.615525	0.850855
N	-1.662558	-0.162997	-0.284168		C	3.487470	0.720354	0.765164
C	-0.924636	0.954461	0.122747		F	6.867657	-0.310086	0.032808
C	-1.730303	2.053997	0.139873		H	0.889452	1.639890	1.032950
C	-3.053538	1.649989	-0.283024		H	-4.419956	-3.047326	1.348618
C	-3.862576	-0.765402	-0.266313		H	-4.096245	-3.651200	-0.271777
C	-3.050403	-1.858413	0.226952		H	-2.885225	-3.853401	0.997900
C	-1.756784	-1.448802	0.256245		H	-0.895841	-3.097266	1.250120
O	-4.070855	2.303377	-0.420497		H	0.069405	-2.500109	-0.102334
O	-5.067378	-0.681000	-0.415796		H	0.034083	-1.598097	1.411261
C	0.499815	0.907164	0.334622		H	-1.549150	4.086614	-0.483067
C	-3.635595	-3.171995	0.597277		H	-1.996761	3.880263	1.205419
C	-0.571043	-2.197833	0.729708		H	-0.330078	3.580946	0.692055
C	-1.375553	3.470289	0.404394		H	0.892251	-0.565592	-1.092101
C	2.769777	0.016419	-0.211649		H	2.936086	-1.362491	-1.854576
C	1.324526	0.094452	-0.344175		H	5.409747	-1.557231	-1.709364
C	3.477427	-0.805984	-1.096075		H	5.427051	1.154112	1.603992
C	4.857002	-0.923153	-1.025983		H	2.968499	1.354440	1.475065
C	5.525296	-0.205740	-0.050540					

Cartesian coordinates of the optimized geometries: 5d

optimized at # **APFD/6-311+G (2d, p)** at ground state

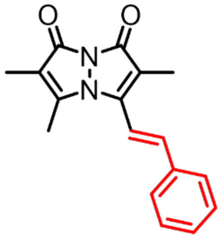

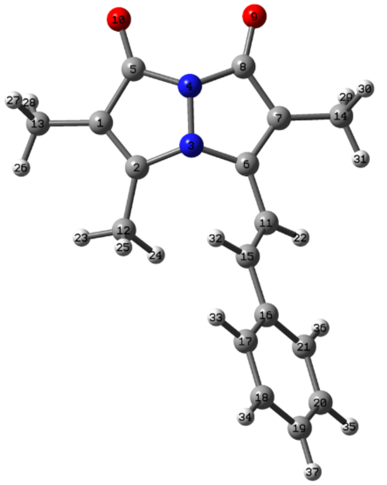

Atom	*X*	*Y*	*Z*		*Atom*	*X*	*Y*	*Z*
C	2.709914	-1.832463	0.225581		C	-5.246127	0.515995	0.848202
C	1.409734	-1.444149	0.255490		C	-3.870391	0.649007	0.767610
N	1.294170	-0.159992	-0.284690		H	-1.286086	1.601905	1.033804
N	2.564860	0.289121	-0.575629		H	0.576230	-3.107511	1.247925
C	3.503719	-0.726227	-0.268001		H	-0.377549	-1.623640	1.411811
C	0.538398	0.945285	0.122637		H	-0.399628	-2.523895	-0.102915
C	1.326058	2.057825	0.139010		H	2.578508	-3.829978	0.996711
C	2.655571	1.675352	-0.284636		H	3.785097	-3.607579	-0.273874
O	3.662362	2.344850	-0.422207		H	4.099800	-2.998366	1.346279
O	4.706860	-0.621797	-0.418015		H	1.576046	3.892986	1.192083
C	-0.885040	0.875175	0.335675		H	1.093914	4.084192	-0.488728
C	0.236614	-2.212701	0.729022		H	-0.093610	3.559635	0.710065
C	3.317091	-3.136151	0.595505		H	-1.256938	-0.605626	-1.088710
C	0.948475	3.468072	0.403993		H	-3.285927	-1.439487	-1.839931
C	-1.698027	0.049185	-0.341131		H	-5.739037	-1.674864	-1.695042
C	-3.142746	-0.049529	-0.205861		H	-5.795144	1.062227	1.608254
C	-3.838510	-0.888292	-1.084753		H	-3.357878	1.295592	1.471769
C	-5.217823	-1.020856	-1.003798		H	-7.004924	-0.419607	0.031143
C	-5.926887	-0.317912	-0.037317					

Cartesian coordinates of the optimized geometries: 5e

optimized at # **APFD/6-311+G (2d, p)** at ground state

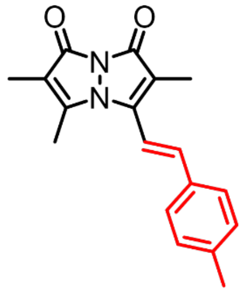

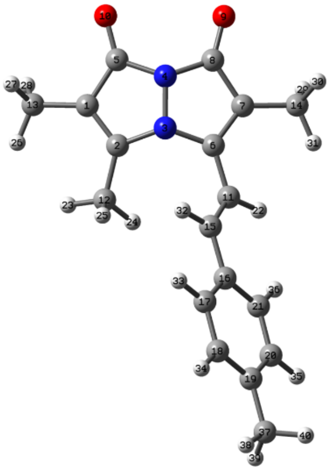

C	3.070078	-1.860922	0.227749		C	-3.474271	0.744994	0.739199
C	1.776834	-1.449510	0.255399		H	-0.867442	1.645650	1.024305
N	1.684888	-0.163980	-0.285366		H	0.913646	-3.094354	1.253370
N	2.964107	0.262556	-0.573975		H	-0.017275	-1.594474	1.405569
C	3.884083	-0.769163	-0.264711		H	-0.048066	-2.502711	-0.104128
C	0.947375	0.954511	0.120914		H	2.900993	-3.855508	0.999014
C	1.756077	2.052445	0.140984		H	4.113621	-3.655628	-0.269416
C	3.078435	1.647113	-0.280523		H	4.436673	-3.051889	1.351139
O	4.097234	2.298981	-0.416762		H	2.013296	3.873482	1.219264
O	5.089457	-0.686652	-0.412848		H	1.592913	4.088829	-0.475053
C	-0.476641	0.909291	0.330510		H	0.353889	3.582613	0.678824
C	0.590023	-2.197064	0.728502		H	-0.869912	-0.570536	-1.089569
C	3.652858	-3.175361	0.599012		H	-2.915764	-1.393706	-1.826325
C	1.403244	3.469171	0.406758		H	-5.364556	-1.584920	-1.686467
C	-1.302868	0.094741	-0.346286		H	-5.391145	1.202459	1.563025
C	-2.747060	0.020586	-0.217093		H	-2.959312	1.397568	1.436356
C	-3.457394	-0.818583	-1.080832		C	-7.051771	-0.307866	0.042536
C	-4.838882	-0.926145	-1.001731		H	-7.454403	-1.004901	-0.693987
C	-5.560168	-0.202665	-0.055847		H	-7.355856	-0.649544	1.036384
C	-4.848901	0.633241	0.813359		H	-7.524107	0.666022	-0.117445

Cartesian coordinates of the optimized geometries: 5f

optimized at # **APFD/6-311+G (2d, p)** at ground state

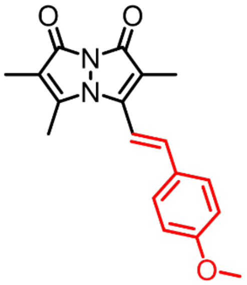

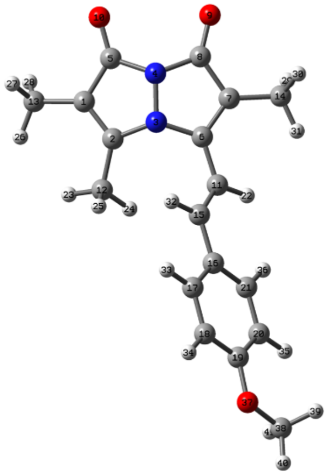

Atom	*X*	*Y*	*Z*		*Atom*	*X*	*Y*	*Z*
C	-3.446200	-1.838591	-0.276992		H	0.541438	1.643281	-0.943973
C	-2.148797	-1.439083	-0.271126		H	-1.279797	-3.080489	-1.269761
N	-2.057387	-0.159619	0.283364		H	-0.327862	-1.589703	-1.377411
N	-3.339375	0.276313	0.546031		H	-0.345832	-2.519092	0.119449
C	-4.261005	-0.743455	0.205758		H	-3.276860	-3.830202	-1.056259
C	-1.299839	0.955620	-0.096592		H	-4.524183	-3.624279	0.176994
C	-2.100339	2.060372	-0.129338		H	-4.792317	-3.006970	-1.448364
C	-3.434223	1.664419	0.258733		H	-2.294779	3.883461	-1.219885
O	-4.450579	2.324258	0.377669		H	-1.961162	4.096311	0.493879
O	-5.469138	-0.651025	0.325817		H	-0.666868	3.585073	-0.595354
C	0.126682	0.899003	-0.272905		H	0.469095	-0.606180	1.133040
C	-0.959107	-2.194329	-0.724530		H	2.484080	-1.476213	1.909800
C	-4.032471	-3.143902	-0.674231		H	4.942752	-1.698278	1.823043
C	-1.729215	3.475933	-0.377183		H	5.067252	1.154188	-1.386023
C	0.929241	0.064057	0.410714		H	2.650168	1.368280	-1.302024
C	2.371776	-0.029707	0.318174		O	6.505065	-0.484961	0.258520
C	3.051664	-0.896525	1.187834		C	7.318075	0.235923	-0.655869
C	4.424268	-1.027010	1.147049		H	7.222238	1.314051	-0.501505
C	5.171042	-0.290131	0.223998		H	8.341103	-0.070618	-0.450012
C	4.513242	0.576235	-0.657142		H	7.063477	-0.013603	-1.689484
C	3.135643	0.695831	-0.602681					

Cartesian coordinates of the optimized geometries: 5h

optimized at # **APFD/6-311+G (2d, p)** at ground state

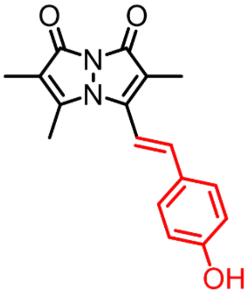

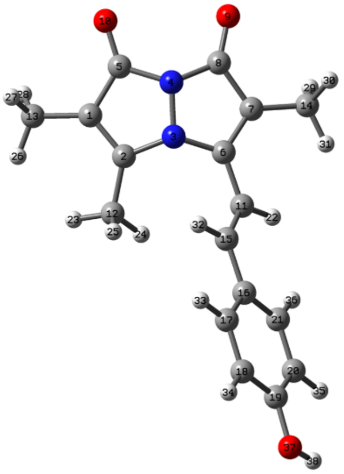

Atom	*X*	*Y*	*Z*		*Atom*	*X*	*Y*	*Z*
C	3.048615	-1.860249	0.230659		C	-4.866416	0.632282	0.816450
C	1.756340	-1.445499	0.260393		C	-3.492027	0.741684	0.737568
N	1.666039	-0.161822	-0.284406		H	-0.881487	1.659965	1.019156
N	2.945935	0.260908	-0.576889		H	0.893664	-3.082103	1.272191
C	3.863825	-0.771868	-0.266685		H	-0.036507	-1.580389	1.413439
C	0.931322	0.959592	0.120865		H	-0.069643	-2.500998	-0.088599
C	1.744827	2.054909	0.139832		H	2.877025	-3.850748	1.012066
C	3.064113	1.645978	-0.283817		H	4.083837	-3.660884	-0.263310
O	4.084731	2.294844	-0.423949		H	4.417059	-3.050497	1.352661
O	5.069440	-0.692943	-0.417456		H	1.990355	3.870842	1.231852
C	-0.491263	0.917355	0.331736		H	1.608725	4.095021	-0.470446
C	0.569702	-2.188832	0.740665		H	0.341734	3.592151	0.654227
C	3.629032	-3.174874	0.605095		H	-0.882794	-0.570788	-1.079316
C	1.396030	3.473095	0.404436		H	-2.922723	-1.402804	-1.820871
C	-1.318480	0.097349	-0.340095		H	-5.383442	-1.602108	-1.684893
C	-2.759586	0.018258	-0.214127		H	-5.416661	1.198589	1.562396
C	-3.468018	-0.827572	-1.078533		H	-2.982510	1.398119	1.434721
C	-4.843948	-0.945864	-1.010871		O	-6.894922	-0.361001	-0.030536
C	-5.550898	-0.213064	-0.061028		H	-7.273460	0.189750	0.664064

Cartesian coordinates of the optimized geometries: 5j

optimized at # **APFD/6-311+G (2d, p)** at ground state

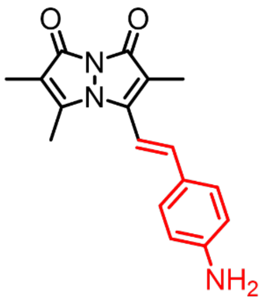

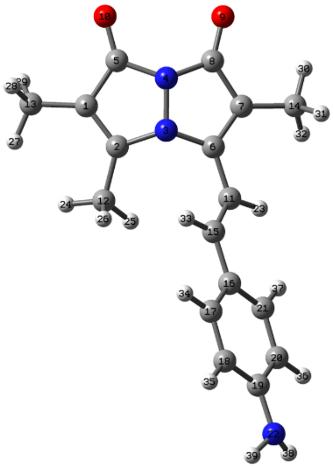

Atom	*X*	*Y*	*Z*		*Atom*	*X*	*Y*	*Z*
C	3.081408	-1.848353	0.231110		C	-3.498769	0.795146	0.687872
C	1.784247	-1.448140	0.256855		N	-6.943441	-0.261643	-0.015747
N	1.680041	-0.168352	-0.292572		H	-0.872871	1.702911	0.948720
N	2.956703	0.268569	-0.583102		H	0.940397	-3.075330	1.299254
C	3.884567	-0.752193	-0.267633		H	-0.020578	-1.588775	1.389855
C	0.933274	0.946284	0.109520		H	-0.015614	-2.549407	-0.088058
C	1.739843	2.049399	0.136995		H	2.930978	-3.834954	1.026755
C	3.060724	1.656705	-0.286326		H	4.131361	-3.643278	-0.254207
O	4.079603	2.310756	-0.424387		H	4.465014	-3.018386	1.356060
O	5.090449	-0.661258	-0.415815		H	2.249754	4.068494	0.528531
C	-0.485109	0.914330	0.312086		H	0.768395	3.883066	-0.415454
C	0.606097	-2.202366	0.740911		H	0.754963	3.549048	1.316988
C	3.675339	-3.155020	0.612375		H	-0.884451	-0.641861	-1.023831
C	1.353124	3.458646	0.406931		H	-2.921592	-1.525693	-1.708771
C	-1.319903	0.065624	-0.321896		H	-5.364564	-1.708015	-1.587095
C	-2.756177	0.004219	-0.205322		H	-5.419347	1.319993	1.463080
C	-3.468304	-0.895092	-1.013267		H	-2.991488	1.495993	1.342797
C	-4.842865	-1.002185	-0.948064		H	-7.398099	0.100343	0.808224
C	-5.574563	-0.203894	-0.056422		H	-7.383503	-1.087220	-0.391848
C	-4.869983	0.698068	0.762702					

Cartesian coordinates of the optimized geometries: 5k

optimized at **# APFD/6-311+G (2d, p)** at ground state

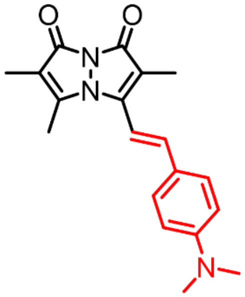

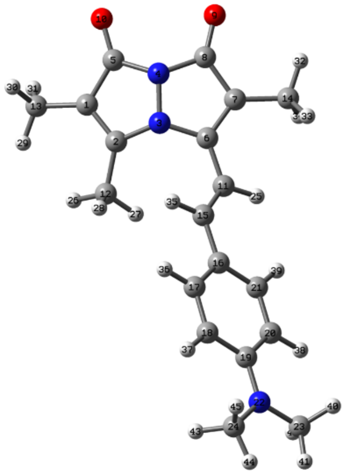

Atom	*X*	*Y*	*Z*		*Atom*	*X*	*Y*	*Z*
C	3.780493	-1.863968	0.305634		C	-6.952480	-1.139366	-0.783171
C	2.489388	-1.443184	0.315750		H	-0.120728	1.777481	0.870805
N	2.404138	-0.185737	-0.284632		H	1.622994	-3.007947	1.432773
N	3.687405	0.220452	-0.591535		H	0.680517	-1.506725	1.448625
C	4.599659	-0.800361	-0.235149		H	0.676654	-2.537892	0.019039
C	1.672505	0.955329	0.071119		H	3.598134	-3.817637	1.173089
C	2.497476	2.046437	0.057217		H	4.808214	-3.689795	-0.106473
C	3.811082	1.618517	-0.347719		H	5.141618	-3.010819	1.481717
O	4.839328	2.251983	-0.514302		H	2.957887	4.113926	-0.025743
O	5.807409	-0.733942	-0.385595		H	1.247881	3.751657	-0.307958
C	0.255392	0.954131	0.272521		H	1.909836	3.678620	1.325986
C	1.300877	-2.158864	0.831999		H	-0.167138	-0.658967	-0.988639
C	4.354214	-3.163573	0.738709		H	-2.217200	-1.556730	-1.622431
C	2.129321	3.470092	0.274325		H	-4.632990	-1.700659	-1.515759
C	-0.593752	0.088194	-0.323164		H	-4.654645	1.522154	1.364421
C	-2.027348	0.054964	-0.210908		H	-2.254114	1.641707	1.240173
C	-2.754717	-0.877105	-0.966653		H	-6.793898	1.784463	0.585150
C	-4.129023	-0.960762	-0.907576		H	-8.027714	0.533370	0.755897
C	-4.871541	-0.097370	-0.069125		H	-6.694139	0.607543	1.911714
C	-4.137743	0.841757	0.699983		H	-6.653658	-2.161949	-0.529588
C	-2.766543	0.908092	0.626064		H	-8.015547	-1.038595	-0.579201
N	-6.226296	-0.163696	0.000723		H	-6.796379	-0.990993	-1.857035
C	-6.964960	0.738093	0.858599					

REFERENCES

1. Kosower, E. M.; Pazhenchevsky, B. Synthesis and Properties of syn- and anti- 1,5 Diazabicyclo[3.3.0loctadienediones (9, l0-Dioxabimanes). *J. Am. Chem. Soc*. **1980**, *102*, 15, 4983–4993.

2. Ishita Neogi, Partha J. Das, and Flavio Grynszpan. Dihalogen and Solvent-Free Preparation of syn-Bimane. *Synlett* **2018**, *29*, 1043–1046.

3. Gaussian 16, Revision C.01, Frisch, M. J.; Trucks, G. W.; Schlegel, H. B.; Scuseria, G. E.; Robb, M. A.; Cheeseman, J. R.; Scalmani, G.; Barone, V.; Petersson, G. A.; Nakatsuji, H.; Li, X.; Caricato, M.; Marenich, A. V.; Bloino, J.; Janesko, B. G.; Gomperts, R.; Mennucci, B.; Hratchian, H. P.; Ortiz, J. V.; Izmaylov, A. F.; Sonnenberg, J. L.; Williams-Young, D.; Ding, F.; Lipparini, F.; Egidi, F.; Goings, J.; Peng, B.; Petrone, A.; Henderson, T.; Ranasinghe, D.; Zakrzewski, V. G.; Gao, J.; Rega, N.; Zheng, G.; Liang, W.; Hada, M.; Ehara, M.; Toyota, K.; Fukuda, R.; Hasegawa, J.; Ishida, M.; Nakajima, T.; Honda, Y.; Kitao, O.; Nakai, H.; Vreven, T.; Throssell, K.; Montgomery, J. A., Jr.; Peralta, J. E.; Ogliaro, F.; Bearpark, M. J.; Heyd, J. J.; Brothers, E. N.; Kudin, K. N.; Staroverov, V. N.; Keith, T. A.; Kobayashi, R.; Normand, J.; Raghavachari, K.; Rendell, A. P.; Burant, J. C.; Iyengar, S. S.; Tomasi, J.; Cossi, M.; Millam, J. M.; Klene, M.; Adamo, C.; Cammi, R.; Ochterski, J. W.; Martin, R. L.; Morokuma, K.; Farkas, O.; Foresman, J. B.; Fox, D. J. Gaussian, Inc., Wallingford CT, 2016.

4. Lougee, M. G.; Pagar, V. V.; Kim, H. J.; Pancoe, S. X.; Chia, W. K.; Mach, R. H.; Garcia, B. A.; Petersson, E. J. Harnessing the Intrinsic Photochemistry of Isoxazoles for the Development of Chemoproteomic Crosslinking Methods. *Chem. Commun*., **2022**, *58*, 9116–9119.

5. Fung, H. Y. J.; McKibben, K. M.; Ramirez, J.; Gupta, K.; Rhoades, E. Structural Characterization of Tau in Fuzzy Tau: Tubulin Complexes. *Structure* **2020**, *28* (3), 378-384.

6. Stine, W. B.; Jungbauer, L.; Yu, C.; Ladu, M. J. Preparing Synthetic Aβ in Different Aggregation States. *Methods Mol. Biol*. **2011**, *670*, 13–32.

7. Ferrie, J. J.; Lengyel-Zhand, Z.; Janssen, B.; Lougee, M. G.; Giannakoulias, S.; Hsieh, C. J.; Pagar, V. V.; Weng, C. C.; Xu, H.; Graham, T. J. A.; Lee, V. M. Y.; Mach, R. H.; Petersson, E. J. Identification of a Nanomolar Affinity A-Synuclein Fibril Imaging Probe by Ultra-High Throughput: In Silico Screening. *Chem. Sci*. **2020**, 11 (47), 12746–12754.

8. Haney, C. M.; Cleveland, C. L.; Wissner, R. F.; Owei, L.; Robustelli, J.; Daniels, M. J.; Canyurt, M.; Rodriguez, P.; Ischiropoulos, H.; Baumgart, T.; Petersson, E. J., Site-Specific Fluorescence Polarization for Studying the Disaggregation of α-Synuclein Fibrils by Small Molecules. *Biochemistry* **2017,** *56* (5), 683-691.

9. Marotta, N. P.; Ara, J.; Uemura, N.; Lougee, M. G.; Meymand, E. S.; Zhang, B.; Petersson, E. J.; Trojanowski, J. Q.; Lee, V. M. Y., Alpha-synuclein from patient Lewy bodies exhibits distinct pathological activity that can be propagated in vitro. *Acta Neuropathol. Commun.* **2021,** *9* (1), 188.

10. Volpicelli-Daley, L. A.; Luk, K. C.; Patel, T. P.; Tanik, S. A.; Riddle, D. M.; Stieber, A.; Meaney, D. F.; Trojanowski, J. Q.; Lee, V. M.-Y. Exogenous α-Synuclein Fibrils Induce Lewy Body Pathology Leading to Synaptic Dysfunction and Neuron Death. *Neuron* **2011**, 72 (1), 57–71.
